# Liposomal Binuclear Ir(III)–Cu(II) Coordination Compounds with Phosphino-Fluoroquinolone Conjugates for Human Prostate Carcinoma Treatment

**DOI:** 10.1021/acs.inorgchem.2c03015

**Published:** 2022-11-16

**Authors:** Urszula K. Komarnicka, Sandra Kozieł, Barbara Pucelik, Agata Barzowska, Miłosz Siczek, Magdalena Malik, Daria Wojtala, Alessandro Niorettini, Agnieszka Kyzioł, Victor Sebastian, Pavel Kopel, Stefano Caramori, Alina Bieńko

**Affiliations:** † Faculty of Chemistry, University of Wroclaw, Joliot-Curie 14, 50-383 Wroclaw, Poland; ‡ Małopolska Centre of Biotechnology, Jagiellonian University, Gronostajowa 7A, 30-387 Krakow, Poland; § Faculty of Chemistry, Wroclaw University of Science and Technology, Wybrzeże Wyspiańskiego 27, 50-370 Wroclaw, Poland; ∥ Department of Chemical, Pharmaceutical, and Agricultural Sciences, University of Ferrara, Via L. Borsari 46, 44121 Ferrara, Italy; ⊥ Faculty of Chemistry, Jagiellonian University, Gronostajowa 2, 30-387 Kraków, Poland; # Department of Chemical Engineering and Environmental Technologies, University of Zaragoza, Campus Río Ebro-Edificio I+D, Mariano Esquillor S/N, 50018 Zaragoza, Spain; ∇ Networking Research Center on Bioengineering, Biomaterials and Nanomedicine, CIBER-BBN, 28-029 Madrid, Spain; ○ Instituto de Nanociencia y Materiales de Aragón (INMA), CSIC-Universidad de Zaragoza, 50009 Zaragoza, Spain; ◆ Department of Inorganic Chemistry, Faculty of Science, Palacky University, 17. listopadu 12, CZ-771 46 Olomouc, Czech Republic

## Abstract

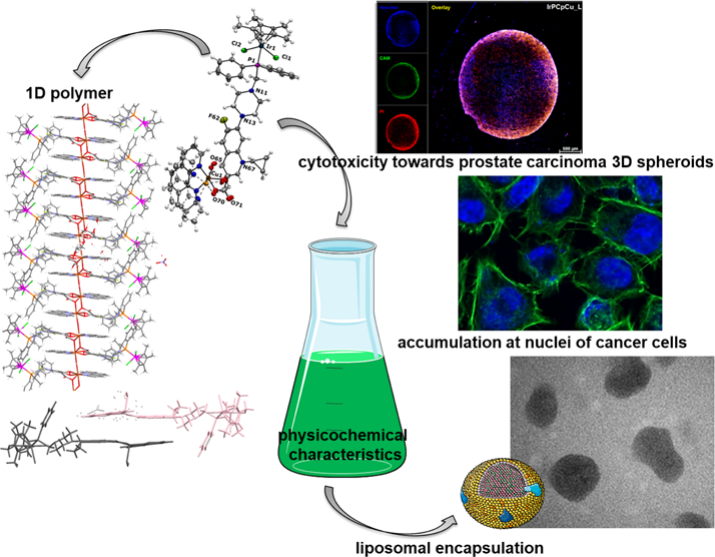

Novel heteronuclear Ir^III^–Cu^II^ coordination compounds ([Ir(η^5^-Cp*)Cl_2_Pcfx-Cu(phen)](NO_3_)·1.75(CH_3_OH)·0.75(H_2_O) (**1**), [Ir(η^5^-Cp*)Cl_2_Pnfx-Cu(phen)](NO_3_)·1.75(CH_3_OH)·0.75(H_2_O) (**2**), [Ir(η^5^-Cp*)Cl_2_Plfx-Cu(phen)](NO_3_)·1.3(H_2_O)·1.95(CH_3_OH) (**3**), [Ir(η^5^-Cp*)Cl_2_Psfx-Cu(phen)] (**4**)) bearing phosphines derived from fluoroquinolones, namely, sparfloxacin (Hsfx), ciprofloxacin (Hcfx), lomefloxacin (Hlfx), and norfloxacin (Hnfx), have been synthesized and studied as possible anticancer chemotherapeutics. All compounds have been characterized by electrospray ionization mass spectrometry (ESI-MS), a number of spectroscopic methods (*i*.*e*., IR, fluorescence, and electron paramagnetic resonance (EPR)), cyclic voltammetry, variable-temperature magnetic susceptibility measurements, and X-ray diffractometry. The coordination geometry of Ir^III^ in all complexes adopts a characteristic piano-stool geometry with the η^5^-coordinated and three additional sites occupied by two chloride and phosphine ligands, while Cu^II^ ions in complexes **1** and **2** form a distorted square-pyramidal coordination geometry, and in complex **3**, the coordination geometry around Cu^II^ ions is a distorted octahedron. Interestingly, the crystal structure of [Ir(η^5^-Cp*)Cl_2_Plfx-Cu(phen)] features the one-dimensional (1D) metal–organic polymer. Liposomes loaded with redox-active and fluorescent [Ir(η^5^-Cp*)Cl_2_Pcfx-Cu(phen)] (**1L**) have been prepared to increase water solubility and minimize serious systemic side effects. It has been proven, by confocal microscopy and an inductively coupled plasma mass spectrometry (ICP-MS) analysis, that the liposomal form of compound **1** can be effectively accumulated inside human lung adenocarcinoma and human prostate carcinoma cells with selective localization in nuclei. A cytometric analysis showed dominance of apoptosis over the other cell death types. Furthermore, the investigated nanoformulations induced changes in the cell cycle, leading to S phase arrest in a dose-dependent manner. Importantly, *in vitro* anticancer action on three-dimensional (3D) multicellular tumor spheroids has been demonstrated.

## Abstract 2

Heteronuclear Ir^III^−Cu^II^ coordination compounds bearing phosphines derived from fluoroquinolones have been synthesized and characterized by strong fluorescence in solution, redox activity, and magnetic properties. Liposomes loaded with selected compounds revealed their high anticancer potential toward human prostate carcinoma cells simultaneously with low toxicity against healthy cells. A first insight into the action mode revealed that the liposomal complex effectively accumulates inside carcinoma cells with selective colocalization in nuclei causing apoptotic cell death.

## Introduction

Some of the most life-threatening diseases in the whole world are undeniably cancer diseases.^1^ According to the Global Cancer Observatory, over 9 million deaths were caused by cancer diseases in 2020 and around 19 million new cases have been reported.^2^ Currently, the most common metal-based therapeutics in chemotherapy are those containing Pt(II) ions.^3^ The effectiveness of drugs is still inhibited by clinical problems, for example, a limited spectrum of activity, high toxicity causing side effects, and acquired or intrinsic resistance.^4^

One of the approaches leading to overcoming these limitations is to find inspiration in the activity of novel heteronuclear complexes.^5^ When we incorporate two various cytotoxic metals into the same molecule, significant improvement in their antitumor activity can be noticed. This phenomenon can be explained by the interaction of different metals with multiple biological targets or by the improved chemicophysical properties of the resulting heteronuclear complexes. Therefore, in some cases where a specific type of cancer becomes resistant toward one of the metals, the second or third metal might still exhibit some cytotoxic activity. Through the years, several binuclear complexes have been synthesized and tested and some noticeable results have been described: for example, Au(I)–Ag(I), Au(I)–Pt(II), Cu(II)–Na(I), Au(I)–Ti(IV), and Ru(II)–Pt(IV).^5−10^ Patra and co-workers synthesized a heteronuclear complex Ru–Ir with the formula [(ppy)_2_Ir(μ-phpy)Ru(p-cym)Cl](PF_6_)_2_. The study showed that this complex was significantly more active than its mononuclear analogue as well as cisplatin against cell lines such as MCF7, SKOV3, and PC3. Moreover, the results show that these complexes induce autophagy in cancer cells.^11^ Zhu and co-workers patented a group of platinum–ruthenium inorganic compounds named Ruthplatins with increased or comparable activity to cisplatin toward a number of cell lines (even toward cisplatin-resistant cell lines). The introduction of the ruthenium center led to increased complex toxicity against healthy MRC-5 lung fibroblast cells compared to the cisplatin control.^12^

Consequently, similar to this approach, in our research, we decided to incorporate two metal ions in our research: copper(II) and iridium(III). It was proved that through many processes, such as DNA damage or generation of reactive oxygen species (ROS), Cu(II) complexes could effectively induce cancer cell death. In addition, the superiority of these substances is also represented by the fact that Cu(II) ions are already present in the human body, limiting the possibility of excessive immunological system response.^13^ Additionally, the introduction of a transition metal with unpaired electrons can also endow the obtained complexes with additional magnetic functionality such as SIM, SMM, or superparamagnetic behavior, which show slow relaxation of magnetization.^14−24^

In the last few years, many scientists around the world have been working on the development of iridium(III) organometallic complexes and have succeeded in proving that iridium(III) inorganic compounds can replace platinum-based drugs.^3,25−33^ These complexes have unique properties such as potential redox activity, a wide range of ligand exchange rates and universal structure, greater cellular uptake efficiency, large stokes shifts, and lower toxic effect.^3,25−27,31,32^ Various mechanisms are responsible for the antitumor activity of iridium(III) compounds, such as protein activity inhibition, catalysis of cellular redox reactions, and damage of specific subcellular organelles. These amazing properties of iridium(III) complexes make them a rising star among new, potential anticancer agents.^3,25−33^

Additionally, to circumvent the previously mentioned side effects, our potential bioactive molecules will be loaded in liposomes. Liposomal technology has attracted great interest in nanomedicine because of liposomes’ low toxicity, biodegradability, and efficient cellular uptake.^34−36^ Liposomes have already been described as great transporters of both hydrophilic and hydrophobic molecules to cancer cells. Additionally, those nanoparticles could also provide (i) protection against the speciation of complexes in the bloodstream as well as (ii) the possibility of targeting in solid tumors.^36,37^

The research described herein on anticancer compounds is a continuation of our ongoing project in which phosphine ligands (Ph_2_P-CH_2_-FQ, FQ: fluoroquinolone antibiotic) bearing fluoroquinolones, namely, sparfloxacin (Hsfx), ciprofloxacin (Hcfx), lomefloxacin (Hlfx), and norfloxacin (Hnfx), were coordinated to various metal centers: Cu(I), Cu(II), Ru(II), and Ir(III).^4,38−42^ The results described above have confirmed that this mononuclear complex with phosphine is an excellent choice for the design of new biological agents. Therefore, we decided to extend our studies. Here, we investigate the dual nature of the iridium(III)–copper(II) ([Ir(η^5^-Cp*)Cl_2_Pcfx-Cu(phen)](NO_3_)·1.75(CH_3_OH)·0.75(H_2_O) (**1**), [Ir(η^5^-Cp*)Cl_2_Pnfx-Cu(phen)](NO_3_)·1.75(CH_3_OH)·0.75(H_2_O) (**2**), [Ir(η^5^-Cp*)Cl_2_Plfx-Cu(phen)](NO_3_)·1.3(H_2_O)·1.95(CH_3_OH) (**3**), [Ir(η^5^-Cp*)Cl_2_Psfx-Cu(phen)] (**4**)). The coordination of two different metal ions would significantly broaden the scope of their action mode with cells through different cytotoxic mechanisms. To take the above issues into account, first, their physicochemical properties have been determined using X-ray diffraction, elemental analysis, cyclic voltamperometry, mass spectrometry (electrospray ionization mass spectrometry (ESI-MS)), spectroscopic techniques, and variable-temperature magnetic susceptibility measurements. The cytotoxic effect of the compounds was assessed *in vitro* toward lung, breast, melanoma, and prostate tumor cell lines and one nontumor human embryonic kidney cell line. Based on the abovementioned findings, in this paper we presented a preclinical investigation into the therapeutic potential of complex IrPcfxCu encapsulated inside liposomes toward three-dimensional (3D) lung and prostate cancer cell cultures. Additionally, a working mechanism for these new inorganic compounds has been proposed and explained.

## Results and Discussion

### Synthesis

**Scheme 1:**
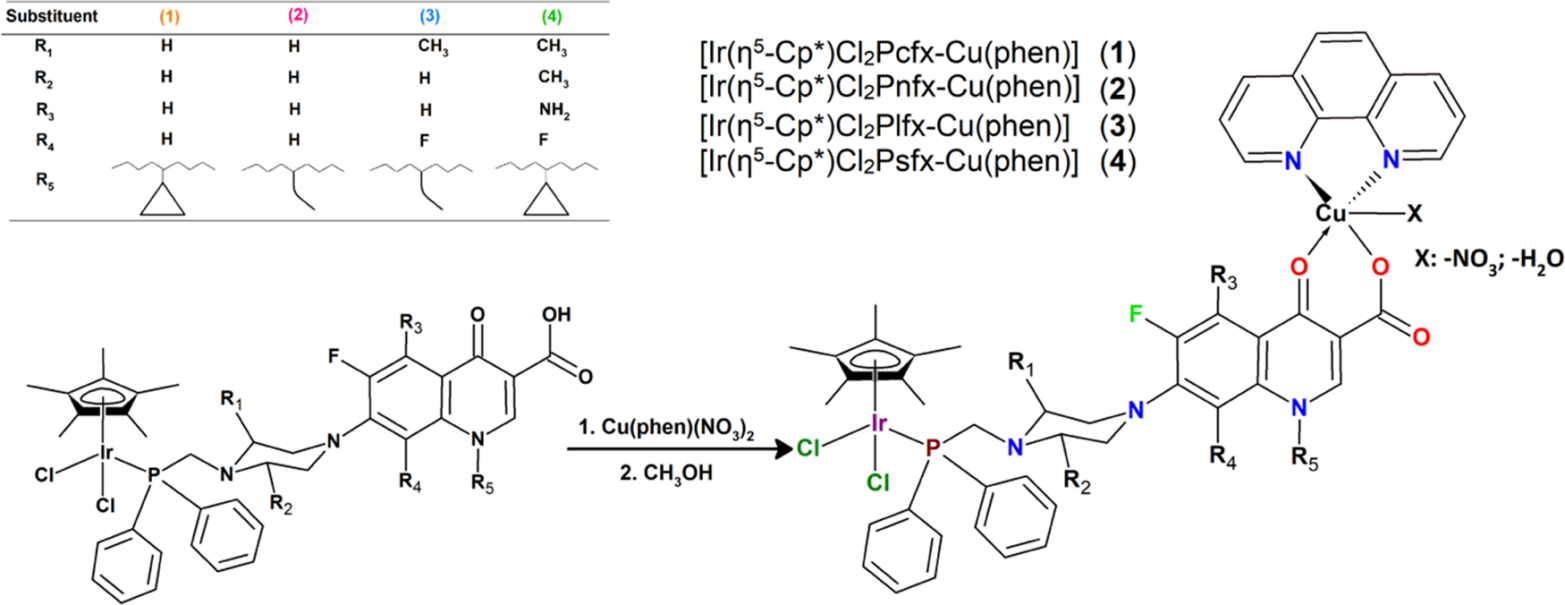
Schematic Illustration of the Inorganic Compound Structures and Synthetic Routes The solvent molecules have been omitted for clarity.

The four new heteronuclear complexes Ir^III^/Cu^II^ were synthesized (Scheme 1) by stirring [Cu(phen)(NO_3_)_2_] with 1 equiv of IrFQ ([Ir(η^5^-Cp*)Cl_2_PFQ]) at room temperature, previously reported by us in a recent study.^27^ Binuclear complexes are soluble in CH_3_OH, dimethyl sulfoxide (DMSO), and CH_2_Cl_2_ and insoluble in water. However, they can be solubilized in water containing 2% DMSO. The synthesis of mononuclear IrFQ complexes (incl.) was carried out under a nitrogen environment using Schlenk techniques. The chemical structures of studied Ir(III)/Cu(II) complexes are presented in Scheme 1.

### Structural Features

**Figure 1:**
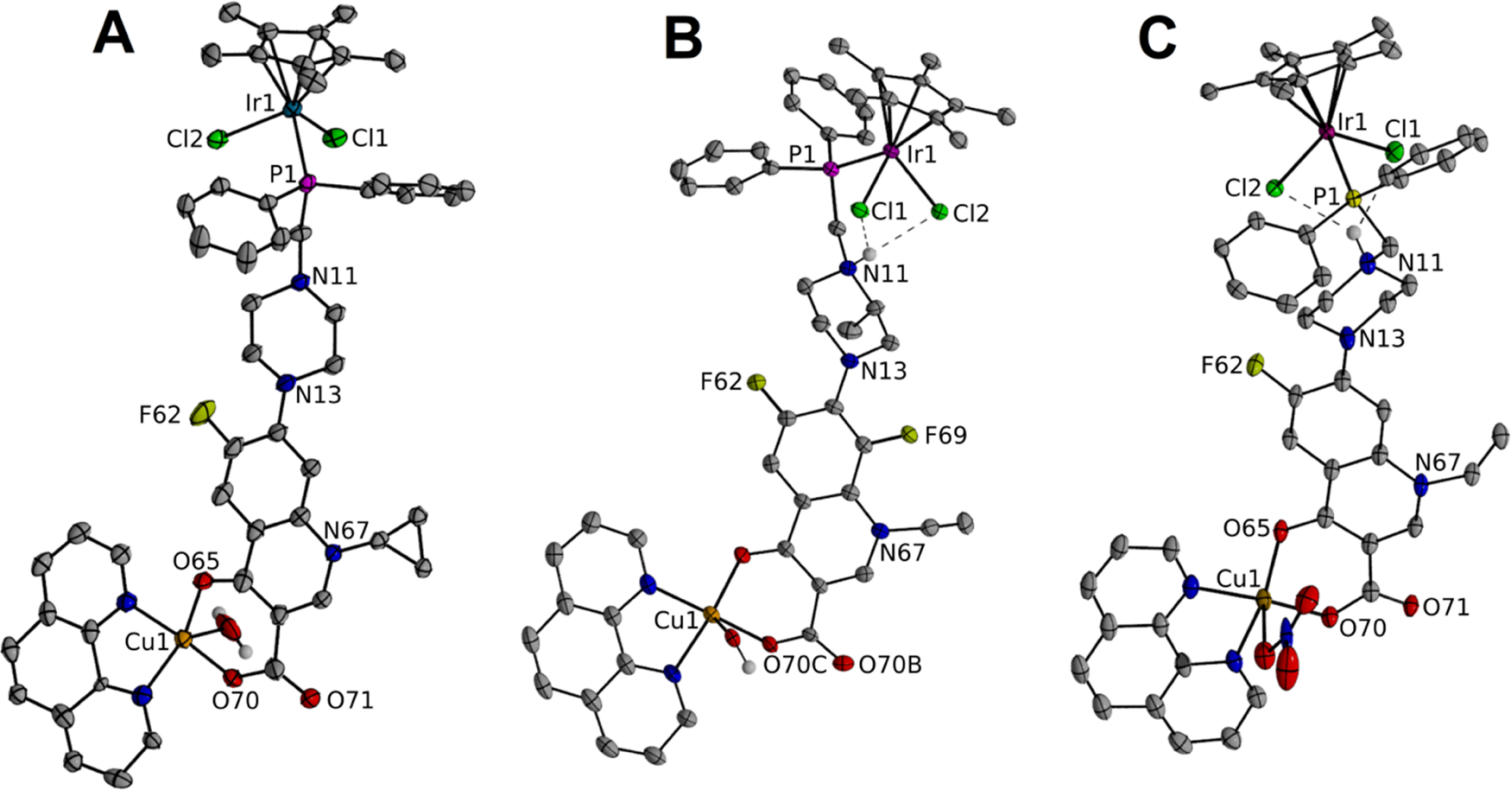
Crystal structures of the complex molecules IrPcfxCu (**1**), IrPnfxCu (**2**), and IrPlfxCu (**3**). The solvent molecules and protons are omitted for clarity.

The single crystals of [Ir(η^5^-Cp*)Cl_2_Pcfx-Cu(phen)](NO_3_)·1.75(CH_3_OH)·0.75(H_2_O) (**1**), [Ir(η^5^-Cp*)Cl_2_Pnfx-Cu(phen)](NO_3_)·1.75(CH_3_OH)·0.75(H_2_O) (**2**), and [Ir(η^5^-Cp*)Cl_2_Plfx-Cu(phen)](NO_3_)·1.3(H_2_O)·1.95(CH_3_OH) (**3**) were analyzed by the X-ray diffraction technique (Figures 1 and S1–S6; Tables 1, 2, and S1).

**Table 1:** Selected Bond Lengths (Å) for Crystallized Complexes

	IrPnfxrCu·(NO_3_)·1.75(CH_4_O)·0.75(H_2_O)	IrPcfxCu·(NO_3_)·2.75(H_2_O)	IrPlfxCu·(NO_3_)·1.3(H_2_O)·1.95(CH_4_O)
C(Cp*ring)–C(Cp*CH_3_)			1.4862
Ir^1^–C^6^	2.235(6)	2.245(6)	2.19(4)
Ir^1^–C^7^	2.224(6)	2.237(6)	2.21(3)
Ir^1^–C^8^	2.198(7)	2.142(6)	2.18(2)
Ir^1^–C^9^	2.195(6)	2.155(6)	2.19(2)
Ir^1^–C^10^	2.203(6)	2.154(6)	2.21(2)
Ir^1^–C_Cp*_ (average)	2.211(0)	2.186(6)	2.196
Ir^1^–C_centroid_			1.835
Ir^1^–P^1^	2.312(16)	2.291(15)	2.304(18)
Ir^1^–Cl^1^	2.410(2)	2.411(15)	2.407(18)
Ir^1^–Cl^2^	2.427(4)	2.448(17)	2.413(18)
P^1^–C^11^	1.839(5)	1.846(6)	1.859(7)
P^1^–C^21^	1.815(5)	1.822(6)	1.814(7)
P^1^–C^31^	1.812(5)	1.816(6)	1.819(7)
Cu^1^–O^70A^	1.911(4)	1.917(17)	2.057(12)
Cu^1^–O^70A′^			2.220(14)
Cu^1^–O^65A^	1.917(4)	1.919(17)	1.923(19)
Cu^1^–O^65B^			1.959(18)
Cu^1^–N^91^	2.019(5)	1.999(13)	2.004(7)
Cu^1^–N^81^	2.014(5)	2.013(12)	1.991(8)
Cu^1^–O^1N^	2.351(6)		
Cu^1^–O^1W^		2.32(2)	2.298(3)
FQ_centroid_–phen_Centroid_			3.743
Centroid—Center of Gravity			
Ir^1^–CCp* (average): average calculated from Ir^1^–C^1^, Ir^1^–C^2^, Ir^1^–C^3^, Ir^1^–C^4^, and Ir^1^–C^5^			
C(Cp*ring)–C(Cp*CH_3_): average calculated from C^1^–C^1A^, C^2^–C^2A^, C^3^–C^3A^, C^4^–C^4A^, and C^5^–C^5A^			

**Table 2:** Selected Angles (deg) for Crystallized Complexes

	IrPnfxCu·(NO_3_)·1.75(CH_4_O)·0.75(H_2_O)	IrPcfxCu·(NO_3_)·2.75(H_2_O)	IrPlfxCu·(NO_3_)·1.3(H_2_O)·1.95(CH_4_O)
P^1^–Ir^1^–Cl^1^	87.57(1)	86.73(5)	87.93(6)
P^1^–Ir^1^–Cl^2^	124.2(2)	91.14(6)	90.19
Cl^1^–Ir^1^–Cl^2^	86.96(13)	88.39(5)	88.28
C_centroid_–Ir^1^–P^1^			134.59
C_centroid_–Ir^1^–P^1^			121.28
C_centroid_–Ir^1^–P^1^			121.68
Ir^1^–P^1^–C^11^	114.44(18)	112.06(19)	114.0(2)
Ir^1^–P^1^–C^21^	111.4(2)	113.2(2)	118.6(3)
Ir^1^–P^1^–C^31^	118.76(19)	117.1(2)	116.0(2)
C^31^–P^1^–C^21^	105.5(2)	107.1(3)	103.8(3)
C^31^–P^1^–C^11^	104.4(3)	101.2(3)	97.2(3)
C^21^–P^1^–C^11^	100.4(2)	104.8(3)	104.4(3)
N^91^–Cu^1^–N^81^	82.1(2)	81.6(5)	82.1(3)
O^70A^–Cu^1^–O^65A^	94.52(16)	93.7(7)	88.2(6)
O^70A^–Cu^1^–O^65B^			95.9(6)
O^70A^–Cu^1^–O^70′^			71.69
O^65A^–Cu^1^–N^81^	90.9(2)	88.9(6)	91.0(6)
O^65B^–Cu^1^–N^81^			91.1(6)
O^70A^–Cu^1^–N^81^	172.94(19)	174.5(6)	157.1(5)
O^70′^–Cu^1^–N^81^			108.2(3)
O^65A^–Cu^1^–N^91^	162.68(19)	158.8(7)	167.9(6)
O^65B^–Cu^1^–N^91^			167.5(6)
O^70A^–Cu^1^–N^91^	91.43(18)	94.4(6)	94.4(4)
O^70A′^–Cu^1^–N^91^			86.2(4)
O^1N^–Cu^1^–N^91^	81.7(2)		
O^1N^–Cu^1^–N^81^	86.88(19)		
O^1N^–Cu^1^–O^65A^	113.91(18)		
O^1N^–Cu^1^–O^70A^	95.02(18)		
O^1W^–Cu^1^–N^91^		102.9(8)	88.6(3)
O^1W^–Cu^1^–N^81^		93.4(10)	108.2(3)
O^1W^–Cu^1^–O^65A^		96.4(9)	103.1(5)
O^1W^–Cu^1^–O^65B^			83.6(5)
O^1W^–Cu^1^–O^70A^			94.3(4)
O^1W^–Cu^1^–O^70A′^			164.5(4)
Centroid—Center of Gravity			
Ir^1^–CCp* (average): average calculated from Ir^1^–C^1^, Ir^1^–C^2^, Ir^1^–C^3^, Ir^1^–C^4^, and Ir^1^–C^5^			
C(Cp*ring)–C(Cp*CH_3_): average calculated from C^1^–C^1A^, C^2^–C^2A^, C^3^–C^3A^, C^4^–C^4A^, and C^5^–C^5A^			

All obtained iridium(III)–copper(II) complexes (Figure 1) crystallized in two different space groups (**1** and **2**: crystal system, triclinic; space group, *P*1; **3**: crystal system, orthorhombic; space group, *Pbcn*). Importantly, the crystal structure of **3** features the one-dimensional (1D) metal–organic polymer assembled from Cu(II) centers, Ir(III) complex linkers, 1,10-phenanthroline (phen) molecule, and OH^–^ ligands (Figure S1 see the Supporting Information). Every structure also contains solvent molecules and anions (H_2_O, NO_3_^–^, or OH^–^). The coordination geometry of the iridium ion in all of the Ir(III)–Cu(II) complexes adopts the well-known half-sandwich pseudo-octahedral “three-leg piano-stool” geometry, where the cyclopentadienyl moiety has served as the top of the stool and the three leg sites have been occupied by phosphorous atom from phosphine ligand and two terminal chloride anions.^27−30^

The bond angle values of P–Ir–Cl and Cl–Ir–Cl, proving the pseudo-octahedral arrangement of atoms around the metal center, are found to be in the range of 86.73–124.2°. They turned out to be higher than the values obtained for mononuclear Ir(III) complexes with the same phosphine ligands previously described by us (the bond angle values for P–Ir–Cl and Cl–Ir–Cl are in the range of 86.04–90.45°).^27^ The bond distances between iridium metal centers and phosphorus atoms of **1**, **2**, and **3** complexes have been found to be on average 2.3 Å, whereas the Ir–Cl1 and Ir–Cl2 bond distances of complexes **1**, **2**, and **3** have been found to be on average 2.4 Å and are comparable to that previously described for mononuclear Ir(III) complexes.^27−29^

Cu^II^ ion in all Ir(III)–Cu(II) complexes is coordinated *via* nitrogen atoms (from phenanthroline ligand) and the IrP(FQ) (where FQ denotes fluoroquinolone) complex *via* deprotonated carboxylate and pyridone oxygen atoms forming a distorted square-pyramidal coordination geometry (Figures 1 and S1–S6). Average bond lengths for **1** and **2** complexes are as follows: Cu1–N91, 2.002; Cu1–N81, 2.013; Cu1–O65, 1.918; and Cu1–O70, 1.914 Å. Additionally, in the case of **1**, the bond length between Cu1 and O1W (from water molecule) is equal to 2.232(1) Å, and for **2**, the bond length in Cu1–N (from NO_3_^–^ ion) is equal to 2.351(1) Å. Interestingly, the analysis of packing in **3** reveals that the coordination geometry around Cu^II^ ions is a distorted octahedron (Figures 1 and S4). The Cu^II^ ion is coordinated by four oxygen atoms (two carboxylate, one pyridine oxygen, and one from the OH– group) and two nitrogen atoms from the phenanthroline ring, leading to the formation of **3** polymer. This unit with two bridging OW1 and O70 atoms is formed with a Cu1–Cu1 distance of 4.193(1) Å, similar to that described in the literature for Cu^II^ complexes with other quinolone antibiotics and aromatic diimines.^43−46^

Additionally, the conformation of the antibiotic fragment of complexes can be defined in terms of torsion angle, defining the orientation of the piperazine ring and fluoroquinolone moiety (**1**: C13–N13–C61–C62 −157.56°; **3**: C13–N13–C14–C15 −128.45°; **2**: C14–N13–C61–C62 −153.29°). Those values differ from the torsion angle of the piperazine ring and antibiotic motif for analogic monometallic Ir(III) complexes with the same phosphine ligands (Ir(η^5^-Cp*)Cl_2_Pcfx: C13–N13–C61–C62 −165.92°; Ir(η^5^-Cp*)Cl_2_Plfx: C13–N13–C61–C62 −117.48°; Ir(η^5^-Cp*)Cl_2_Pnfx: C14–N13–C61–C62 −165.97°).^27^ The value of the torsion angle for **3** is significantly lower than the values of the torsion angle of both complexes **1** and **2**. This phenomenon can be explained by forming a dimer by **3** where many various interactions exist.

### Characterization of Ir(III)–Cu(II) Inorganic Compounds

#### Infrared Spectroscopies

The Fourier transform infrared (FT-IR) spectra of the four novel iridium(III)–copper(II) complexes together with analysis and discussion have been provided in the Supporting Information.

#### Electrospray Ionization Mass Spectrometry (ESI-MS)

All inorganic compounds have also been investigated by high-resolution mass spectrometry. In almost every case, a molecular ion peak was present and corresponded to isotopic distribution for a protonated parent ion [M + H]^+^. Only complex **1** did not show the corresponding molecular ion peaks, but [IrPCpCu-2Cl-2H + CH_3_OH]^+^ ions were detected at *m*/*z* 1129.264 (Figures S7–S10). Less abundant peaks corresponding to [M – Cl]^+^ and [M – 2Cl + H]^+^ ions have also been analyzed, indicating that chloride ions can be easily displaced. Surprisingly, we can also observe adducts with solvent molecules, either H_2_O or CH_3_OH. A solvent molecule can occupy the coordination site vacated by chloride ions. Additionally, peaks corresponding to the loss of the phosphine ligands and the arene ring are observed, which indicates poor metal-to-ligand and metal-to-arene binding. As illustrated in Figures S7–S10 in the Supporting Information, the cluster peaks obtained from the experiments exhibited excellent superimposition compared with those from simulations.

#### EPR Spectroscopy

The polycrystalline EPR spectra of the synthesized inorganic compounds have been recorded at room temperature and liquid nitrogen temperature (Figures S12 and S13, see the Supporting Information). No changes in the line shape, line width, and resolution as a function of temperature for all complexes have been observed. The anisotropic EPR spectral features can be linked with the axial symmetry having a d_*x*^2^–*y*^2^_ ground state, where the geometry can be assigned to an elongated octahedral, a square-pyramidal, or a square planar geometry. At the X-band, the EPR spectra for **1**, **2**, and **4** showed an asymmetry in the perpendicular region, with *g_x_* = 2.091, *g_y_* = 2.092, and *g_z_* = 2.21 (*g*_av_ = 2.13) for **1**, *g_x_* = 2.075, *g_y_* = 2.151, and *g_z_* = 2.11 (*g*_av_ = 2.11) for **2**, and *g_x_* = 2.091, *g_y_* = 2.132, and *g_z_* = 2.212 (*g*_av_ = 2.14) for **4** (see Figure S12, Supporting Information). This range of values is usually observed in the case of compounds with a distorted square-pyramidal geometry. The EPR spectra for **3** are typical of Cu(II) ion coordinated in a distorted octahedron with *g_x_* = 2.091, *g_y_* = 2.131, and *g_z_* = 2.291 (*g*_av_ = 2.17) for **3** in agreement with its structure.

The frozen solution EPR spectra of **1** and **2** compounds at 77 K show a well-defined resolution of hyperfine splitting of parallel orientation that is the result of the interaction of an unpaired electron with copper nuclei (*I* = 3/2). The spin Hamiltonian parameters have been calculated by computer simulation (sim Figure S13, see the Supporting Information) of the experimental spectra with *g_x_* = *g_y_* = *g*_⊥_ = 2.065, *g_z_* = *g_k_* = 2.211, and *A_k_* = 164 G for **1**, *g_x_* = 2.069, *g_y_* = 2.073, *g_z_* = *g_k_* = 2.215, and *A_k_* = 113 G for **2**, *g_x_* = *g_y_* = *g*_⊥_ = 2.098, *g_z_* = *g_k_* = 2.289, and *A_k_* = 163 G for **3**, and *g_x_* = *g_y_* = *g*_⊥_ = 2.068, *g_z_* = *g_k_* = 2.301, and *A_k_* = 146 G for **4**. Hence, the EPR parameters of all inorganic compounds in frozen solutions are also in agreement with the axial symmetry of the Cu(II) coordination sphere. Furthermore, the changes in parameter values upon dissolving complexes **1**, **2**, and **4** strongly suggest the replacement of the labile water or NO_3_^–^ ligands with the solvent molecules as has been found in many other already described complexes.^47,48^

#### Luminescence Properties

**Figure 2:**
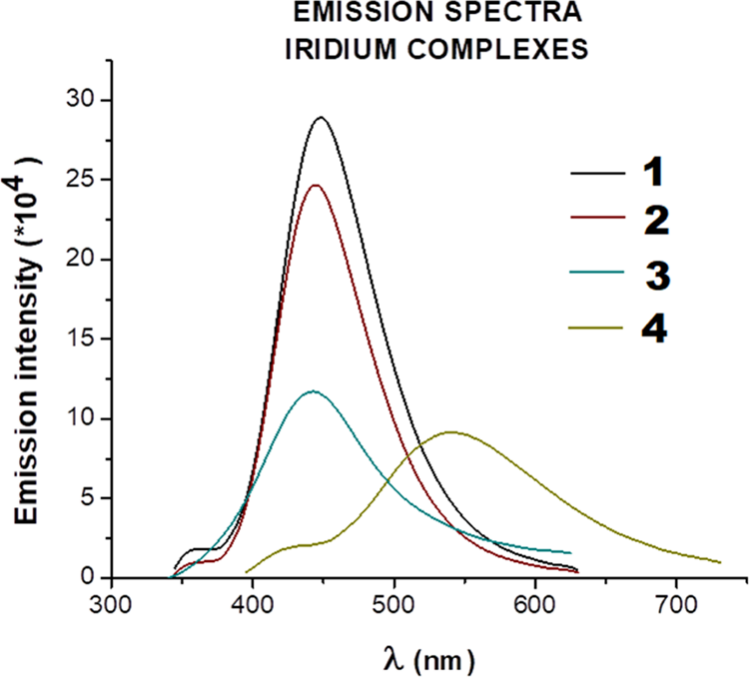
Emission spectra obtained for heteronuclear Ir^III^/Cu^II^ complexes in DMF, *C* ∼ 0.05 M.

All heteronuclear Ir^III^/Cu^II^ complexes emit in fluid solution at room temperature upon photoexcitation in the absorption manifold at 340 nm. The emission was structureless and peaked at 450 nm. A particular case is constituted by complex **4** that manifests an emission peak cantered at 540 nm (Figure 2). Moreover, the emission intensity was stronger in dimethylformamide (DMF) compared to DMSO, probably due to stronger solvation interactions in the case of DMSO, which enhances the nonradiative deactivation of these species (see Figure S14, Supporting Information). So, for this reason we decided to conduct all of the spectroscopic measures in DMF. The spectroscopic energy was around 3.2 eV for **1**, **2**, and **3** complexes and 2.85 eV for **4**, indicating a negligible influence of the solvent in tuning the excited state energy.

The coordination of Cu(II) to Ir(III) complexes, which was described by us previously, such as Ir(η^5^-Cp*)Cl_2_Pcfx, Ir(η^5^-Cp*)Cl_2_Plfx, and Ir(η^5^-Cp*)Cl_2_Pnfx, leads to the bathochromic shift of the peak emission maximum wavelength. However, the incorporation of another metallic center to Ir(η^5^-Cp*)Cl_2_Psfx causes the red shift of **4** in comparison to Ph_2_PCH_2_sfx but a blue shift toward the Ir(η^5^-Cp*)Cl_2_Psfx. Therefore, **1** (λ_max_ = 448 nm), **2** (λ_max_ = 442 nm), and **3** (λ_max_ = 470 nm) exhibited purple emission, whereas **4** (λ_max_ = 513 nm) exhibited green emission (see Figures S15 and S16, Supporting Information).

It could be concluded that the emission wavelength associated with these bimetallic Ir–Cu complexes is strongly related to the nature of the chosen ligand. While **1**, **2**, and **3** seem to exhibit the same spectroscopic trend, only **4** seems to manifest a different emission pattern.^27^ The outcome of conducted luminescence experiments corresponds to our previous findings, not only for Ir(III) complexes but also for Ru(II) as well as Cu(I) with the same phosphine ligands.^13,26,27^ Luminescence properties of studied complexes are helpful in detecting their intercellular localization. We were able to localize the complexes inside cancer cells, and the results obtained by us are presented below.

#### Magnetic Properties

**Figure 3:**
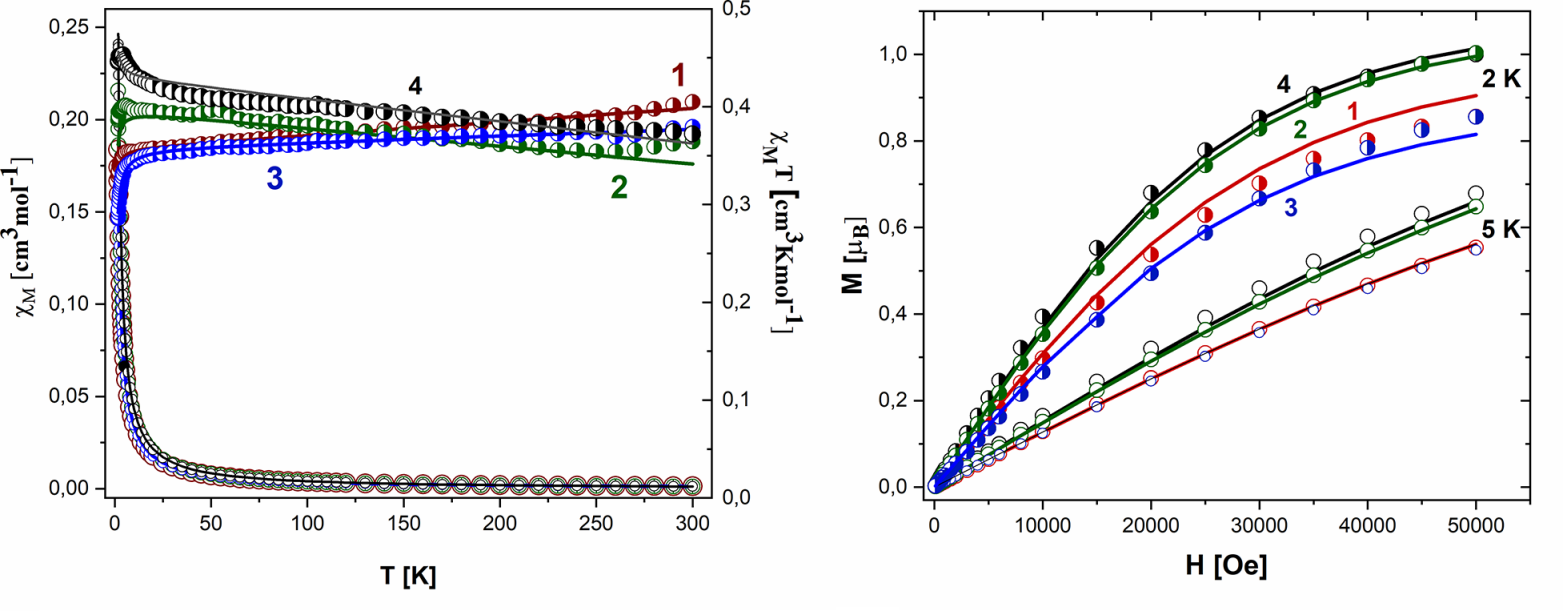
Left: thermal dependence of χ_M_*T* (half-open circles) and χ_M_ (open circles) for **1**, [Ir(η^5^-Cp*)Cl_2_Pcfx-Cu(phen)]; **2**, [Ir(η^5^-Cp*)Cl_2_Pnfx-Cu(phen)]; **3**, [Ir(η^5^-Cp*)Cl_2_Plfx-Cu(phen)]; and **4**, [Ir(η^5^-Cp*)Cl_2_Psfx-Cu(phen)]. Right: magnetization as a function of the magnetic field at 2.00 K (half-open circles) and 5.00 K (open circles) for **1**, IrPcfxCu; **2**, IrPnfxCu; **3**, IrPlfxCu; and **4**, IrPsfxCu. The solid lines (on all graphs) are calculated using the Heisenberg–Dirac–van Vleck (HDVV) spin Hamiltonian and PHI.^49^

Magnetic data were acquired with the help of the SQUID magnetometer (MPMS, Quantum Design) at an applied field of *B*_0_ = 0.5 T, after correction to the underlying diamagnetism, and transformed to the temperature dependence of the χ_M_*T* product (or effective magnetic moment) (Figure 3, left). The field dependence of the magnetization per formula unit *M*_1_ = *M*_mol_/*N*_A_ μ_B_ at a constant temperature is shown in Figure 3, right.

All examined complexes magnetically behave as a mononuclear unit with *S*_Cu_ = 1/2 because Ir^III^ ions are diamagnetic. Assuming *g* = 2.0, the expected high-temperature value for the *S* = 1/2 spin system is μ_eff_ = *g*[*S*(*S* + 1)]^1/2^ = 1.73 μ_B_. The experimental data for **1**–**4** compounds show a value of μ_eff_ = 1.80 μ_B_ for **1** (χ_M_*T* = 0.41 cm^3^ K/mol), μ_eff_ = 1.75 μ_B_ for **2** (χ_M_*T* = 0.39 cm^3^ K/mol), μ_eff_ = 1.74 μ_B_ for **3** (χ_M_*T* = 0.38 cm^3^ K/mol), and μ_eff_ = 1.73 μ_B_ for **4** (χ_M_*T* = 0.38 cm^3^ K/mol) at *T* = 300 K. The χ_M_*T* (and/or the effective magnetic moment) (Figure 3 left) for **1** and **3** decreases slowly with decreasing the temperature down to *T* = 25 K. Below 50 K, a rapid decrease of the χ_M_*T* was observed, reaching an χ_M_*T* value of 0.33 cm^3^ K/mol (1.62 μ_B_) for **1** and an χ_M_*T* of 0.29 cm^3^ K/mol (1.51 μ_B_) for **3** at 1.8 K. This feature indicates the antiferromagnetic nature of intramolecular exchange interactions mediated through carboxylate groups of phosphino-fluoroquinolone ligands and OH– linkers in **3** or intermolecular interaction between the nearest copper ion in the supramolecular architecture. Compounds **2** and **4** exhibit weak intermolecular ferromagnetic coupling (systematic increase of χ_M_*T* value with the temperature) with similar strength (Figure 3, left), which can be seen from almost the same position of the maximum of χ*T*. The magnetization data at *T* = 2.0 and *B*_DC_ = 5.0 T saturates to *M*_1_ = *M*_mol_/(*N*_A_μ_B_) = 0.86 μ_B_ (**1**, **3**) and 1.00 (**2**, **4**), which confirms the presence of some weak antiferromagnetic interactions in the case of **1** and **3**. A significant contribution of the temperature-independent paramagnetism (TIP) is reflected in the slopes of the χ_M_*T* dependencies at higher temperatures. A more detailed discussion about the pathways of magnetic exchange interactions together with theoretical analysis based on proper Hamiltonian is presented in the Supporting Information.

Additionally, unexpected information was obtained from the AC susceptibility measurements for complex **2**. The in-phase (χ*M*′) and out-of-phase (χ*M*″) components exhibit small frequency dependence with the application of an external field of 0.2 T, indicating the possibility of a slow relaxation of magnetization typical for SMM or SIM behavior (Figures S17 and S18, see the Supporting Information). This phenomenon for Cu(II) ions is very rare due to the absence of a barrier to spin reversal: the axial zero-field splitting parameter *D* is undefined. More details on the AC measurement procedure and an explanation of the presence of the relaxation process are provided in the Supporting Information.

#### Electrochemical Behavior

We decided to investigate the electrochemical properties of the Ir(III)–Cu(II) complex to understand its role in cellular signaling through redox chemistry. Any disruptions of intracellular redox processes can significantly affect a plethora of cellular processes such as proliferation. This may, in turn, result in serious consequences (*e*.*g*., cell death).^4^

**Figure 4:**
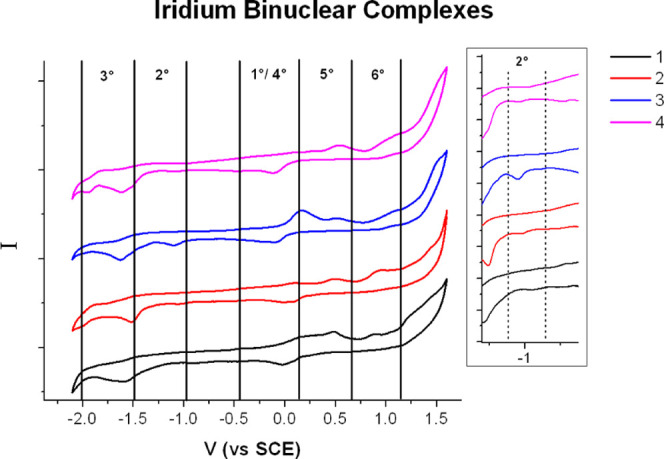
Cyclic voltammetry of binuclear complexes of Ir^III^/Cu^II^ in DMF (50 mV/s from −2.1 to +1.6 *vs* SCE, TBAPF_6_ 0.1 M as a supporting electrolyte).

Cyclic voltammetry of iridium binuclear complexes at 50 mV/s in purged DMF is reported in Figure 4. The scans were recorded by scanning first in the cathodic direction (toward negative voltage) and then moving back to positive values. At a negative voltage, we observed two major irreversible reductive waves, the first at *ca*. −1.1 V *vs* saturated calomel electrode (SCE) for all heteronuclear complexes Ir^III^/Cu^II^ (wave 2°), followed by another more intense reduction wave centered at *ca*. −1.6 V *vs* SCE (wave 3°) that, upon back scanning, results in a first anodic feature at +0.2 V (wave no. 4), followed by a convolved response spanning the voltage range +0.4, +1.3 V (wave nos. 5, 6) (see Figure S19in the Supporting Information). In contrast, the restriction of the voltage scan to −0.3/+0.3 V *vs* SCE only makes evident the clean quasireversible process of Cu(II)/(I) with *E*_1/2_ of *ca*. 0 V (peaks **1** and **4**). The complex appearance of the voltammetric waves in the 0/+1.3 V range of the full scan mostly originates from the oxidation of decomposition products generated during the cathodic scan at potentials more negative than −0.9 V *vs* SCE.

#### Biological Study

The cytotoxic potency of a drug is generally associated to its cellular uptake, which is in turn connected to its lipophilicity. In general, hydrophobicity plays a critical role in structure–activity correlations and in determining the biological properties of the drug.^27^ The octanol–water partition coefficients (log *P*) for all complexes were determined and are listed in Table S3 in the Supporting Information (log *P* values have been calculated using programs ACD/log *P* and ALOGPS 2.150). The complexes containing Cu(phen) ligands show substantially lower lipophilicity than mononuclear complexes, which is in accordance with the hydrophilic nature of the diimine ligands.^50^

The cytotoxicity of the four novel heteronuclear Ir^III^–Cu^II^ complexes was assessed against four selected cancer cell lines: A549, MCF7, DU145, WM2664, and one normal HEK293T cell line *in vitro* (Table 3). IC_50_ values (concentration of a drug required to inhibit the growth of 50% of the cells) were assessed in two different approaches, *i.e*., after 24 or 24 + 48 h, using the 3-(4,5-dimethylthiazole)-2,5-diphenyltetrazolium bromide (MTT) method. Cancerous and noncancerous diseases have also been treated with the well-known drug cisplatin in the same concentration range as investigated complexes and are considered as a control. IC_50_ values have been determined from the plots of cell viability at various concentrations of each compound by matching appropriate dose–response curves and are presented in Table 3.

**Table 3:** Values of IC_50_ [μM] (Concentration of a Drug Required to Inhibit the Growth of 50% of the Cells) for WM2661, A549, MCF7, DU145, and HEK293T Cells after 24 and 24 + 48 h Treatment with the Studied Compounds and Cisplatin as Reference

IC_50_ [μM] ± SD; 24 h					
	A549	MCF7	DU145	WM2664	HEK293T
IrPcfxCu (**1**)	35.5 ± 5.6 × 10^–3^	35.3 ± 6.5	12.8 ± 2.7 × 10^–7^	5.2 ± 4.1 × 10^–3^	786.8 ± 11.2
IrPnfxCu (**2**)	11.2 ± 7.8 × 10^–3^	30.0 ± 0.7	10.8 ± 1.9× 10^–4^	9.9 ± 3.8 × 10^–3^	756.8 ± 5.7
IrPlfxCu (**3**)	31.6 ± 7.6 × 10^–3^	24.2 ± 7.2	14.2 ± 2.4 × 10^–3^	10.1 ± 2.2 × 10^–3^	775.8 ± 15.7
IrPsfxCu (**4**)	18.1 ± 1.3 × 10^–3^	10.5 ± 0.8	10.1 ± 2.9 × 10^–3^	6.2 ± 2.4 × 10^–3^	676.8 ± 9.2
cisplatin (CDDP)	57.0 ± 1.3 × 10^–3^	51.9 ± 4.6	68.3 ± 1.3 × 10^–3^	2.6 ± 0.6	21.0 ± 1.8
Cu(phen)(NO_3_)_2_	536.8 ± 13.2	317.8 ± 12.2	412.4 ± 10.9	933.1 ± 13.6	81.8 ± 8.2

IC_50_ [μM] ± SD; 72 h (24 + 48 h)					
	A549	MCF7	DU145	WM2664	HEK293T
IrPcfxCu (**1**)	42.4 ± 7.3 × 10^–5^	>1000	125.7 ± 3.4	137.1 ± 2.2	886.8 ± 12.7
IrPnfxCu (**2**)	36.6 ± 2.8 × 10^–3^	>1000	122.7 ± 5.4	155.1 ± 3.2	856.8 ± 15.9
IrPlfxCu (**3**)	36.0 ± 2.2 × 10^–2^	>1000	126.2 ± 4.4	229.3 ± 25.9	822.8 ± 12.3
IrPsfxCu (**4**)	0.6 ± 2.9 × 10^–4^	>1000	53.6 ± 0.40	212.2 ± 10.5	776.8 ± 15.3
cisplatin (CDDP)	71.7 ± 3.7	17.7 ± 8.6	65.5 ± 3.6	8.3 ± 0.4	10.3 ± 2.1
Cu(phen)(NO_3_)_2_	721.2 ± 15.1	425.5 ± 9.9	658.9 ± 14.5	1106.5 ± 18.5	42.4 ± 9.1

Notably, all of the heteronuclear Ir^III^/Cu^II^ complexes showed higher cytotoxicity (after 24 h of incubation) than cisplatin against all studied cell lines except the WM2664 cells. Interestingly, lung carcinoma cancer cells (A549) were the most sensitive cell line to heteronuclear Ir^III^–Cu^II^ complexes even in the case of both incubation times (24 and 24 + 48 h). Among all investigated inorganic compounds, complex **4** exhibited the most significant antiproliferative activity *in vitro* with an IC_50_ value of 0.6 μM against the A549 cell line, which was more than 100 times more effective than the reference drug cisplatin. Cytotoxic activity of complex **4** toward A549 cells was higher after 24 and 48 h of recovery time (24 + 48 h), compared to the experimental approach where cytotoxicity was evaluated after 24 h. This indicates that intercellular biochemical changes initiated during the 24 h of incubation cannot be repaired by the cells. Repair systems responsible for minimizing toxicity are almost not sufficient and may result in breaking down the resistance. In addition, compound **4** elicited a moderate cytotoxic effect against other tumor cell lines (except MCF7 after 72 h). Therefore, this suggests that the introduction of the third-generation fluoroquinolone (Ph_2_PCH_2_sfx) causes a significant increase in cytotoxicity to A549 cells compared to the complexes containing the second-generation fluoroquinolones (Ph_2_PCH_2_nfx, Ph_2_PCH_2_cfx, Ph_2_PCH_2_lfx). This is consistent with our previous observations about the cytotoxicity of mononuclear Ir^III^ complexes with the same phosphine ligands.^27^

The described results show that the activity of all complexes against WM2664 is in the same range as for cisplatin after 24 h. Such good cytotoxicity *in vitro* was achieved simultaneously with very low toxicity toward normal cell lines. A comparison of the IC_50_ values shown by heteronuclear ([Ir(η^5^-Cp*)Cl_2_Psfx-Cu(phen)] (**4**), [Ir(η^5^-Cp*)Cl_2_Plfx-Cu(phen)] (**3**), [Ir(η^5^-Cp*)Cl_2_Pcfx-Cu(phen)] (**1**), [Ir(η^5^-Cp*)Cl_2_Pnfx-Cu(phen)] (**2**)) *vs* mononuclear complexes ([Ir(η^5^-Cp*)Cl_2_Psfx], [Ir(η^5^-Cp*)Cl_2_Plfx], [Ir(η^5^-Cp*)Cl_2_Pcfx], [Ir(η^5^-Cp*)Cl_2_Pnfx]) indicated that the presence of second metal caused a significant decrease in the toxicity against the HEK293T cell line.^27^ In the A549 cell line, the selectivity index (SI – IC_50_ (normal cell line)/IC_50_ (cancer cell line)) was found to be approximately 1290 for complex **4**. Remarkably, such a high value was achieved despite the addition of a second metal with phenanthroline, which is known to be toxic.^51^ This suggests that the introduction of a second metal is an effective method of minimizing toxicity to healthy cells and may bring into play different properties of the resulting compound.

#### Characterization of Liposomes

**Figure 5:**
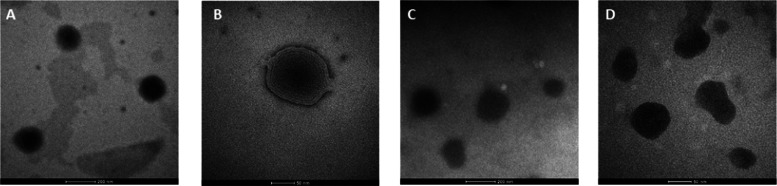
TEM images of empty liposomes (A, B) and liposomes loaded with the Ir^III^/Cu^II^ complex (**1a**; C, D).

To overcome toxicity and low water solubility, complex [Ir(η^5^-Cp*)Cl_2_Pcfx-Cu(phen)] (**1**) has been encapsulated inside liposomes (**1L**) in two different concentrations (**1La**: 0.25 mg/mL and **1Lb**: 0.5 mg/mL). Negative staining transmission electron microscopy (TEM) images exhibited a spherical shape with a smooth surface and laminar character of homogeneous liposomal dispersion (Figure 5).

Statistical analysis of liposome size obtained from TEM analysis (ImageJ) is in agreement with dynamic light scattering (DLS) data (Table S4, see the Supporting Information). Although the size differences between TEM and DLS can be clarified by the technique differences, in TEM, the nanoparticles are dried and the size should be smaller after shrinking than in the case of DLS.^52^ The average size of empty liposomes was *ca.* 135 nm, while that of liposomes loaded with the Ir^III^/Cu^II^ complex was *ca*. 110 nm, with the polydispersity index indicating a quite narrow size distribution. The ζ-potential (the electrostatic repulsion of the particle surface) was examined in phosphate-buffered saline (PBS) (pH = 7.4), resulting in negative values for empty and loaded liposomes (*ca*. −30 and −42 mV, respectively) and indicating a stable dispersion.

The anticancer activity of the tested iridium liposomes was evaluated by their cytotoxicity, determined by the MTT assay against cancer (A549, DU145) and noncancer (HaCaT) cells. The IC_50_ values (concentration at which 50% of the cells are not able to grow any longer) for the tested compounds after 24 h are summarized in Table 4.

**Table 4:** IC_50_ (μg/mL and μM) Values of the Investigated Compounds toward the Selected Cancer (A549, DU145) and Noncancer (HaCaT) Cell Lines for 24 h^a^

	HaCaT	A549	Du145
**L**	180.47 ± 0.108 μg/mL	21.18 ± 0.143 μg/mL	12.56 ± 0.175 μg/mL
**1La**	260.92 ± 0.172 μg/mL	12.31 ± 0.130 μg/mL	1.74 ± 0.132 μg/mL
	200.68 ± 2.081 μM	9.47 ± 0.261 μM	1.34 ± 0.051 μM
**1Lb**	200.20 ± 0.118 μg/mL	7.26 ± 0.124 μg/mL	1.56 ± 0.128 μg/mL
	154.14 ± 1.051 μM	5.58 ± 0.121 μM	1.20 ± 0.071 μM
CDPP	9.69 ± 0.206 μg/mL	17.16 ± 0.181 μg/mL	20.56 ± 0.219 μg/mL
	32.20 ± 0.881 μM	56.99 ± 0.481 μM	68.28 ± 2.081 μM

a **1La**: 0.25 mg/mL and **1Lb**: 0.5 mg/mL.

The cytotoxic activity of the used carriers (liposome, **L**), the reference compound cisplatin (CDPP), and complex [Ir(η^5^-Cp*)Cl_2_Pcfx-Cu(phen)] (**1L**) encapsulated inside liposomes in two different concentrations (**1La**: 0.25 mg/mL and **1Lb**: 0.5 mg/mL) were also determined. It was observed that the empty liposome exhibited no cytotoxicity, while IC_50_ values for liposomes loaded with the Ir/Cu compounds (**1La** and **1Lb**) were lower than that for CDDP. In the case of the DU145 line, a 10-fold decrease was observed for complex accumulated inside the liposome. Moreover, the compounds exhibited relatively lower cytotoxic activity against normal cells, human keratinocytes.

#### First Insight into the Cytotoxic Action Mode

To investigate the mechanism underlying the activity of liposomes loaded with the Ir^III^/Cu^II^ complex in selected cell lines (HaCat, A549, and DU145) and to locate their possible targets in the cell, total cellular uptake and confocal microscopy tests were first performed. Cellular accumulation of **1** in liposomes has been determined by the inductively coupled plasma mass spectrometry (ICP-MS) technique.

**Figure 6:**
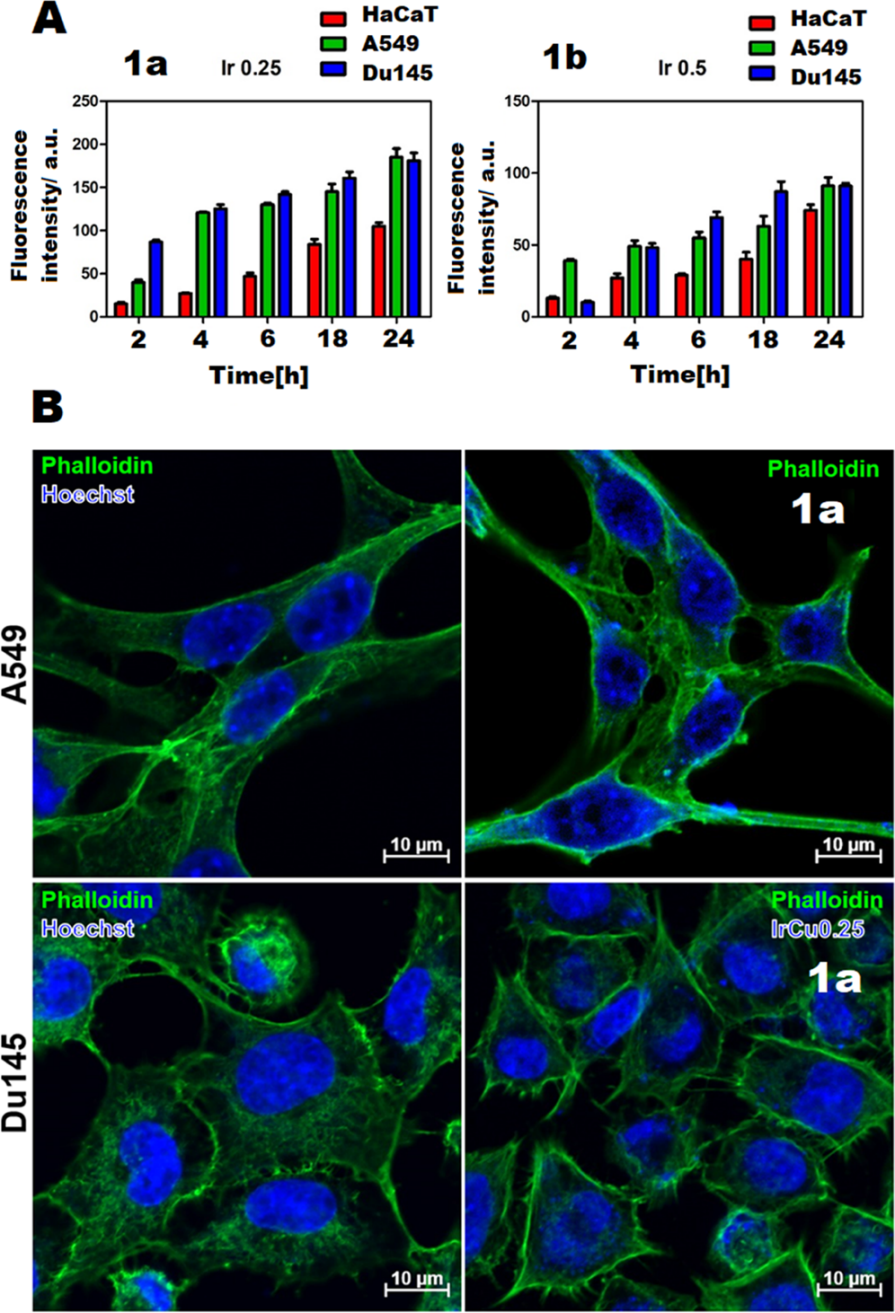
Cellular uptake. (A) Accumulation of compounds (**1La**, IrPcfxCu_L0.25; **1Lb**, IrPcfxCu_L0.5) in cells as a function of incubation time. (B) Accumulation of **1a** in A549 and DU145 cells. Green fluorescence, phalloidin; blue fluorescence, tested compounds.

The time-dependent cellular uptake of **1L** compounds is given in Figure 6A. The results obtained confirm that a higher accumulation of liposomes in cells is observed with the increase of time. The highest accumulation of the compound was observed after 24 h. The compound **1L** accumulated to a greater extent because a smaller amount of the compound was encapsulated in the liposomes, making them smaller in size. Moreover, we indicated the main accumulation of **1L** in nuclei (Figure 6B). It is noteworthy that the accumulation of **1L** differed significantly between cancer and normal cells, showing lower accumulation in the noncancer HaCaT cell line. These results fully corroborate the differences between the cytotoxicity of loaded nanoformulations against the cancer lines and the normal cell line.

**Figure 7:**
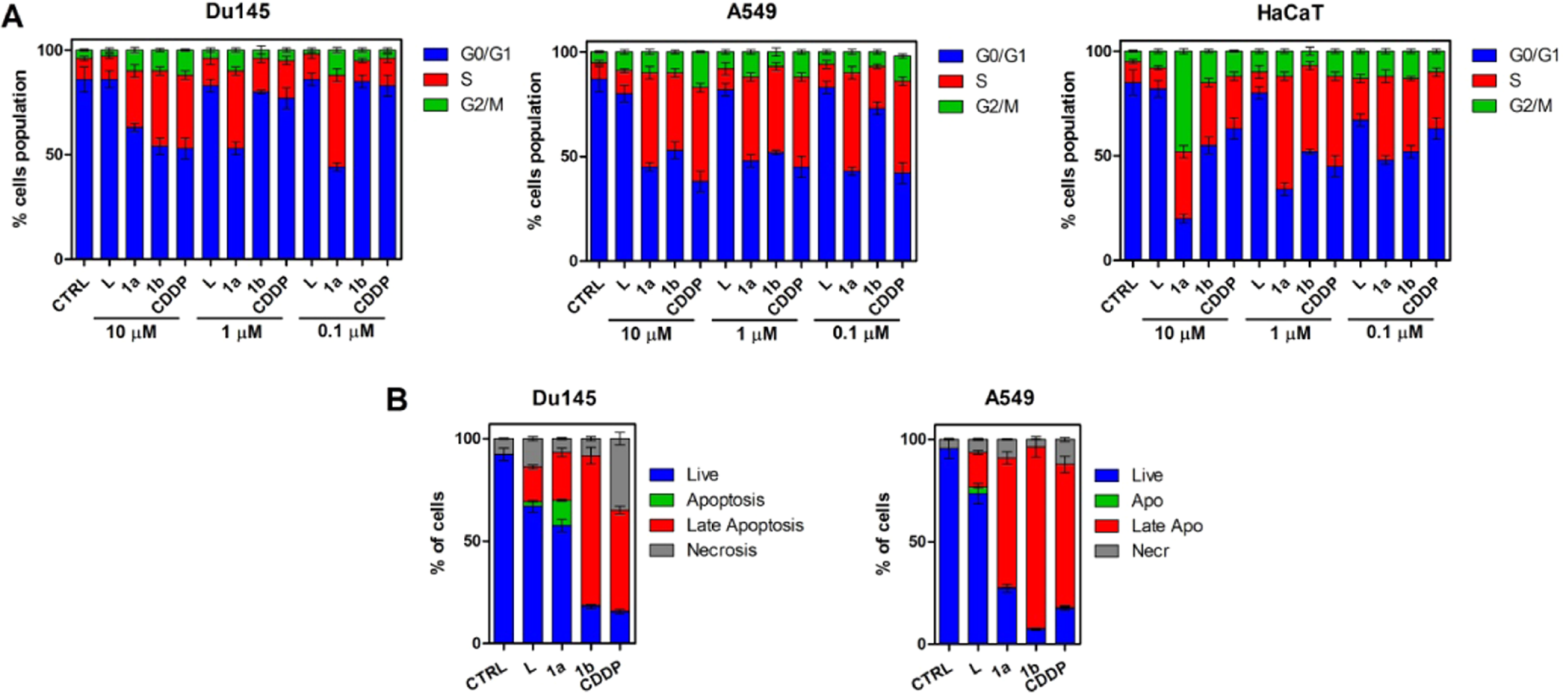
(A) Cell cycle analyses. HaCaT, A549, and DU145 cells were treated with the investigated compounds for 24 h and then stained with PI. (B) Cell death mechanisms. Annexin V–fluorescein isothiocyanate (FITC)/PI double staining of A549 and DU145 spheroids treated with the investigated compounds at concentrations equal to IC_50_ values (**1a**: 10 and 1 μM; **1b**: 5 and 1 μM; CDDP: 17 and 20 μM for A549 and DU145, respectively) for 24 h.

The activity of the tested compounds is certainly associated with cell cycle disruption (Figure 7). Previously published studies confirm that cell cycle arrest is a characteristic of many compounds with anticancer properties. The cell cycle is divided into three main phases: the G0/G1 phase, the S phase, and the G2/M phase.^53^ DNA content was measured using propidium iodide (PI) and analyzed by flow cytometry (Figure 7A). Before the experiment, the cells were synchronized and then the test compounds were added at different concentrations. Iridium liposomes strongly inhibit the ability of the cell to further divide regardless of the dose used. Compound **1L**, even at 1000-fold dilution, arrests the cell cycle in the S phase for the A549 line and shows effects similar to CDDP. In studies on the DU145 cell line, an approximately 3-fold increase in S-phase cells was obtained for this compound compared to the reference compound.

The cell cycle of normal cells (HaCaT) was not as strongly inhibited as for cancer cells. A similar effect could be observed for empty carriers (L). These results indicate that the tested compounds induce cell cycle disruption.

Induction of apoptotic death in response to anticancer drugs is one of the most important mechanisms for cancer cell death. Most tumor cells exhibit sensitivity to certain apoptotic stimuli from chemotherapeutic agents.^41^ The obtained results confirm that the cell death mechanisms are very similar for the iridium liposomes tested. Interestingly, compounds with lower iridium concentrations (**1La**) induce a high percentage of cells in late apoptosis (Figure 7B). In the case of the DU145 line, less toxicity of the compounds is observed, particularly for the liposomes with higher iridium concentrations (**1Lb**), for which the percentage of apoptotic cells is also the highest. Analyzing the cell death mechanisms induced by iridium liposomes, it can be observed that compounds with lower iridium concentration were more effective, and the percentage of living cells was 7% for the A549 cell line and 18% for the DU145 cell line, respectively. Compared to cisplatin, a significantly higher percentage of late-apoptotic cells than necrotic cells was observed after treatment with the tested compounds. Apoptosis is a more desirable cell death process because it does not lead to the inflammatory process that occurs with necrosis.

**Figure 8:**
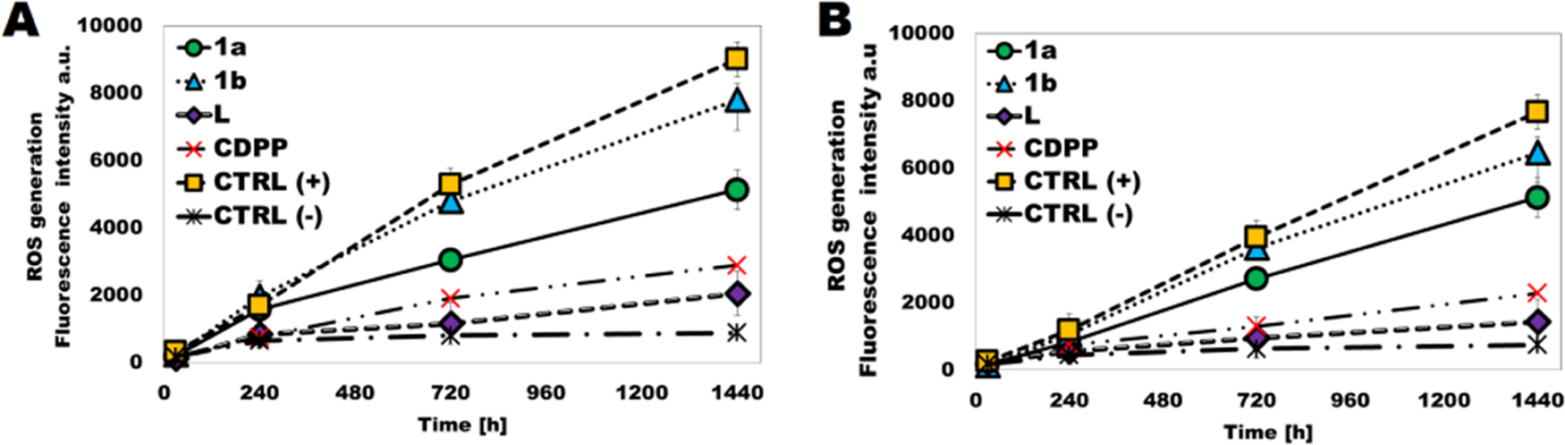
ROS production after 30 min and 4, 12, and 24 h using H_2_DCF-DA for **1a**, **1b**, **L**, CDPP; CTRL(+) (H_2_O_2_ as a positive control), and CTRL(−) (negative control); cells without compound in (A) A549 and (B) DU145. Data are expressed as mean ± scanning electron microscopy (SEM).

Several studies have demonstrated that cancerous damage by metal complexes leads to the overproduction of reactive oxygen species (ROS). An increased ROS level can cause a cell cycle arrest or lead to apoptotic cancer cell death.^13^ Therefore, it was important to check whether the cellular damage induced by the liposomal Ir(III)–Cu(II) complex (**1L**) would lead to increased ROS production. Cellular ROS generation in A549 and DU145 cells upon 30 min and 4, 12, and 24 h treatment with empty liposome (**L**), **1L**, and cisplatin (CDPP) was monitored by a fluorescent H_2_DCF-DA ROS probe (λ_ex_ = 495 nm, λ_em_ = 530 nm). H_2_O_2_ was used as a positive control in the first and second cases, respectively. The fluorescence intensity-ROS dependence over time is presented in Figure 8A,B. It was proved that the investigated complex **1L** encapsulated inside liposomes in two different concentrations (**1La**, **1b**) induced ROS production inside the treated DU145 and A549 cancer cells. It was proven that ROS generation in both cancerous lines treated with liposomal complex caused a significant increase over time in a concentration-dependent manner.

**Figure 9:**
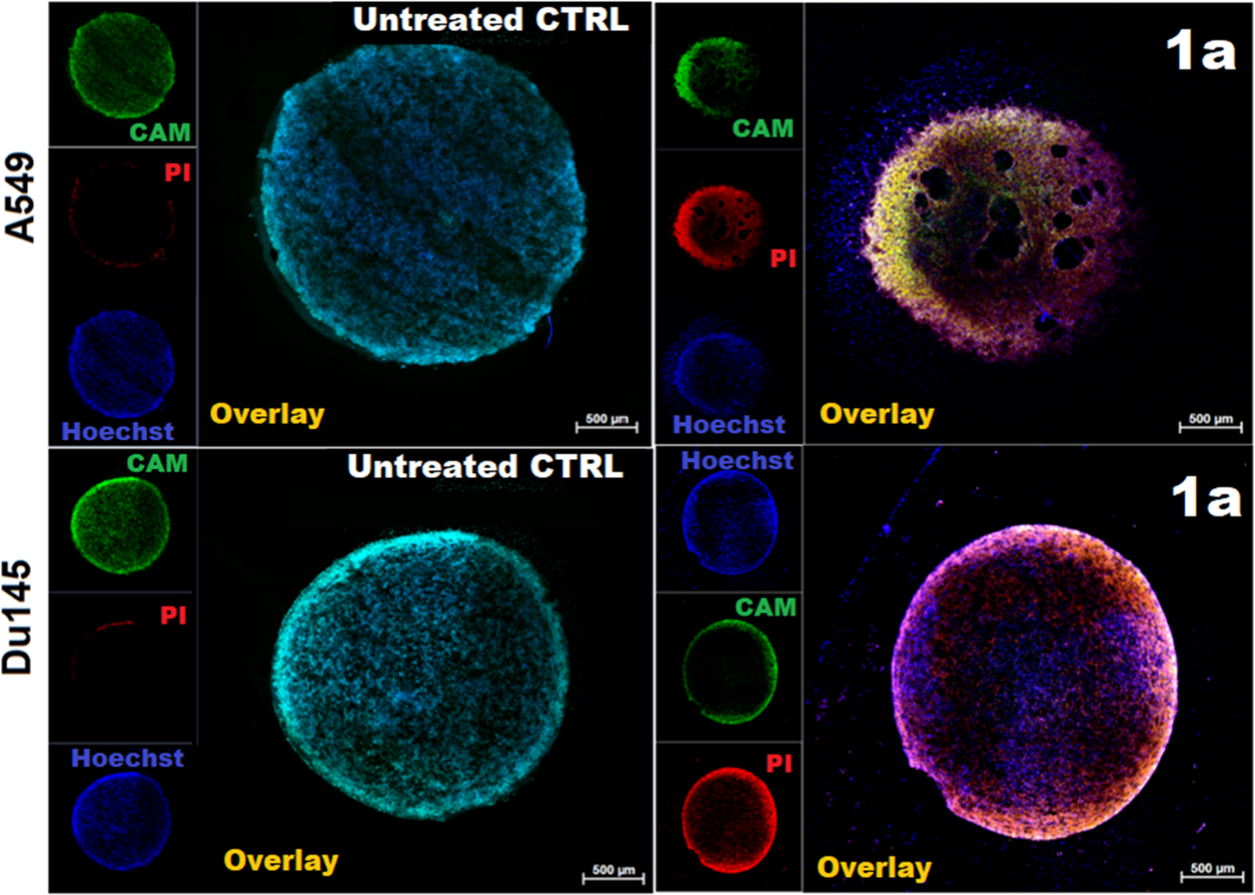
A549 (on top) and DU145 (on bottom) spheroids: representative live/death fluorescence images of spheroids subjected to **1a** and corresponding control untreated spheroids grown in the same culturing conditions.

The results obtained on two-dimensional (2D) cell culture encouraged us to investigate the antitumor activity of the selected Ir^III^–Cu^II^ complex (**1a**) also in 3D A549 and DU145 spheroidal culture. In addition, compared to classic adherent culture, spheroids can provide a microenvironment that more closely mimics the cellular interactions observed in tumor tissues. The therapeutic potential of synthesized compounds on 3D spheroids was detected by fluorescence staining of live and dead cells (Figure 9). For untreated 3D spheroid with normal morphology and structure, mostly we observed live cells and a small amount of dead cells mainly in the superficial regions. In contrast, after spheroid treatment by complex, we observed a significant increase in dead cells after spheroid treatment by complex.

## Conclusions

The present work demonstrates the synthesis, physicochemical characterization, and anticancer activity *in vitro* of [Ir(η^5^-Cp*)Cl_2_Pcfx-Cu(phen)] (IrPcfxCu (**1**)), [Ir(η^5^-Cp*)Cl_2_Pnfx-Cu(phen)] (IrPnfxCu (**2**)), [Ir(η^5^-Cp*)Cl_2_Plfx-Cu(phen)] (IrPlfxCu (**3**)), and [Ir(η^5^-Cp*)Cl_2_Psfx-Cu(phen)] (IrPsfxCu (**4**)) complexes. All inorganic compounds were characterized by ESI-MS spectrometry, selected spectroscopic methods (*i*.*e*., IR, fluorescence, and EPR), cyclic voltammetry, and variable-temperature magnetic susceptibility measurements. Three of four complexes were structurally identified by single-crystal X-ray diffraction analysis.

The coordination geometry of the iridium(III) ion in all Ir^III^–Cu^II^ complexes adopts the half-sandwich pseudo-octahedral “three-leg piano-stool” geometry. The cyclopentadienyl fragment served as the top of the stool and the three leg sites were occupied by phosphorous from phosphine ligand and two chloride anions. Cu^II^ ion in all Ir^III^–Cu^II^ complexes is coordinated *via* nitrogen atoms (from phenanthroline ligand) and IrP(FQ) complexes (where FQ denotes fluoroquinolone) *via* deprotonated carboxylate and pyridone oxygen atoms forming a distorted square-pyramidal coordination geometry in the case of IrPcfxCu (**1**) and IrPnfxCu (**3**) complexes. Importantly, the crystal structure of [Ir(η^5^-Cp*)Cl_2_Plfx-Cu(phen)] features the 1D metal–organic polymer and the analysis of packing in **3** reveals that the coordination geometry around Cu^II^ ion is distorted octahedral.

Furthermore, investigation of the elucidation of the behavior of the Ir^III^–Cu^II^ compounds allowed us to formulate the following general conclusions: (i) All complexes exhibit intense emission in solution; (ii) Redox activity of all complexes was confirmed by cyclic voltamperometry; (iii) All complexes are characterized by weak magnetic properties. Surprisingly, one of the studied systems [Ir(η^5^-Cp*)Cl_2_Pnfx-Cu(phen)] exhibits a slow magnetic relaxation under the moderate DC magnetic field, which is extremely rare in the case of Cu(II) complexes; (iv) The heteronuclear Ir^III^/Cu^II^ complexes displayed higher cytotoxicity than cisplatin against all cell lines (A549, MCF7, DU145) except for the WM2664 cell line. (v) After encapsulation of complex **1** in liposomal formulation, a 10-fold cytotoxicity decrease toward the DU145 cell line was observed. (vi) Liposomes loaded with [Ir(η^5^-Cp*)Cl_2_Pcfx-Cu(phen)] effectively accumulate inside human lung adenocarcinoma and human prostate carcinoma cells with colocalization in nuclei. (vii) A precise cytometric analysis revealed a predominance of apoptosis over the other types of cell death. (viii) Complexes are able to significantly increase ROS generation. (ix) Efficient anticancer action in 3D multicellular tumor spheroids DU145 was demonstrated.

All these data show the unique properties of the Ir^III^–Cu^II^ complexes presented here and their huge potential in various medical treatments. Redox, luminescence, and magnetically active complexes with anticancer effectiveness (preliminarily proved *in vitro*) can be proposed for magnetic resonance imaging (MRI), targeted delivery *via* magnetic field control, targeted destruction of tumor tissue through hyperthermia or reactive oxygen species (phototherapy). We believe that Ir^III^–Cu^II^ compounds are promising agents to further more detailed investigations as anticancer drugs.

## Experimental Section

All data can be found in Supporting Information.

## Supporting Information Available

Structural characterization data; experimental and simulated MS and EPR spectra; IR spectra with description; magnetostructural data with analysis; excitation and emission spectra; cyclic voltamperograms; calculated log *P* values; hydrodynamic diameter; and ζ potentials as well as all experimental data (PDF)
