# Supplementary material for: Liposomal Binuclear Ir(III)–Cu(II) Coordination Compounds with Phosphino-Fluoroquinolone Conjugates for Human Prostate Carcinoma Treatment
Source: Inorg Chem. 2022 Nov 16;61(48):19261–73. doi: 10.1021/acs.inorgchem.2c03015 (PMC9727733; doi:10.1021/acs.inorgchem.2c03015)
Supplement: Supplementary file 1 — ic2c03015_si_001.pdf [file ic2c03015_si_001.pdf]

Supporting Information

Liposomal binuclear Ir(III)-Cu(II) coordination compounds with phosphino-fluoroquinolone conjugates for human prostate carcinoma treatment

Urszula K. Komarnicka,[a†*] Sandra Kozieł,[a†] Barbara Pucelik,[b] Agata Barzowska,[b] Miłosz Siczek,[a] Magdalena Malik,[c] Daria Wojtala,[a] Alessandro Niorettini,[d] Agnieszka Kyzioł,[e] Victor Sebastian,[f,g,h] Pavel Kopel,[i] Stefano Caramori,[d] Alina Bieńko[a]

*corresponding author e-mail urszula.komarnicka@chem.uni.wroc.pl

† First author

[a.] Faculty of Chemistry, University of Wroclaw, Joliot-Curie 14, 50-383 Wroclaw, Poland

[b.]Małopolska Centre of Biotechnology, Jagiellonian University, Gronostajowa 7A, 30-387 Krakow, Poland

[c.] Faculty of Chemistry, Wroclaw University of Science and Technology, WybrzeżeWyspiańskiego 27, 50-370 Wroclaw, Poland

[d.] Department of Chemical, Pharmaceutical, and Agricultural Sciences, University of Ferrara, Via L. Borsari 46, 44121 Ferrara, Italy

[e.] Faculty of Chemistry, Jagiellonian University, Gronostajowa 2, 30-387 Kraków, Poland

[f.]Department of Chemical Engineering, Aragon Institute of Nanoscience (INA), The Aragón Materials Science Institute (ICMA), University of Zaragoza, Campus Río Ebro-Edificio I+D, Mariano Esquillor S/N, 50018 Zaragoza, Spain

[g.]Networking Research Center on Bioengineering, Biomaterials and Nanomedicine, CIBER-BBN, 28-029 Madrid, Spain

[h.]Instituto de Nanociencia y Materiales de Aragón (INMA), CSIC-Universidad de Zaragoza, Zaragoza 50009, Spain

[i.]Department of of Inorganic Chemistry, Faculty of Science, Palacky University, 17. listopadu 12, CZ-771 46 Olomouc, Czech Republic

Compounds under study

1, ([Ir(η[5]-Cp*)Cl_2Pcfx-Cu(phen)](NO_3)·1.75(CH_3OH)·0.75(H_2O), (C51H52Cl2CuFIrN6O6P)·(NO3)·1.75(CH4O)·0.75(H2O) (*M* = 1353.19g mol^{-1}), CCDC 2175515

2, [Ir(η[5]-Cp*)Cl_2Pnfx-Cu(phen)](NO_3)·1.75(CH_3OH)·0.75(H_2O), $C_{52}H_{53}Cl_2CuFIrN_5O_4P$·($NO_3$)·2.75($H_2O$)

(*M* = 1300.15 g mol^{-1}), CCDC 2175516

3, [Ir(η[5]-Cp*)Cl_2Plfx-Cu(phen)](NO_3)·1.3(H_2O)·1.95(CH_3OH), $C_{52}H_{53.5}Cl_2CuF_2IrN_5O_{3.5}P$·($NO_3$)·1.3($H_2O$)·1.95($CH_4O$) (*M* = 1348.02 g mol^{-1}), CCDC 2175517

4, Ir(η[5]-Cp*)Cl_2Psfx-Cu(phen)]), $C_{20}H_{16}N_{10}Co$ (*M* = 1231.69% g mol^{-1})

Table of Contents

Crystallographic data

Table S1 Crystallographic experimental details……………………………………………………..….S5

Figure S1 A perspective view (A and B) of the 1D polymer chain in the crystal structure of **3**…..........….S6

Figure S2 A perspective view of the complex IrPCpCu showing (A) π-stacking interaction between the fluoroquinolone rings (B) packing diagram……………………………………….………S7

Figure S3 A perspective view of the complex IrPNrCu showing (A) π-stacking interaction between the fluoroquinolone rings (B) packing diagram…………………………………………………….S7

Figure S4 Packing diagram of complex IrPLmCu………………………………………………….….S7

Figure S5 Packing diagram of complex 1 showing (A) π-stacking interaction between the fluoroquinolone rings (B) offset pattern of the π–π stacking in complex 1…………………………………….…S8

Figure S6 Packing diagram of complex IrPNrCu showing (A) π-stacking interaction between the fluoroquinolone rings (B) offset pattern of the π–π stacking in complex IrPNrCu……………..…S8

Mass Spectrometry

Figure S7

(a) ESI mass spectrum of IrPLmCu(phen). ESI(+)MS in CH_3OH, m/z: 1191.175 [IrPLmCu(phen)]$^+$; 1149.285 [IrPLmCu(phen)-HCl-Cl+CH_2OH]$^+$; 948.229 [IrPLmCu(phen)-

Cu(phen)+H]$^+$; 912.253 [IrPLmCu(phen)-Cu(phen)-Cl]$^+$; 908.303 [IrPLmCu(phen)-Cu(phen)-2Cl+CH_2OH]$^+$; 878.293[IrPLmCu(phen)-Cu(phen)-2Cl+H]$^+$; 791.191[IrPLmCu(phen)-$IrCl_2$]$^+$; 593.131 [IrPLmCu(phen)-$IrCl_2$-PPh_2CH_2]$^+$……………………………………………….…….S9

(b)experimental and simulated spectra of [IrPLmCu(phen)+H]$^+$……………………………..S10

(c) experimental and simulated spectra of[IrPLmCu(phen)-2Cl-2H+CH_3OH]$^+$………………..S10

(d) experimental and simulated spectra of [IrPLmCu(phen)-Cu(phen)]$^+$……………………..…S11

(e)experimental and simulated spectra of [IrPLmCu(phen)-Cu(phen)-Cl]$^+$……………………..…S11

(f)experimental and simulated spectra of [IrPLmCu(phen)-Cu(phen)-2Cl-H+CH_3OH]$^+$…..…S12

(g)experimental and simulated spectra of[IrPLmCu(phen)-Cu(phen)-2Cl+H]$^+$…………….....…S12

(h)experimental and simulated spectra of [IrPLmCu(phen)-$IrCl_2$]$^+$……………………………..S13

(i)experimental and simulated spectra of[IrPLmCu(phen)-$IrCl_2$-PPh_2CH_2]$^+$…………….……..…S13

Figure S8

(a) ESI mass spectrum of IrPCpCu(phen). ESI(+)MS in CH_3OH, m/z: 1129.264 [M-HCl-Cl+CH_2OH]$^+$; 1099.260 [M-2Cl]$^+$; 858.280 [M-2Cl-Cu(phen)+H]$^+$; 787.176 [M-$IrCpCl_2$+O]$^+$; 573.122 [M-$IrCpCl_2$-$PPhCH_2$]$^+$; 564.131 [M-2HCl+CH_2OH]$^{2+}$; 549.126 [M-HCl-Cl]$^{2+}$………..S14

(b) experimental and simulated spectra of [M-HCl-Cl+CH_2OH]$^+$……………………….…..S15

(c) experimental and simulated spectra of[M-2Cl]$^+$…………………………...……………...S15

(d) experimental and simulated spectra of [M-2Cl-Cu(phen)+H]$^+$………………………………S16

(e) experimental and simulated spectra of [M-$IrCpCl_2$+O]$^+$……………………….……………S16

(f) experimental and simulated spectra of[M-$IrCpCl_2$-$PPhCH_2$]$^+$…………….…………………S17

(g) experimental and simulated spectra of [M-2HCl+CH_2OH]$^{2+}$………………..……………S17

(h)experimental and simulated spectra of [M-HCl-Cl]$^{2+}$……………………………………..….S18

Figure S9

(a) ESI mass spectrum of IrPNrCu(phen). ESI(+)MS in CH_3OH, m/z: 1157.199 [M]$^+$; 1123.246 [M-Cl+H]$^+$; 916.222 [M-Cu(phen)+H]$^+$; 894.226 [M-HCl-OH+CH_2OH]$^+$; 759.183 [M-$IrCpCl_2$]$^+$….S19

(b) experimental and simulated spectra of [M]$^+$……………………………………………….…..S20

(c) experimental and simulated spectra of [M-Cl+H]$^+$………………………………….……....S20

(d) experimental and simulated spectra of [M-Cu(phen)+H]$^+$……………………………………..S21

(e) experimental and simulated spectra of [M-HCl-OH+CH_2OH]$^+$…………………….…………S21

(f) experimental and simulated spectra of [M-$IrCpCl_2$]$^+$……………………………...…………S22

Figure S10

(a) ESI mass spectrum of IrPSfCu(phen). ESI(+)MS in CH_3OH, m/z: 1230.208 $[M]^+$; 989.243 $[M-Cu(phen)+H]^+$; 947.300 $[M-Cu(phen)-2HCl+CH_2OH]^+$; 919.320 $[M-Cu(phen)-2Cl+H]^+$; 832.210 $[M-IrCpCl_2]^+$; 634.158 $[M-IrCpCl_2-PPhCH_2]^+$; 594.651 $[M-2HCl+CH_2OH]^{2+}$……………….……..S23

(b) experimental and simulated spectra of $[M]^+$…………………………………………………...S24
(c) experimental and simulated spectra of $[M-Cu(phen)+H]^+$…………………………………….S24
(d) experimental and simulated spectra of $[M-Cu(phen)-2HCl+CH_2OH]^+$…………………….…S25
(e) experimental and simulated spectra of $[M-Cu(phen)-2Cl+H]^+$……………………………….……S25
(f) experimental and simulated spectra of $[M-IrCpCl_2]^+$………………………………………..S26
(g) experimental and simulated spectra of $[M-IrCpCl_2-PPhCH_2]^+$………………………..……S26
(h) experimental and simulated spectra of $[M-2HCl+CH_2OH]^+$……………………………….……S27

Infrared spectroscopy

Table S2 The characteristic discussed bands in FT-IR spectra of complexes 1-4……………………….……S28
Figure S11
a The FT-FIR spectra of complexes 1-4…………………………………………………...……S29
b The FT-IR spectra of complexes 1-4 in the MIR spectral region………………………………..S30
c The FT-IR spectra of $Cu(phen)(NO_3)_2$ in the MIR and FIR spectral regions…………...………S31

EPR measurements

Figure S12 The X-band EPR spectra of 1 - 4 at 77 K together with the spectrum calculated by computer simulation of the experimental spectra with spin Hamiltonian parameters given in the text……………………………………….S32
Figure S13 EPR frozen solution spectra (at 77 K) of compounds 1 - 4; in DMSO solvent together with the theoretical spectrum calculated using the parameters given in the text……………S33

Luminescence properties

Figure S14 Emission spectra obtained for IrPLmCu(Phen) and RuPLmCu(Phen) both in DMF and DMSO solution……………...….S34
Figure S15 Normalized emission spectra for heteronuclear Ir(III)/Cu(II) complexes,

homonuclear Ir(III) complexes and the corresponding phosphine ligands

λex = 340 nm, 298 K……………………………….….S34

Figure S16 Excitation spectra of IrPCpCu, IrPNrCu, IrPLmCu and IrPSfCu……………………….……S35

Magneto-structural studies

Figure S17 Field dependencies of the AC susceptibility components for 2 at T = 2.0 K for a set of frequencies of the AC field. Lines are a guide for the eye………………………………..S37

Figure S18 Temperature dependence of (a) the in-phase and (b) out-of-phase molar susceptibility of 2………………..……….S38

Electrochemical study

Figure S19 Cyclic voltammetry of the mononuclear fragments of Iridium complexes in DMF (50mV/s from -2.1 to +1.6 vs SCE, $TBAPF_6$ 0.1M as supporting electrolyte)…………………..….S39

Biological study

Table S3. Calculated log P values for ligands using program ACD/log P[1] and complexes using program ALOGPS 2.150.(1-3)……………………………….……..S39

Table S4. Hydrodynamic diameter and Zeta potential determined by DLS technique as well as loading content and encapsulation efficiency determined by ICP-MS technique for selected liposome formulations……………………….………..….S40

Experimental Section ……………………………………………………………………..…..S40

Table S1. Crystallographic experimental details			
Parameters	**IrPNrCu·(NO_3)·1.75(CH_4O)·0.75(H_2O)**	**IrPCpCu·(NO_3)·2.75(H_2O)**	**IrPLmCu·(NO_3)·1.3(H_2O)·1.95(CH_4O)**
Moiety Formula	$(C_{51}H_{52}Cl_2CuFIrN_6O_6P)\cdot(NO_3)\cdot1.75(CH_4O)\cdot0.75(H_2O)$	$C_{52}H_{53}Cl_2CuFIrN_5O_4P\cdot(NO_3)\cdot2.75(H_2O)$	$C_{52}H_{53.5}Cl_2CuF_2IrN_5O_{3.5}P\cdot(NO_3)\cdot1.3(H_2O)\cdot1.95(CH_4O)$
Formula weight (g·mol^{-1})	1353.19	1300.15	1348.02
Crystal description	blue	pale green	green
Crystal size (mm)	0.26 × 0.12 × 0.04	0.25 × 0.10 × 0.05	0.28 × 0.11 × 0.06
Temperature (K)	293	100	100
Type of radiation	Cu Kα	Cu Kα	Cu Kα
Crystal system	Triclinic	Triclinic	Orthorhombic
Space group	*P-1*	*P-1*	P*bcn*
a (Å)	10.801 (2)	10.531 (3)	48.765 (5)
b (Å)	13.114 (3)	15.039 (4)	15.889 (2)
c (Å)	20.180 (5)	18.734 (5)	14.660 (3)
α (°)	100.01 (2)	66.38 (3)	
β (°)	99.95 (2)	82.87 (3)	
γ (°)	91.82 (2)	78.35 (2)	
Volume (Å^3)	2766.9 (11)	2659.4 (14)	11359 (3)
Z	2	8	8
Density calc. (mg/m^3)	1.624	1.624	1.577
Absorptioncoeff. (mm^{-1})	6.81	7.028	6.63
F(000)	1364	1309	5445
θmin – θmax (°)	4.2–66.9	3.2–74.2	3.9 – 67.5
hkl ange	12 ← h ← - 12<br>15 ← k ← - 14<br>23 ← l ← - 24	13← h ← - 13<br>17 ← k ← - 18<br>21 ← l ← - 23	58 ← h ← - 58<br>16 ← k ← - 19<br>17 ← l ← - 17
Reflections collected	33507	43007	63264
Independent reflections	9814	10849	10196

R_{int}	0.037	0.034	0.053
Completeness to θ_{full} (%)	99.5	99.7	99.8
Absorptioncorrectiontype	analytical	gaussian	gaussian
T_{max} and T_{min}	0.773, 0.389	1.000, 0.320	1.000, 0.304
Data/restraints/parameters	9814/6/786	10849/0/877	10196/36/855
Goodness of fit F^2	1.240	1.039	1.127
R_1, wR_2 [I>2σ(I)]	0.0442, 0.1082	0.0508, 0.1250	0.0630, 0.1677
R_1, wR_2 (all data)	0.0455, 0.109	0.0601, 0.132	0.0665,
Largest diff. peak and hole (eÅ^{-3})	0.99, -0.89	2.03, -3.49	2.29, −2.31

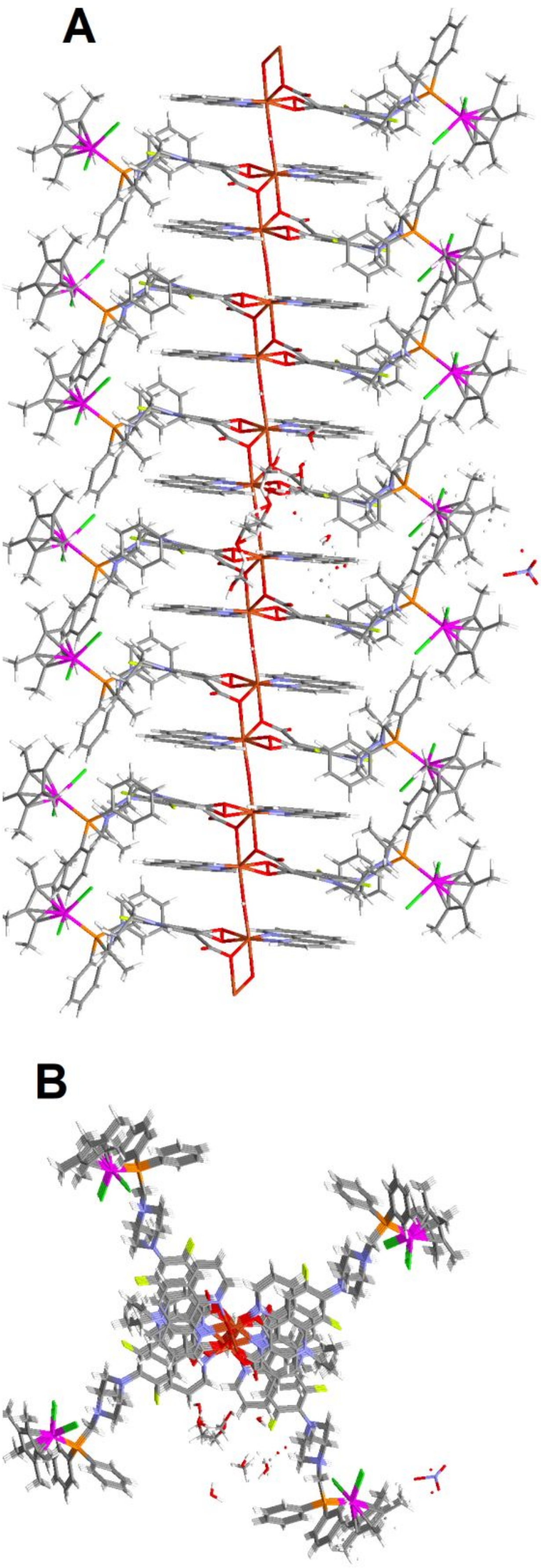

Figure S1 A perspective view (A and B) of the 1D polymer chain in the crystal structure of **3**.

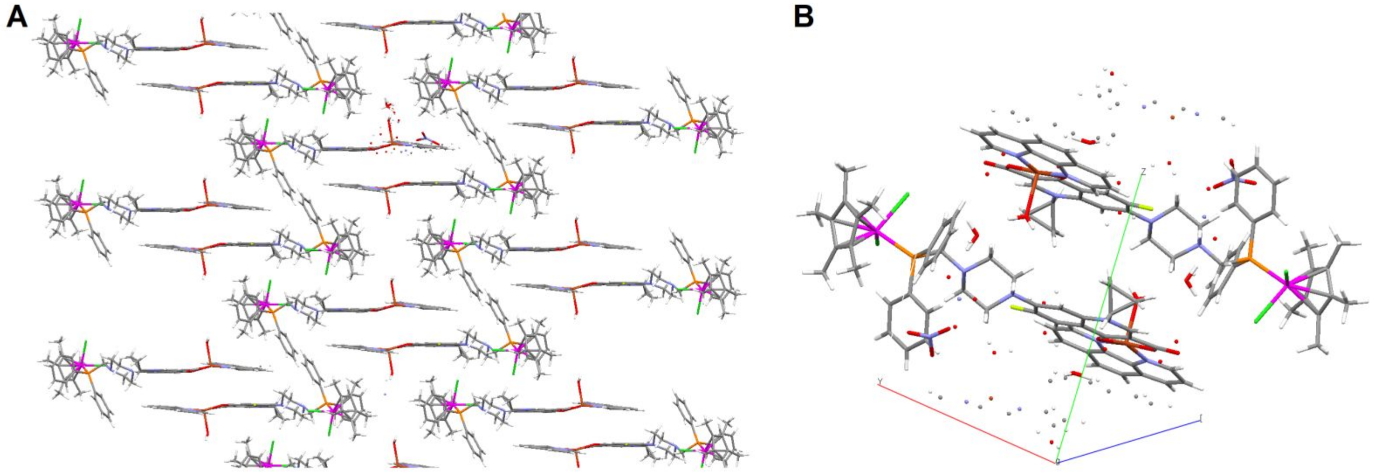

Figure S2 A perspective view of the complex **IrPCpCu** showing (A) π-stacking interaction between the fluoroquinolone rings (B) packing diagram.

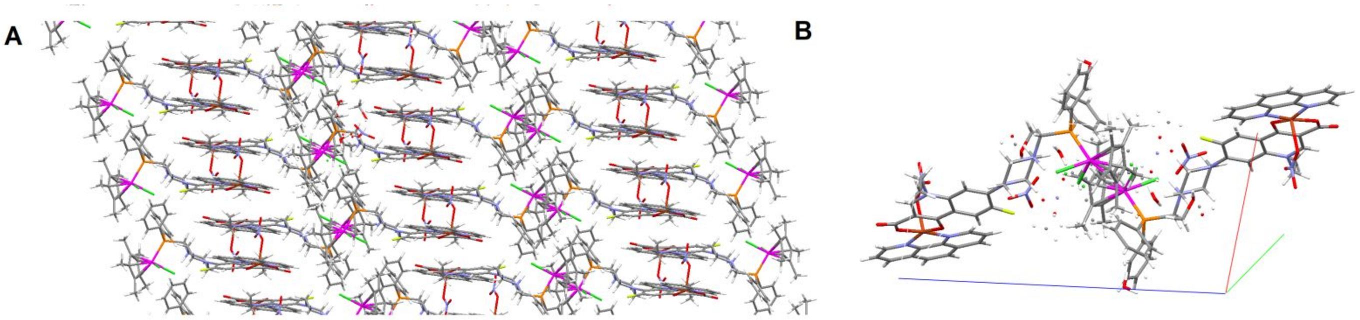

Figure S3 A perspective view of the complex **IrPNrCu** showing (A) π-stacking interaction between the fluoroquinolone rings (B) packing diagram.

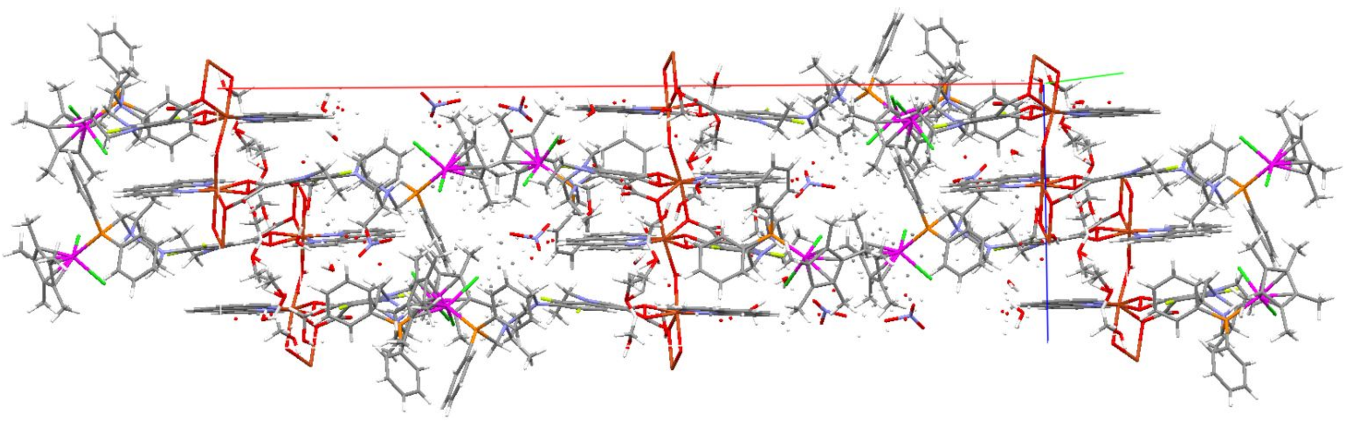

Figure S4 Packing diagram of complex **IrPLmCu**

Additionally, the binuclear unit is stabilized by π-stacking interactions between phenanthroline and lomefloxacin fragments.

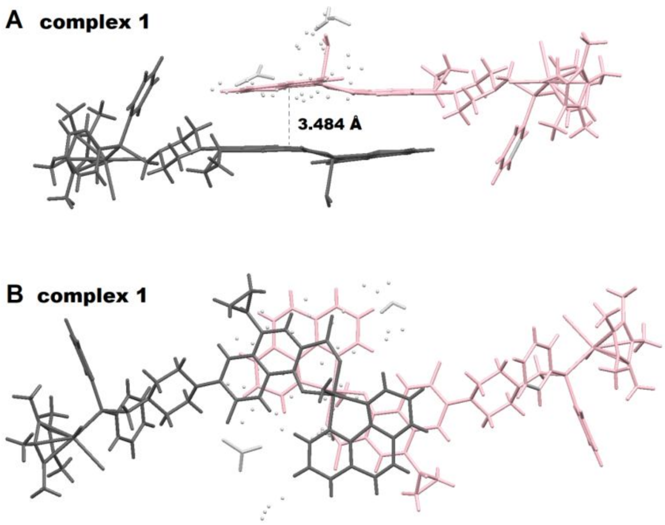

Figure S5 Packing diagram of complex **1** showing (A) π-stacking interaction between the fluoroquinolone rings (B) offset pattern of the π–π stacking in complex **1**.

Interactions between two independent fused-ring moieties: the fluoroquinolone fragment ~~rings~~ and the phen ligands can be found as well in cases of **1** and **2**. As it could be expected, these interactions led to interesting molecular packing of the inorganic compounds stabilized by π-stacking interactions (Fig. S5, S6). The distances from the two centroids of ciprofloxacin (complex **1**) or norfloxacin (complex **2**) to the phen plane are 3.484 and 3.375 Å, respectively.

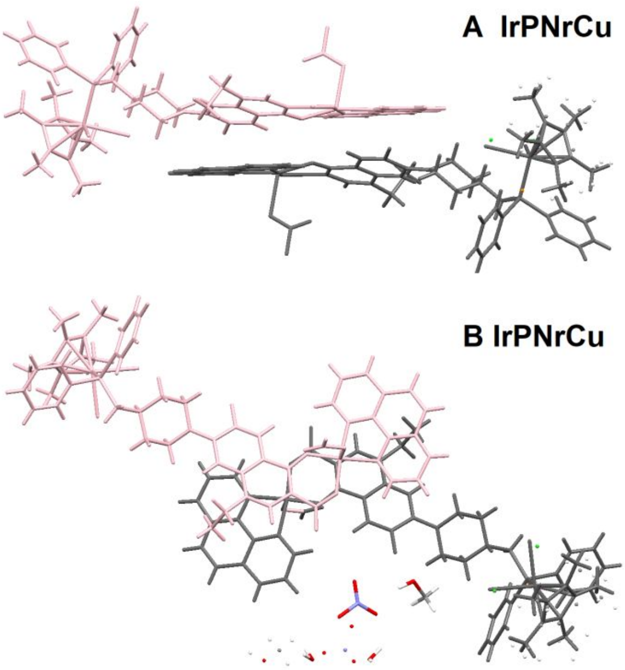

Figure S6 Packing diagram of complex **IrPNrCu** showing (A) π-stacking interaction between the fluoroquinolone rings (B) offset pattern of the π–π stacking in complex **IrPNrCu**.

Figure S7 (a)

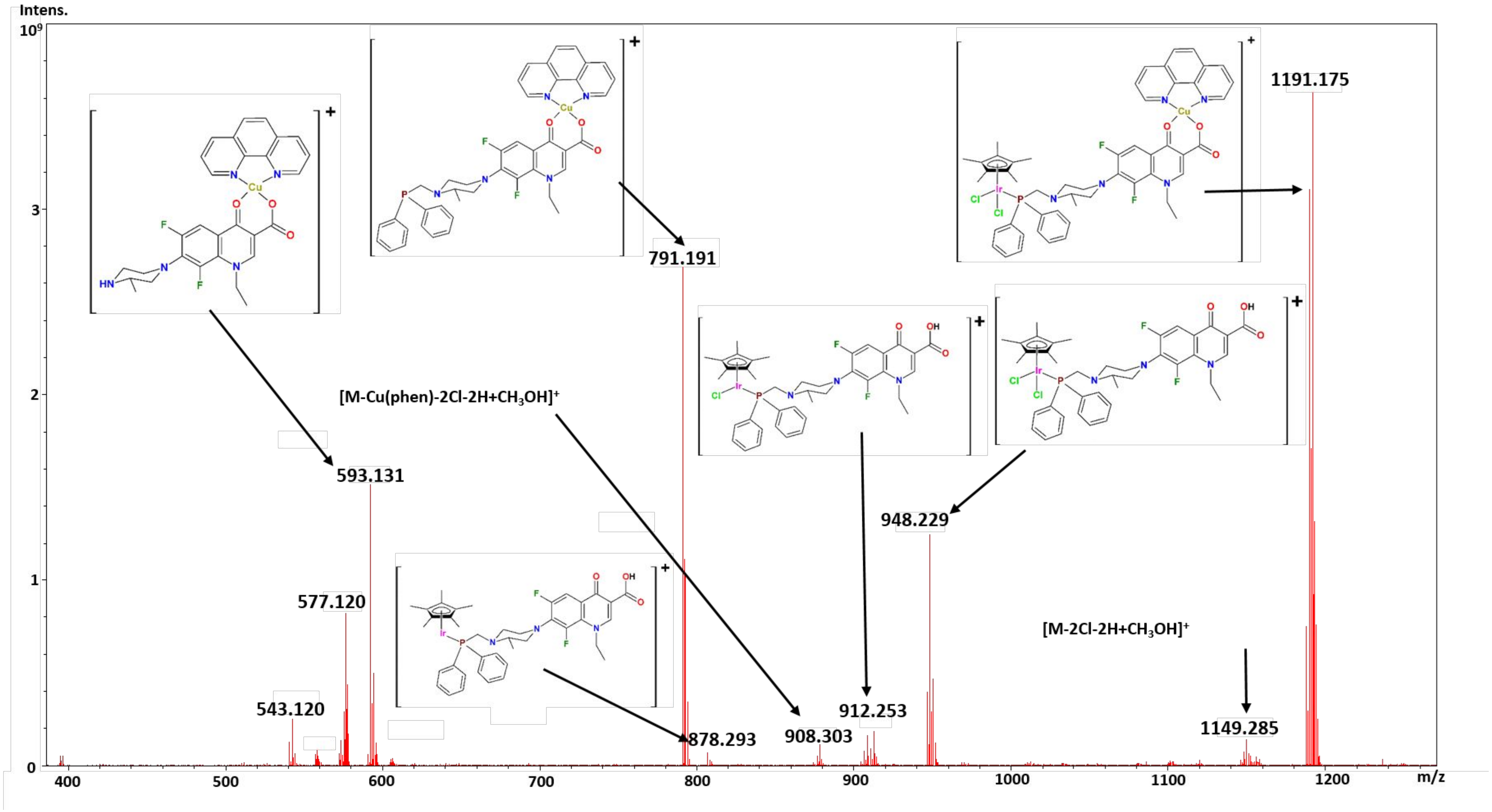
Intens.
10^9
3
2
1
0
400
500
600
700
800
900
1000
1100
1200
m/z
593.131
791.191
1191.175
[M-Cu(phen)-2Cl-2H+CH3OH]+
948.229
577.120
543.120
878.293
908.303
912.253
[M-2Cl-2H+CH3OH]+
1149.285

Figure S7 (b)

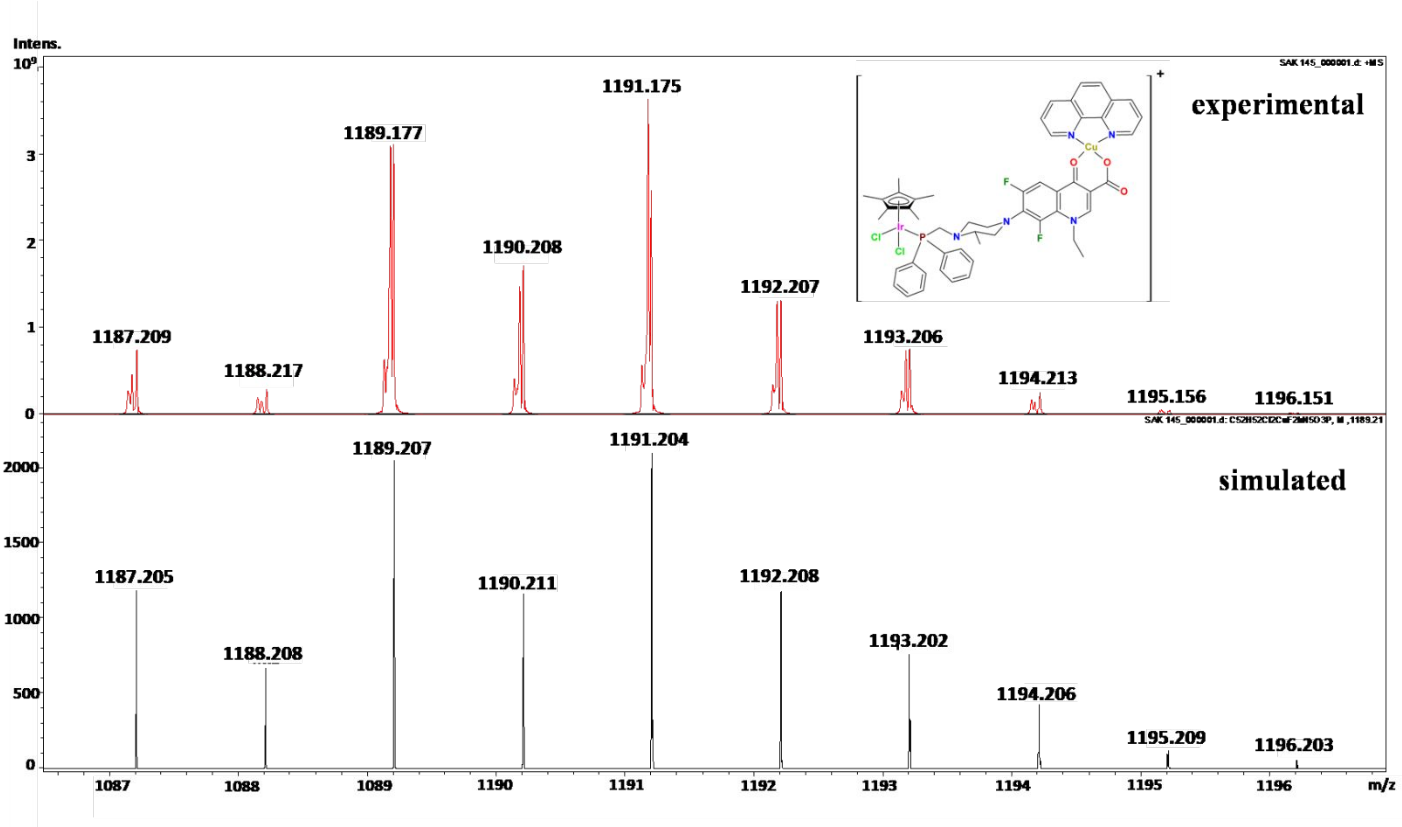

Figure S6 (c)

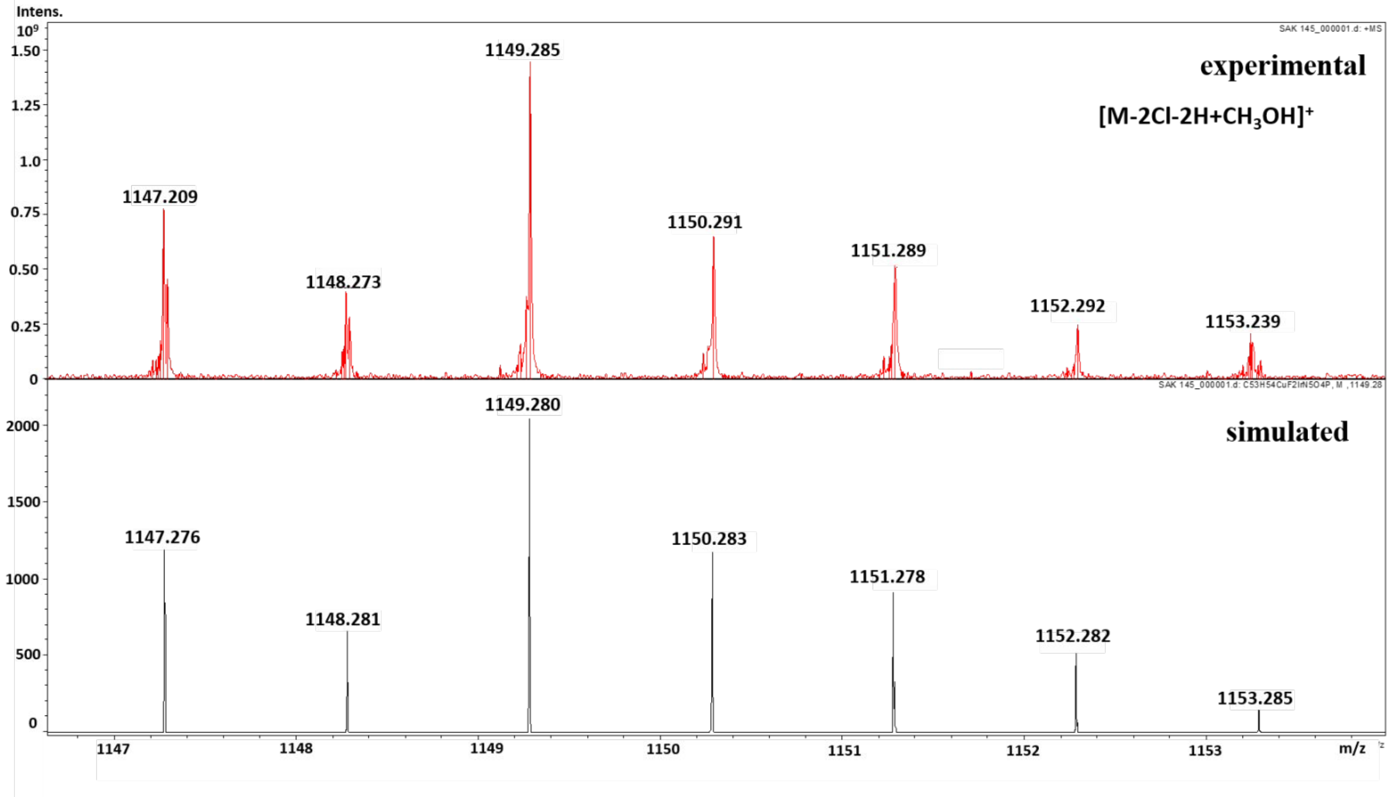

Figure S7 (d)

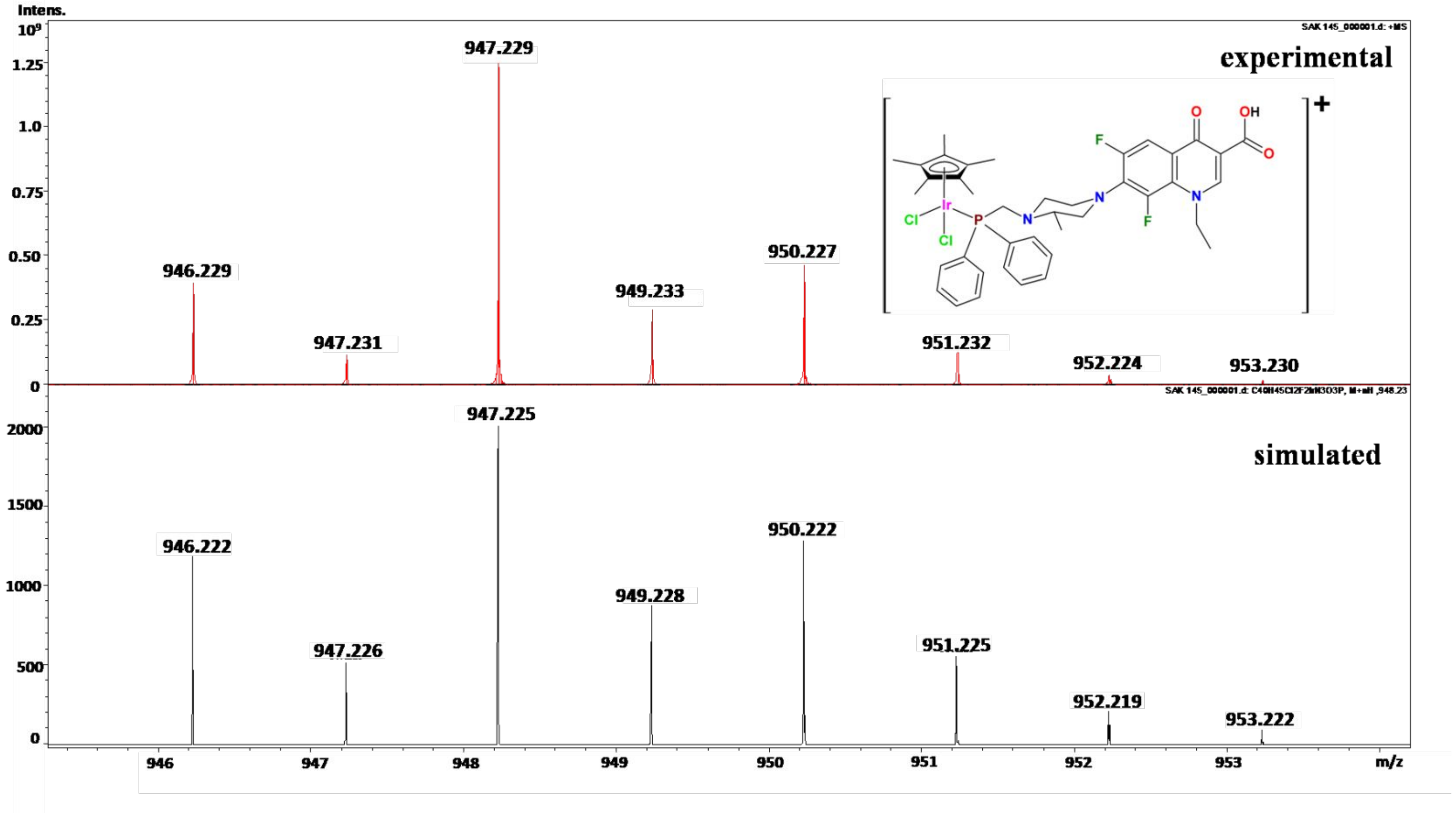

Figure S7 (e)

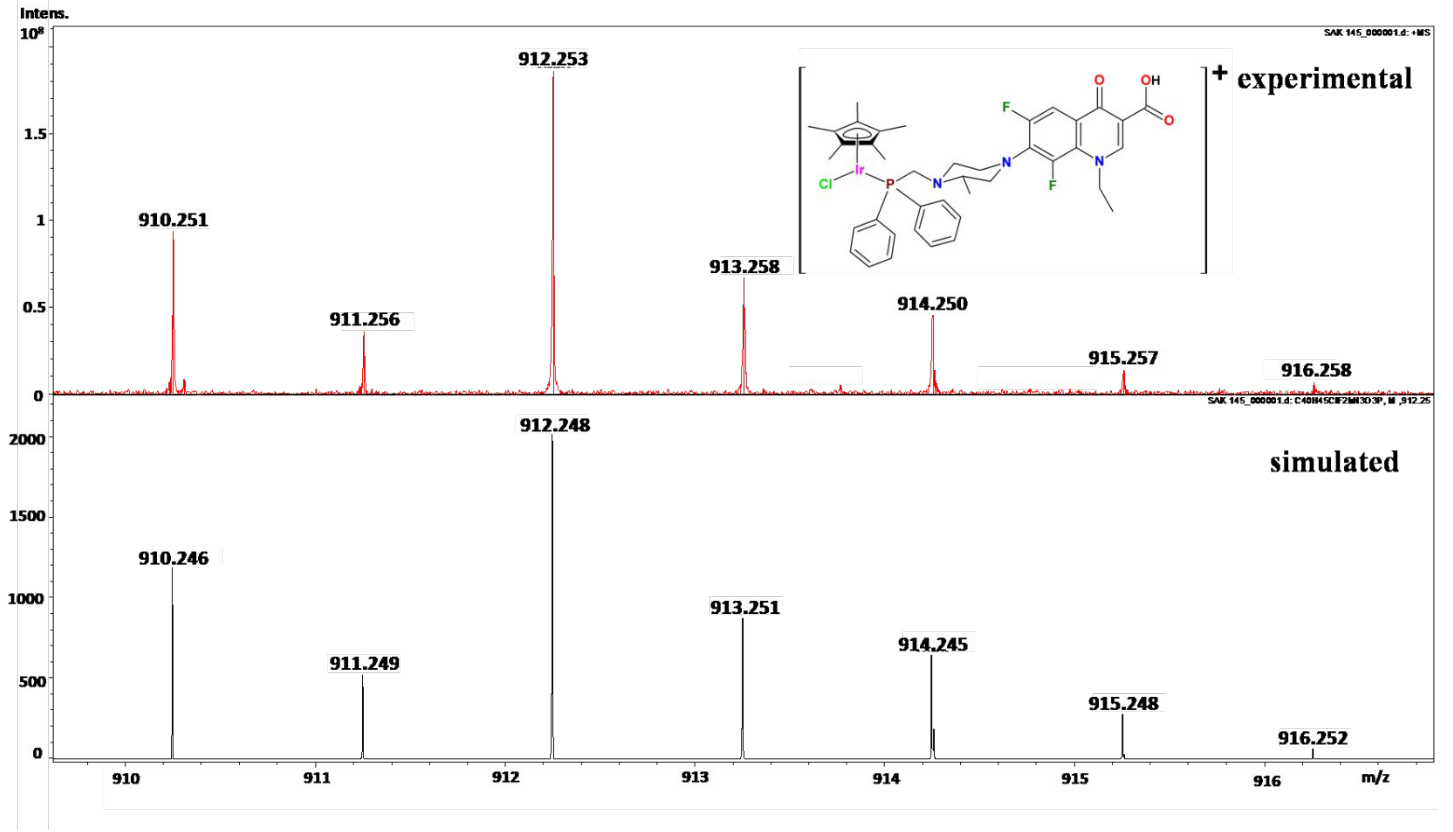

Figure S7 (f)

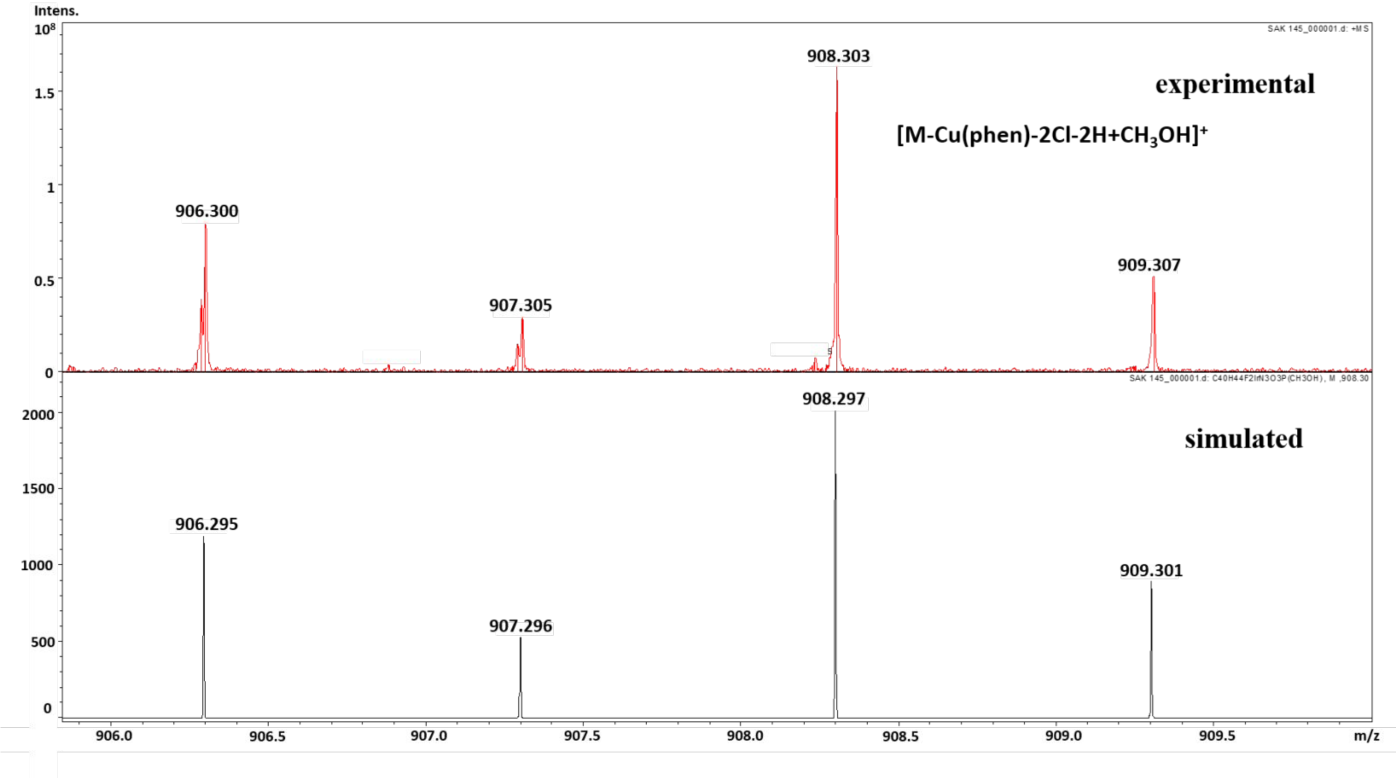

Figure S7 (g)

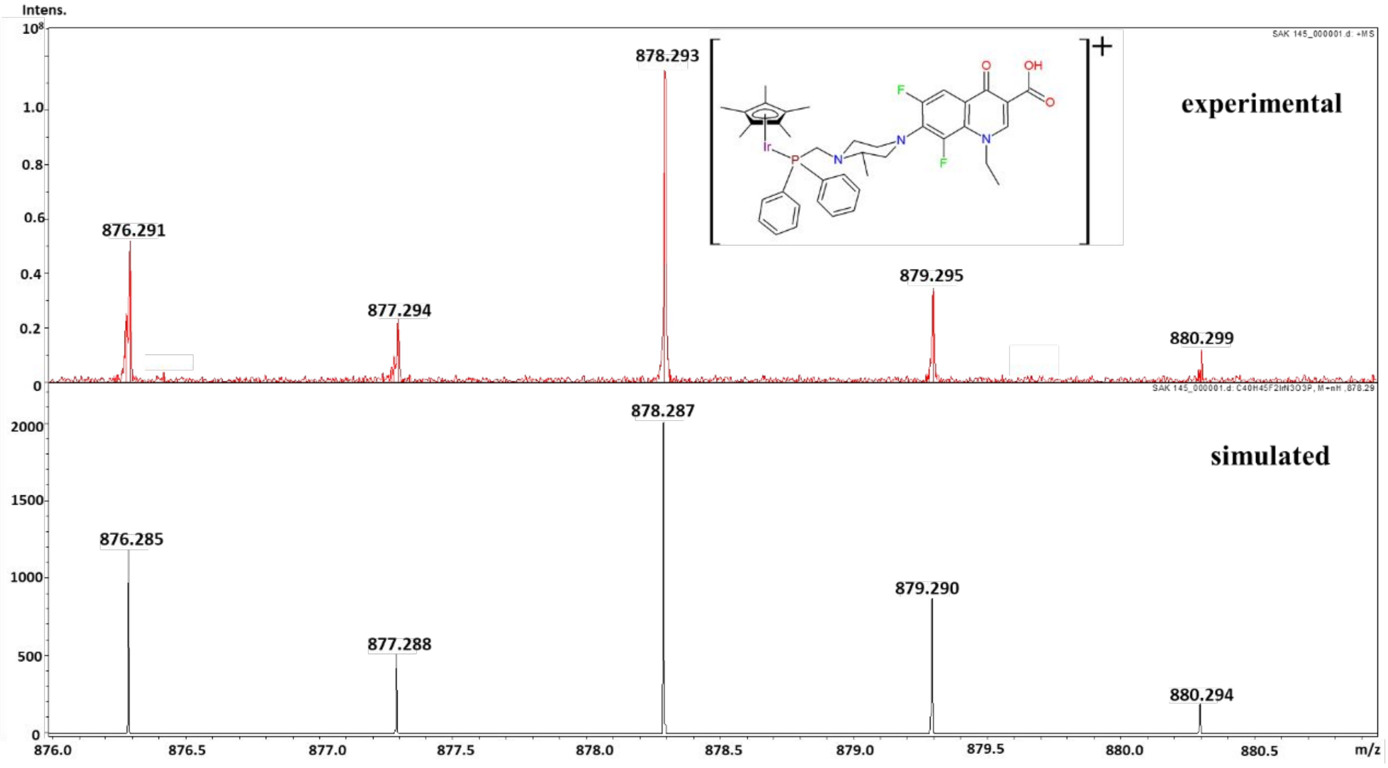

Figure S7 (h)

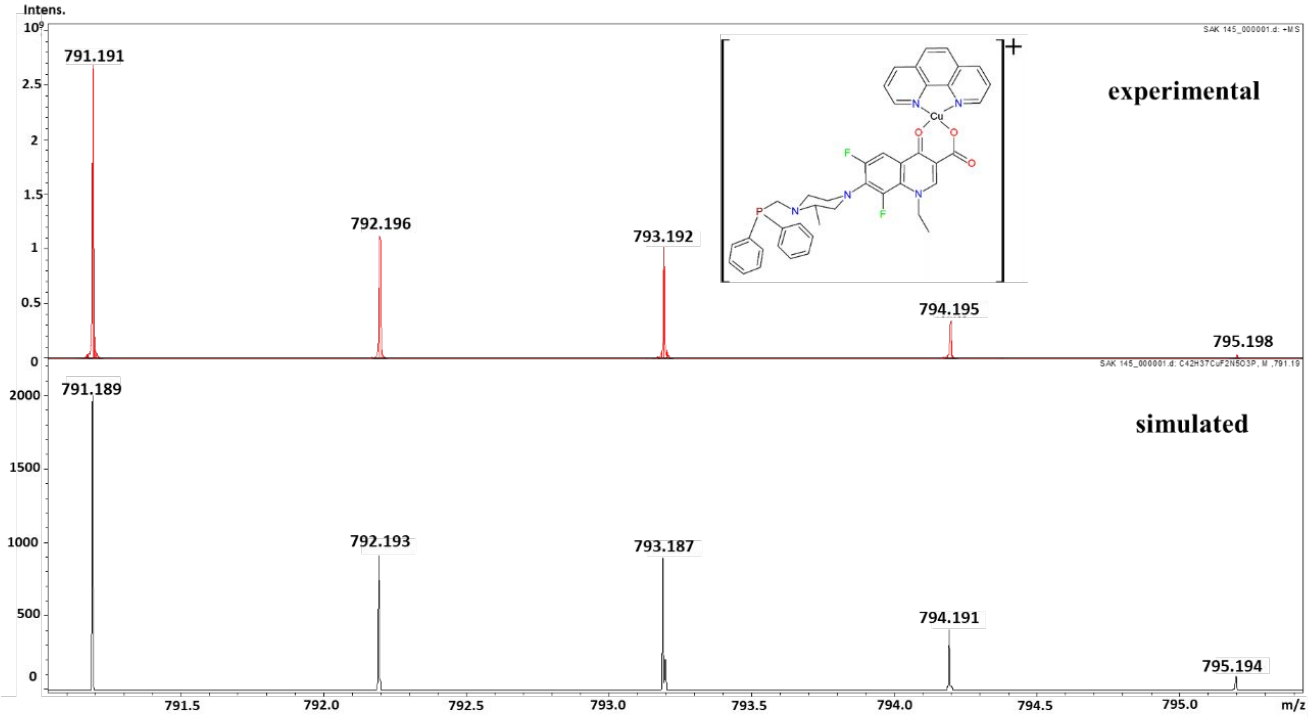

Figure S7 (i)

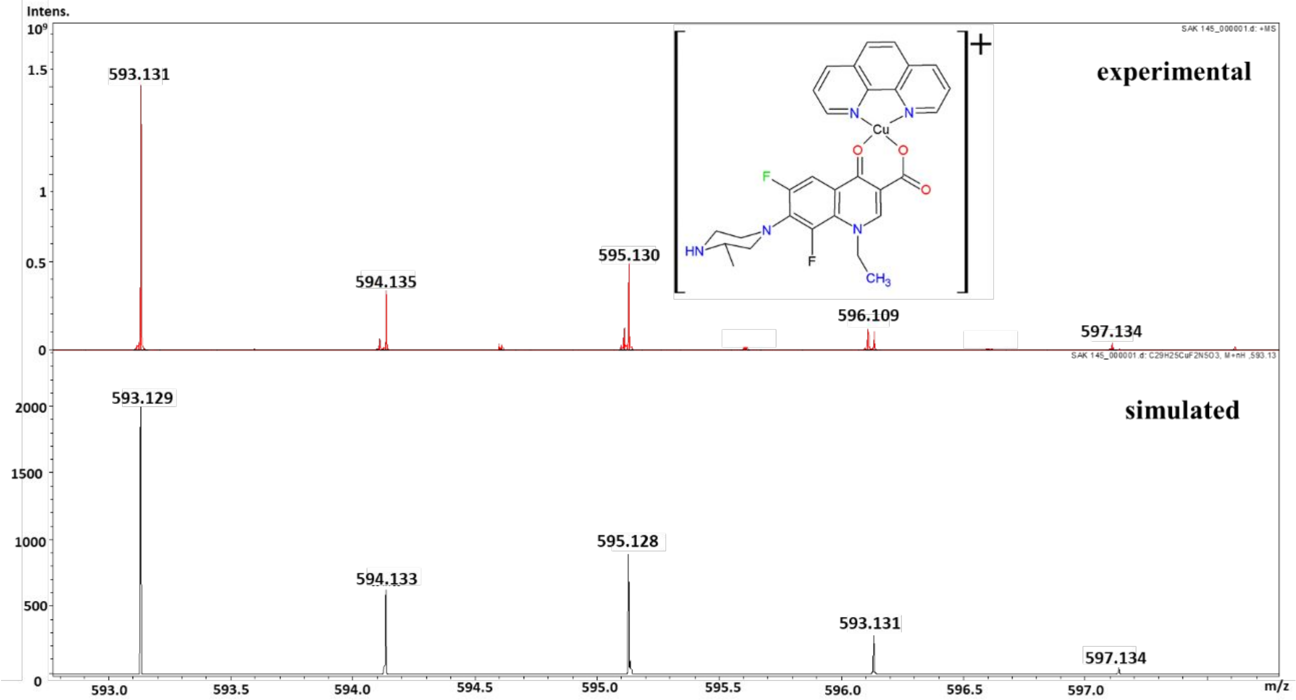

Figure S7 (a) ESI mass spectrum of **IrPLmCu(phen)**. ESI(+)MS in CH_3OH, m/z: 1191.175 [**IrPLmCu(phen)**]$^+$; 1149.285 [**IrPLmCu(phen)-**HCl-Cl+CH_2OH]$^+$; 948.229 [**IrPLmCu(phen)**-Cu(phen)+H]$^+$; 912.253 [**IrPLmCu(phen)-**Cu(phen)-Cl]$^+$; 908.303 [**IrPLmCu(phen)-**Cu(phen)-2Cl+CH_2OH]$^+$; 878.293[**IrPLmCu(phen)-**Cu(phen)-2Cl+H]$^+$; 791.191[**IrPLmCu(phen)**-$IrCl_2$]$^+$; 593.131 [**IrPLmCu(phen)**-$IrCl_2$-PPh_2CH_2]$^+$**(b)**experimental and simulated spectra of [**IrPLmCu(phen)**+H]$^+$**(c)** experimental and simulated spectra of[**IrPLmCu(phen)-**2Cl-2H+CH_3OH]$^+$**(d)** experimental and simulated spectra of [**IrPLmCu(phen)**-Cu(phen)]$^+$**(e)**experimental and simulated spectra of [**IrPLmCu(phen)-**Cu(phen)-Cl]$^+$**(f)**experimental and simulated spectra of [**IrPLmCu(phen)-**Cu(phen)-2Cl-H+CH_3OH]$^+$**(g)**experimental and simulated spectra of[**IrPLmCu(phen)-**Cu(phen)-2Cl+H]$^+$**(h)**experimental and simulated spectra of[**IrPLmCu(phen)**-$IrCl_2$]$^+$**(i)**experimental and simulated spectra of[**IrPLmCu(phen)**-$IrCl_2$-PPh_2CH_2]$^+$

Fig. S8 (a)

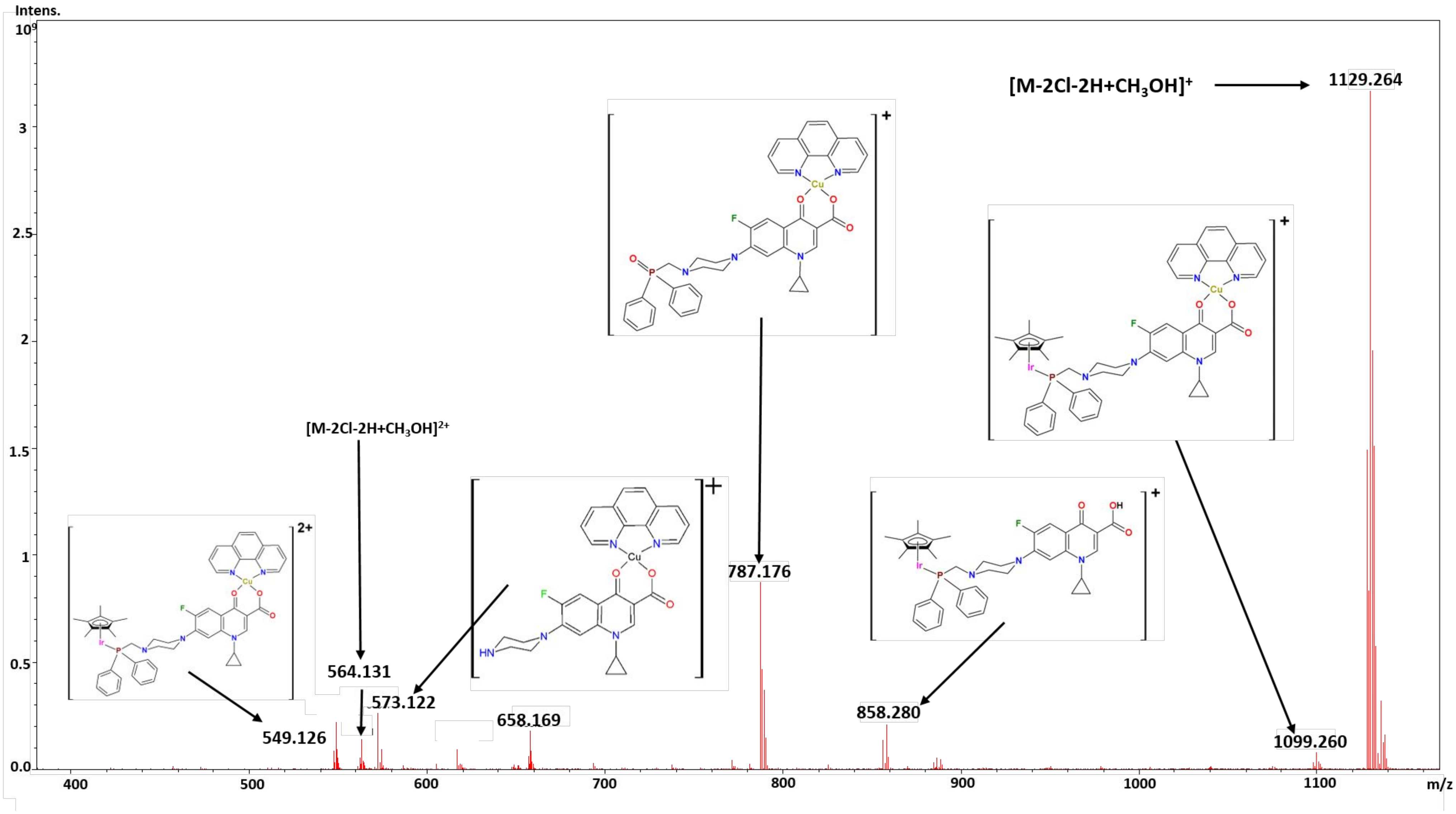

Intens.
10^9
3
2.5
2
1.5
1
0.5
0.0
400
500
600
700
800
900
1000
1100
m/z
[M-2Cl-2H+CH_3OH]$^+$
1129.264
[M-2Cl-2H+CH_3OH]$^{2+}$
564.131
549.126
573.122
658.169
787.176
858.280
1099.260
2+
+

Fig. S8 (b)

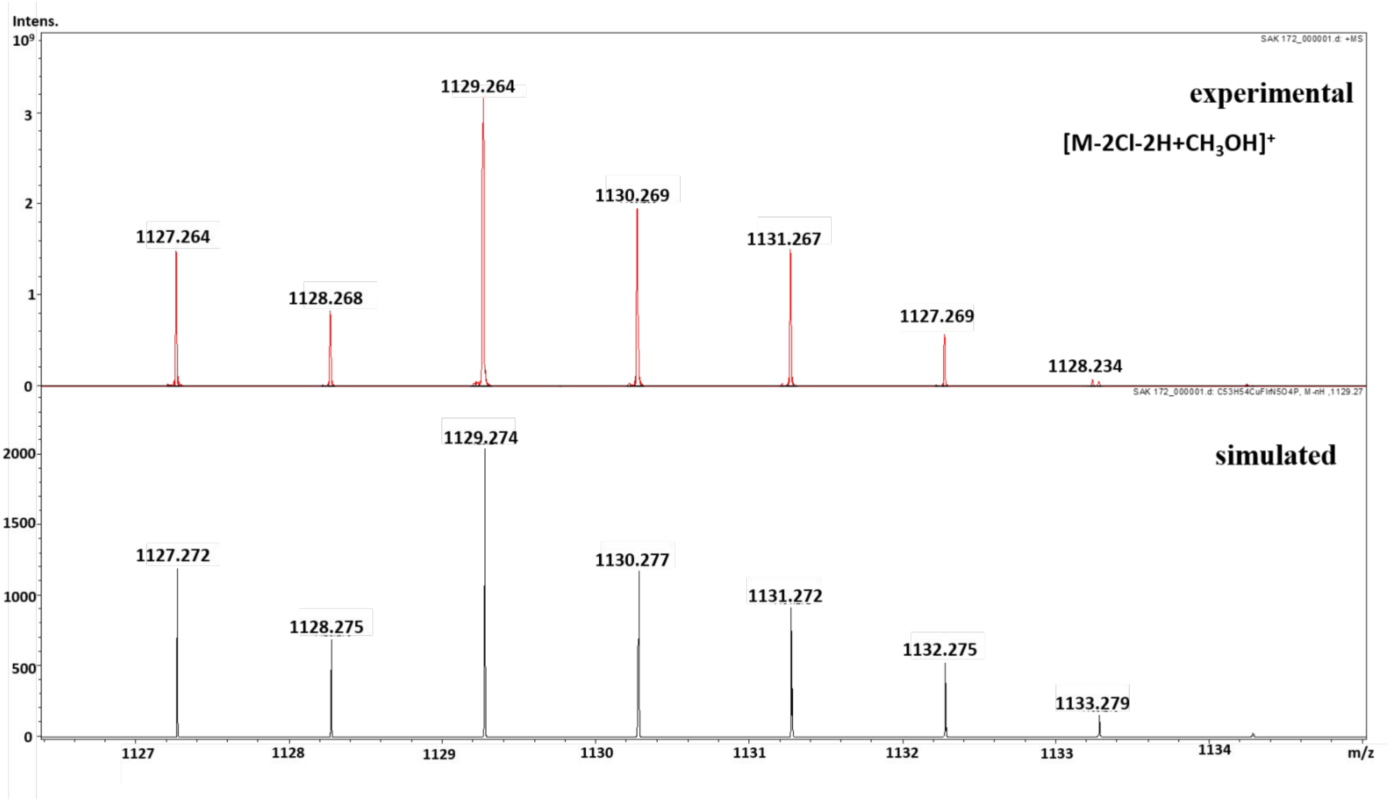

Fig. S8 (c)

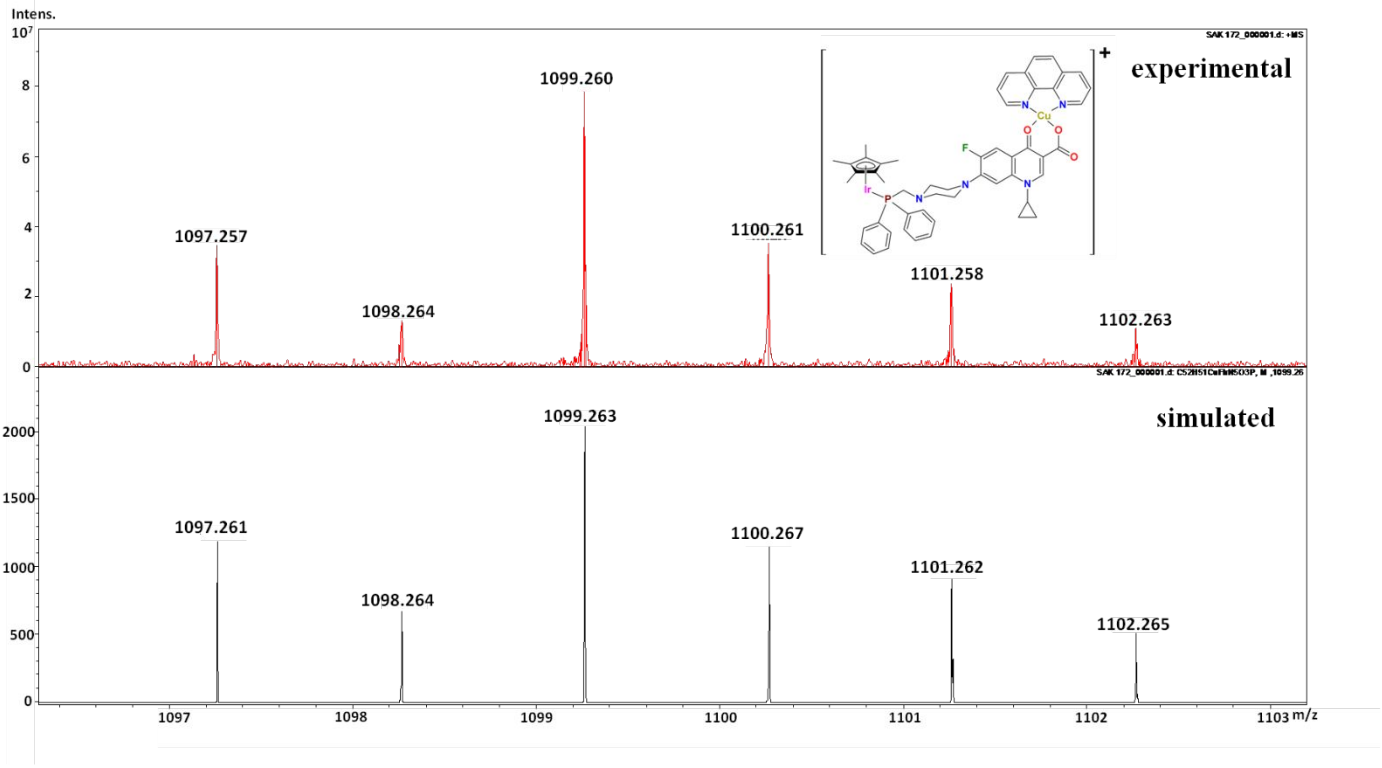

Fig. S8 (d)

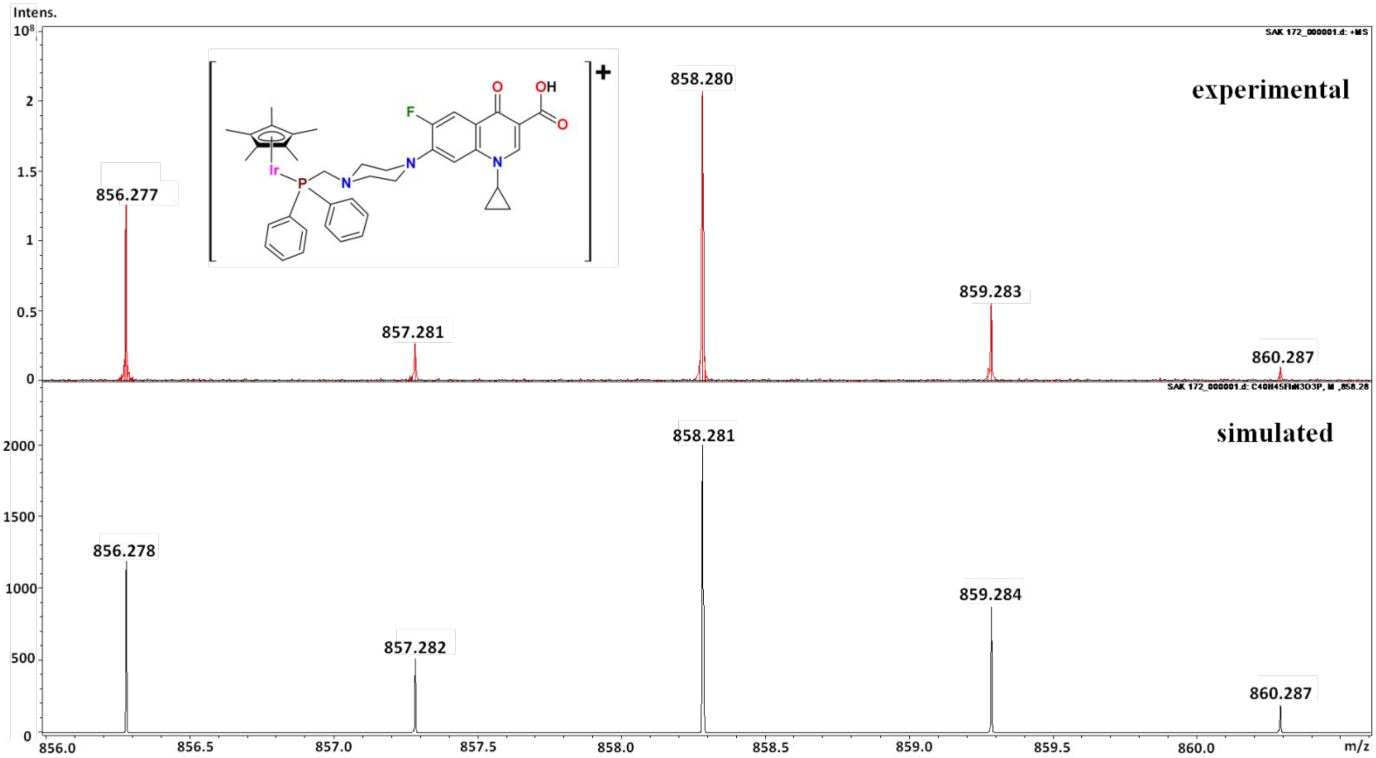

Fig. S8 (e)

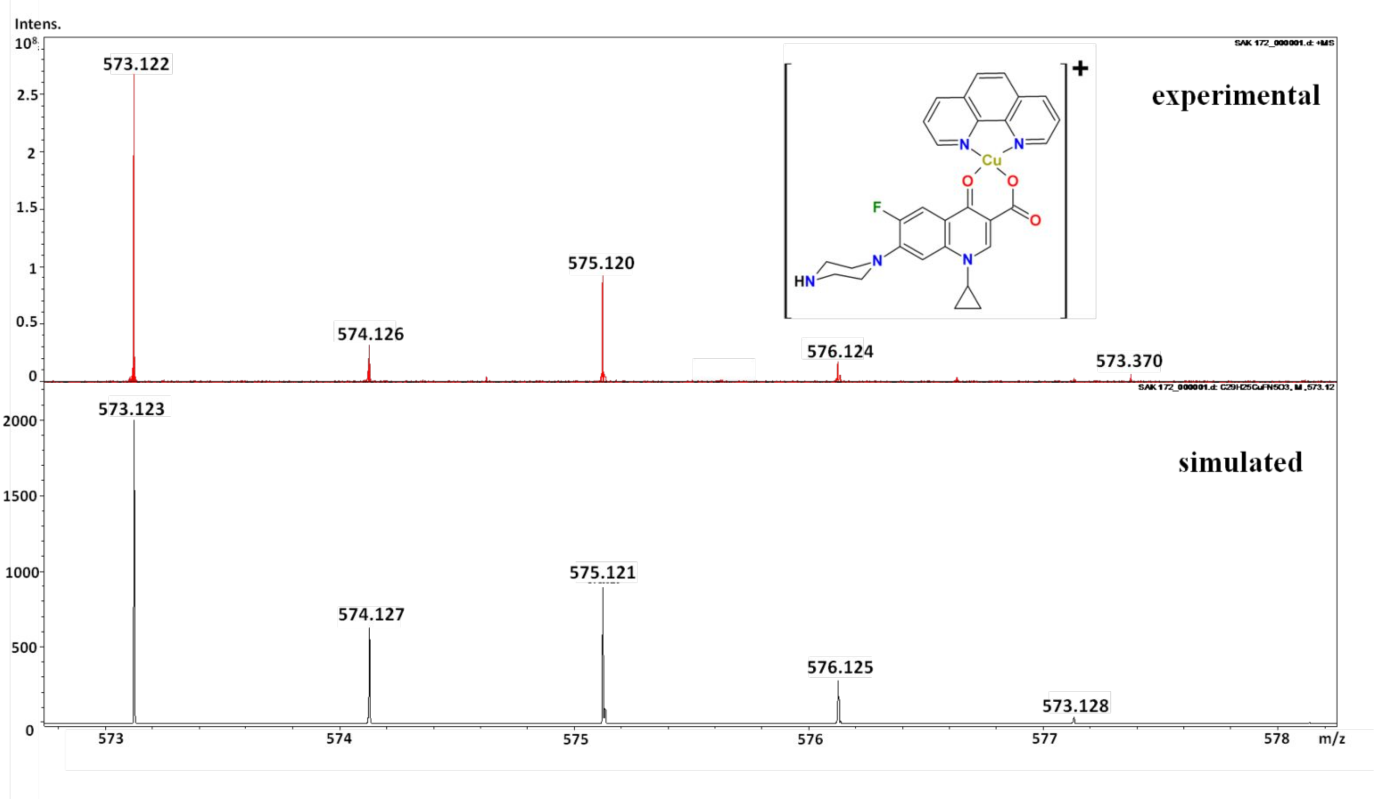

Fig. S8 (f)

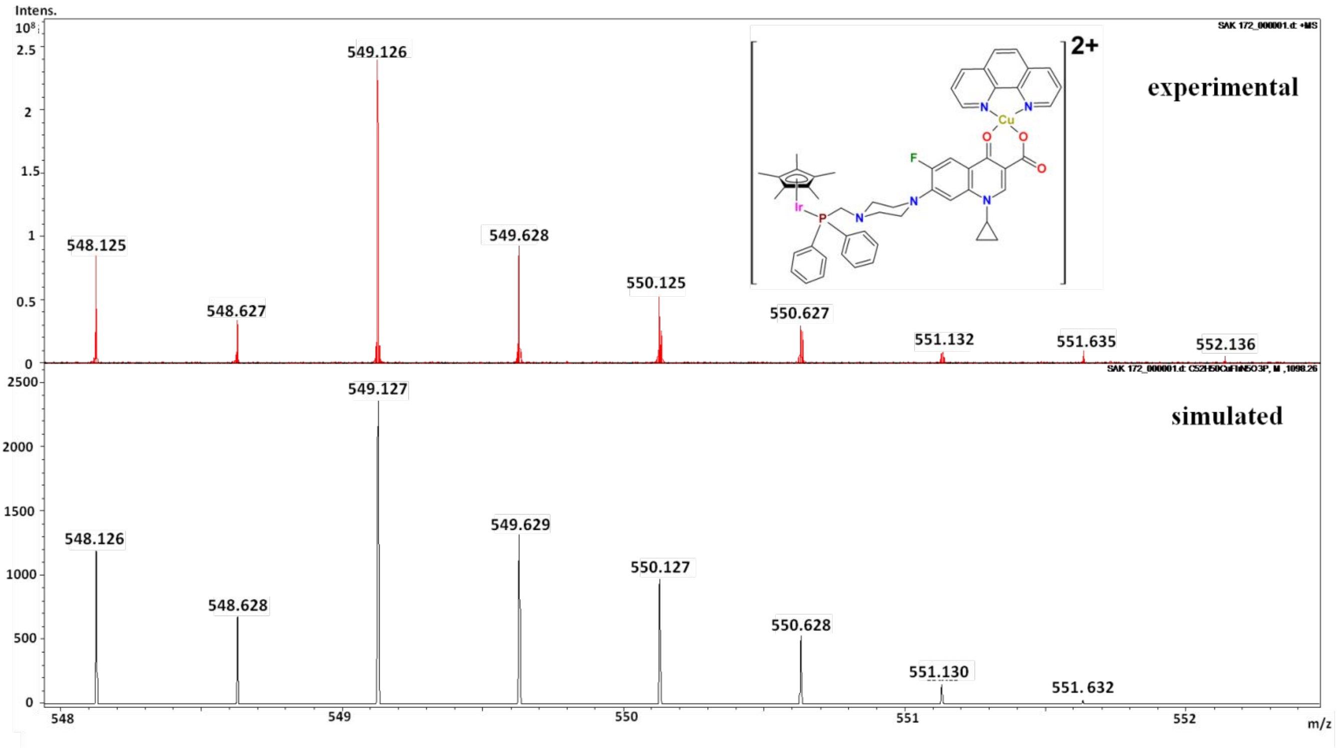

Fig. S8 (g)

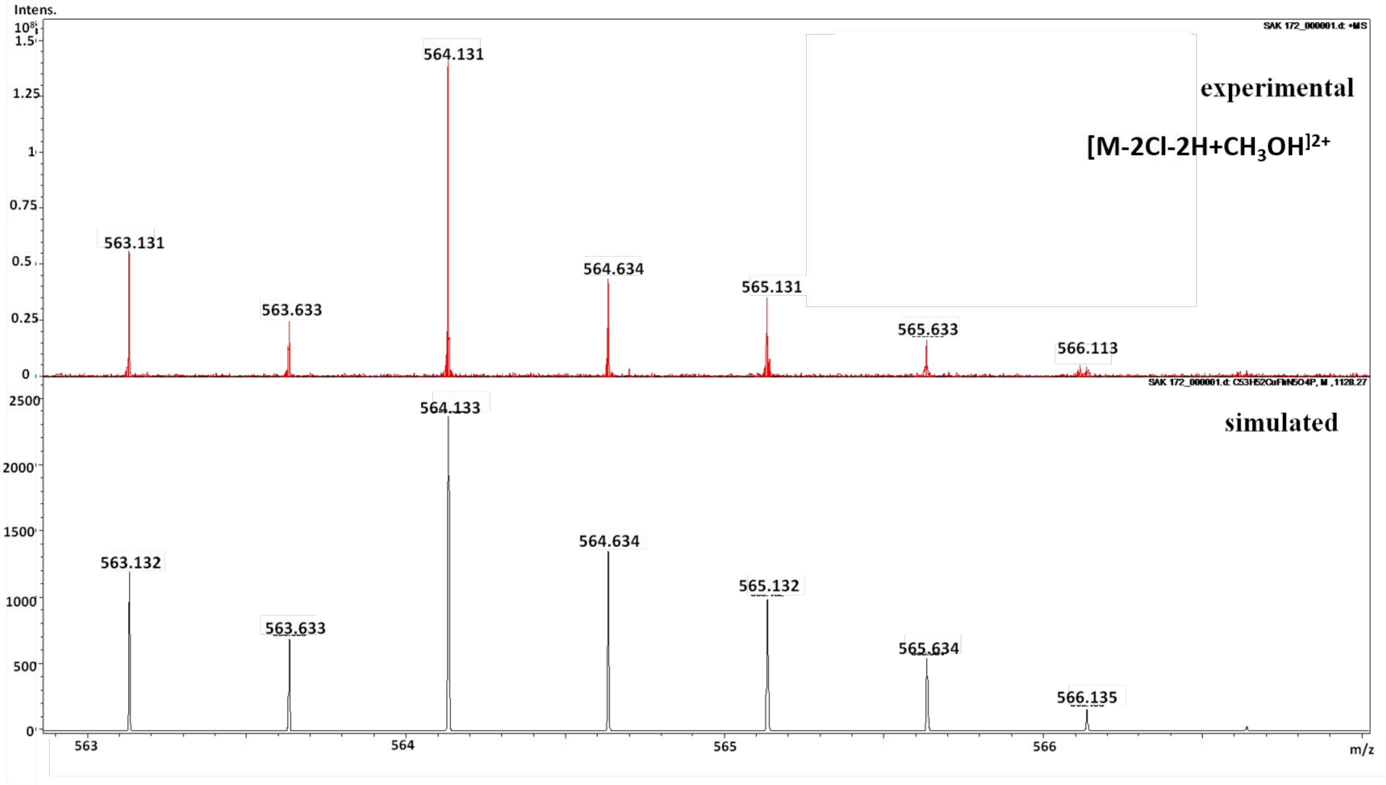

Fig. S8 (h)

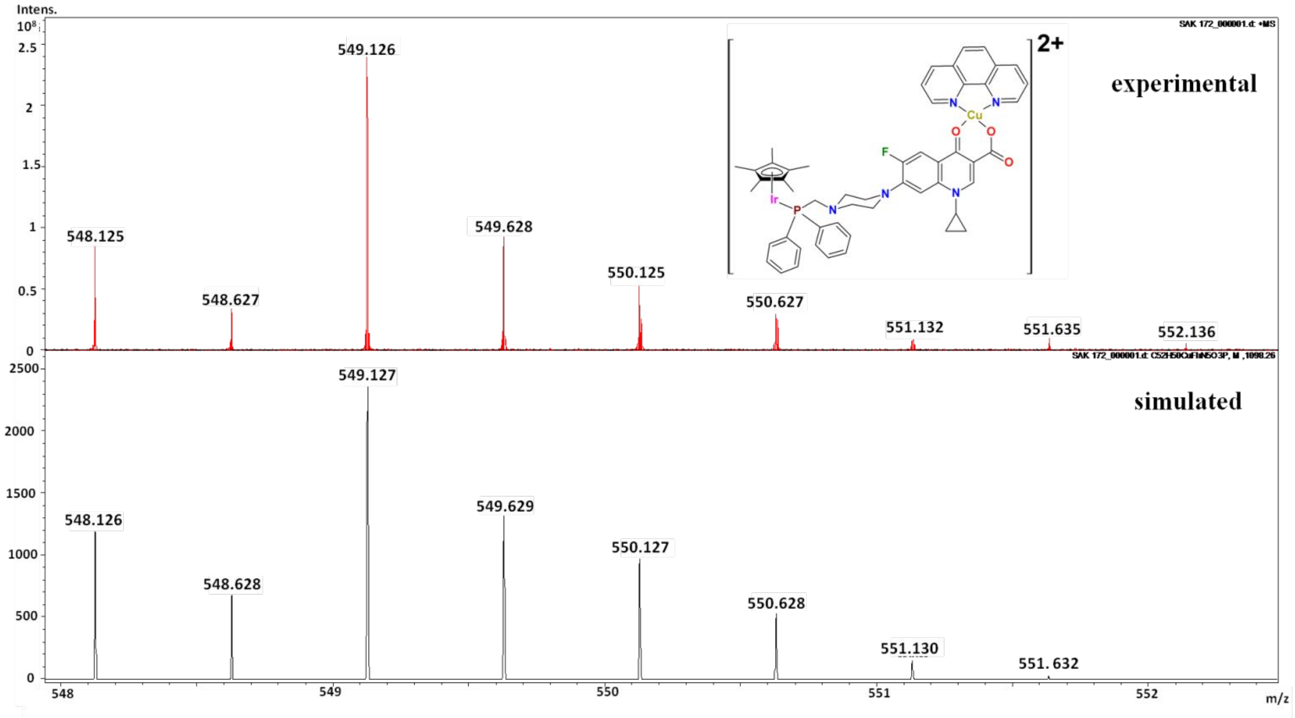

Figure S8 (a) ESI mass spectrum of **IrPCpCu(phen)**. ESI(+)MS in CH_3OH, m/z: 1129.264 [**M**-HCl-Cl+CH_2OH]$^+$; 1099.260 [**M**-2Cl]$^+$; 858.280 [**M**-2Cl-Cu(phen)+H]$^+$; 787.176 [**M**-IrCpCl_2+O]$^+$; 573.122 [**M**-IrCpCl_2-PPhCH_2]$^+$; 564.131 [**M**-2HCl+CH_2OH]$^{2+}$; 549.126 [**M**-HCl-Cl]$^{2+}$ **(b)**experimental and simulated spectra of [**M**-HCl-Cl+CH_2OH]$^+$**(c)** experimental and simulated spectra of[**M**-2Cl]$^+$**(d)** experimental and simulated spectra of [**M**-2Cl-Cu(phen)+H]$^+$**(e)**experimental and simulated spectra of [**M**-IrCpCl_2+O]$^+$**(f)**experimental and simulated spectra of[**M**-IrCpCl_2-PPhCH_2]$^+$**(g)** experimental and simulated spectra of [**M**-2HCl+CH_2OH]$^{2+}$**(h)**experimental and simulated spectra of [**M**-HCl-Cl]$^{2+}$

Fig. S9 (a)

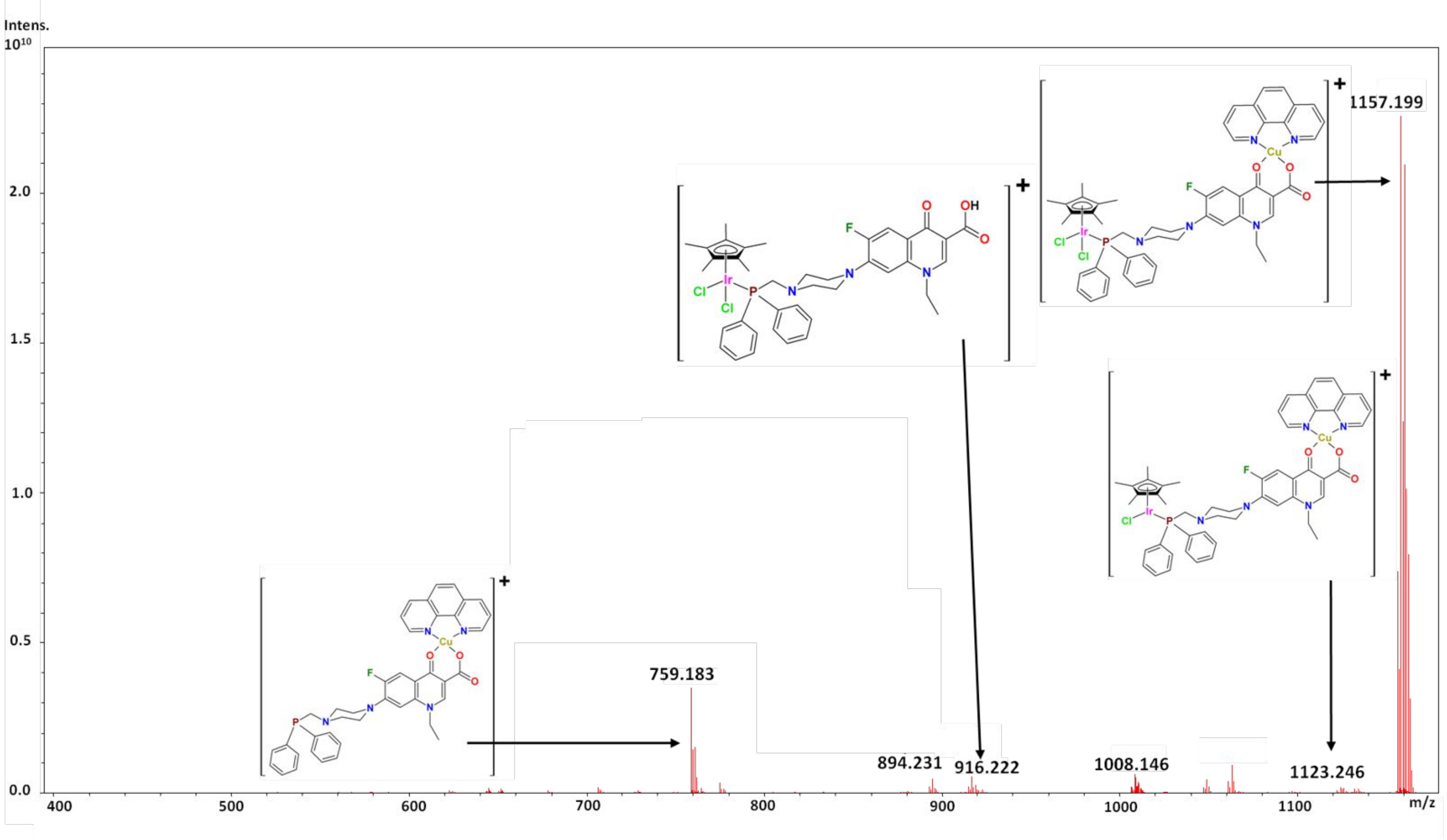

Intens.
10^10
2.0
1.5
1.0
0.5
0.0
400
500
600
700
800
900
1000
1100
m/z
759.183
894.231
916.222
1008.146
1123.246
1157.199
+
Ir
Cl
P
N
F
O
OH
Cu

Fig. S9 (b)

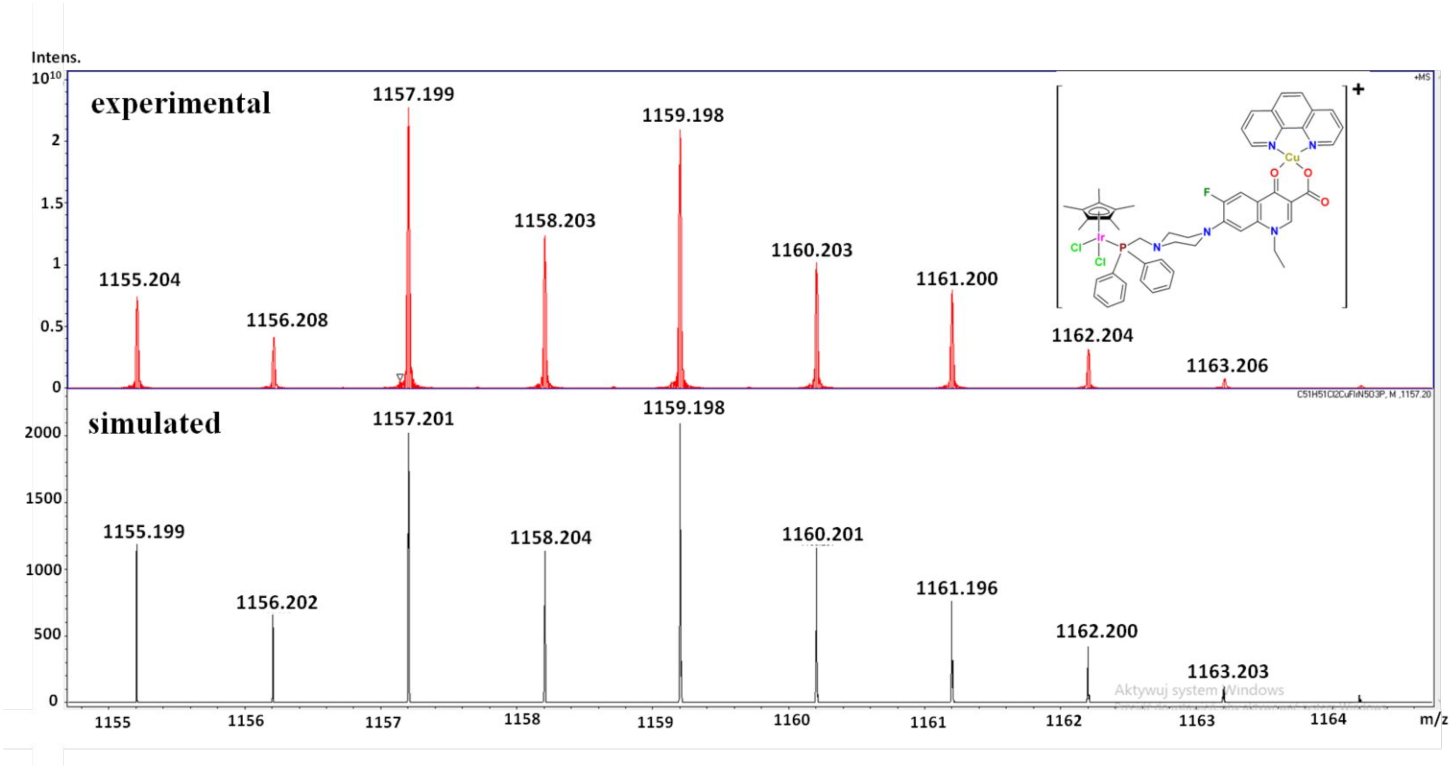

Fig. S9 (c)

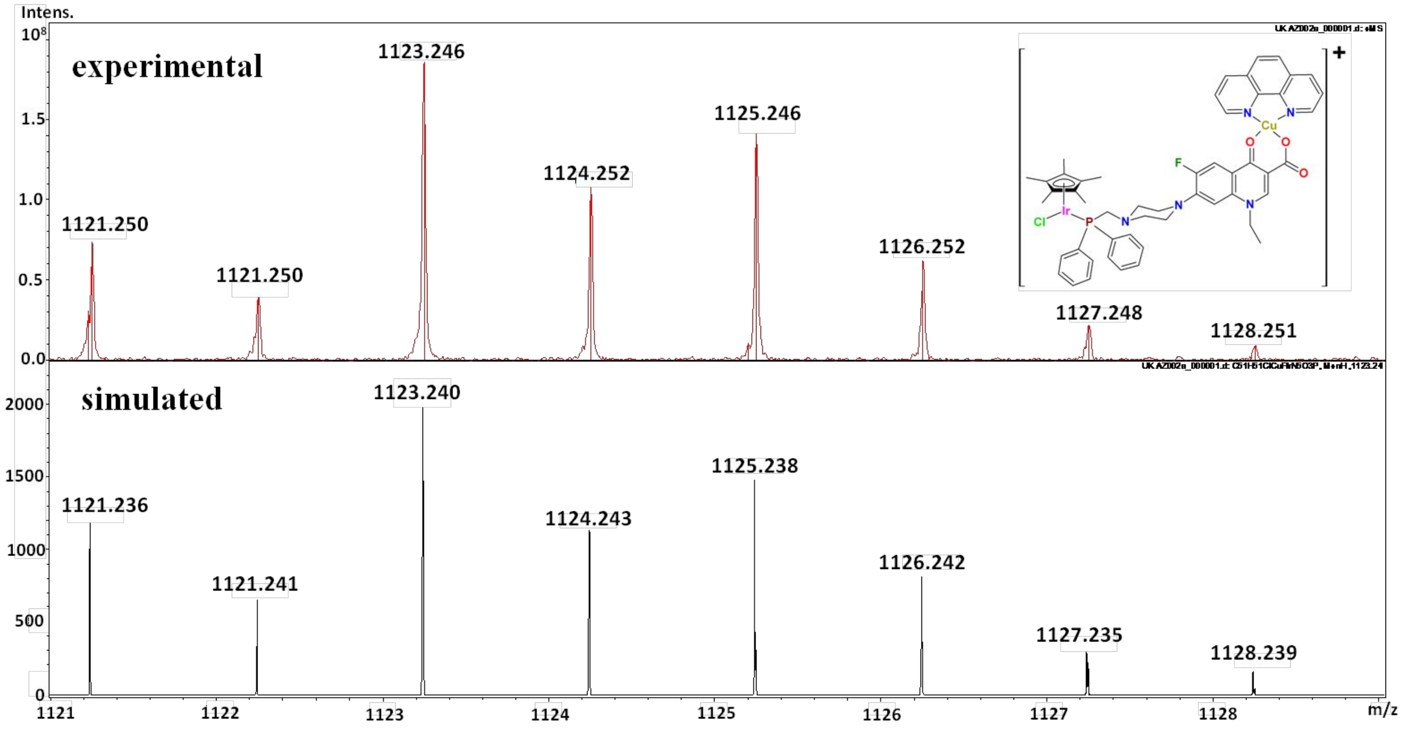

Fig. S9 (d)

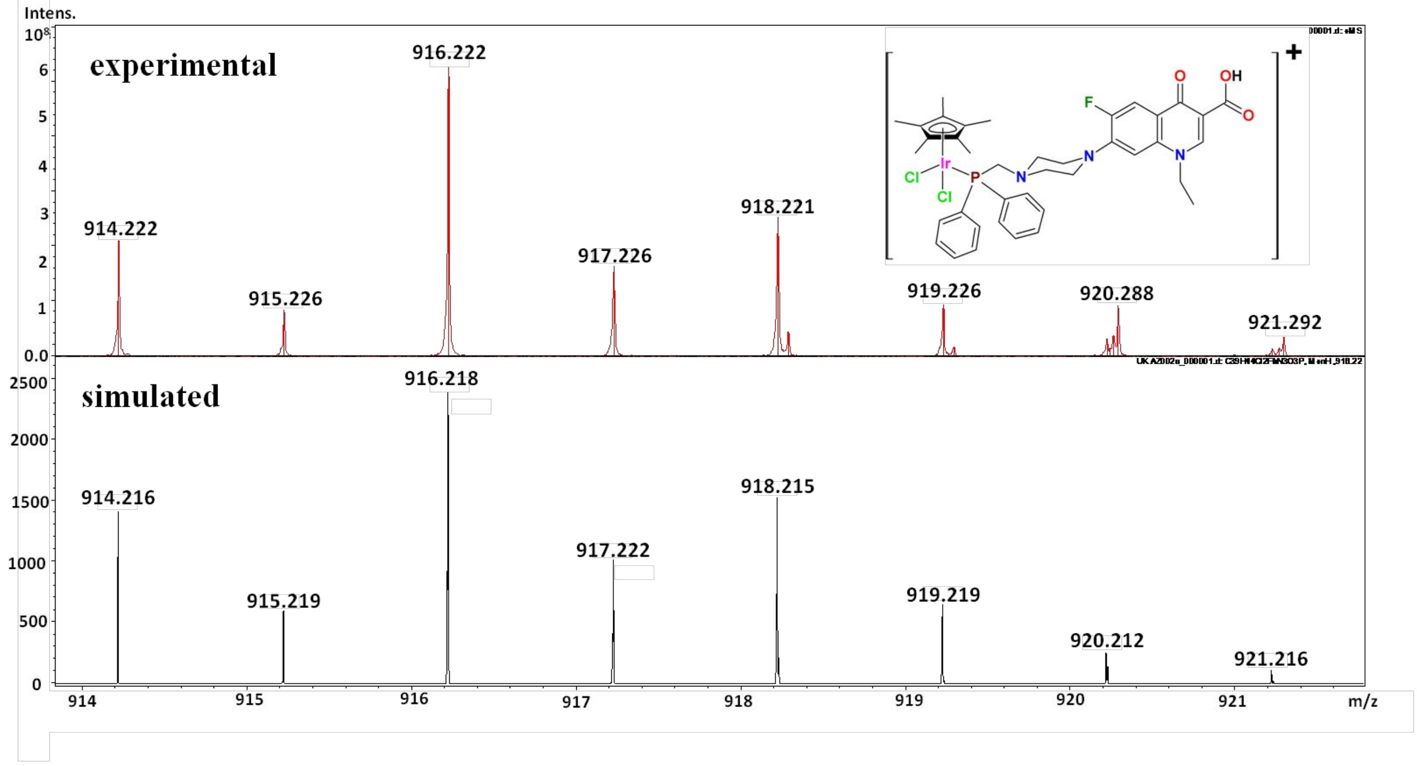

Fig. S9 (e)

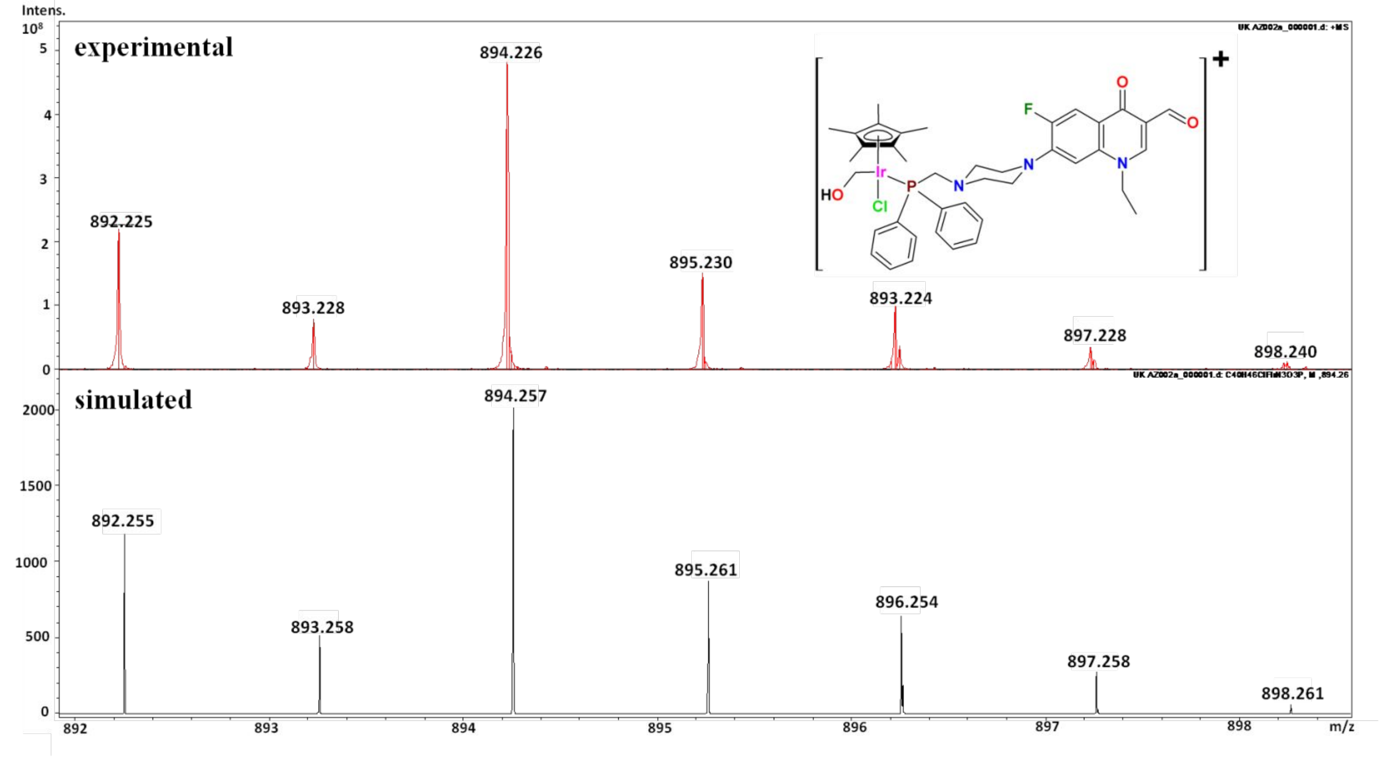

Fig. S9 (f)

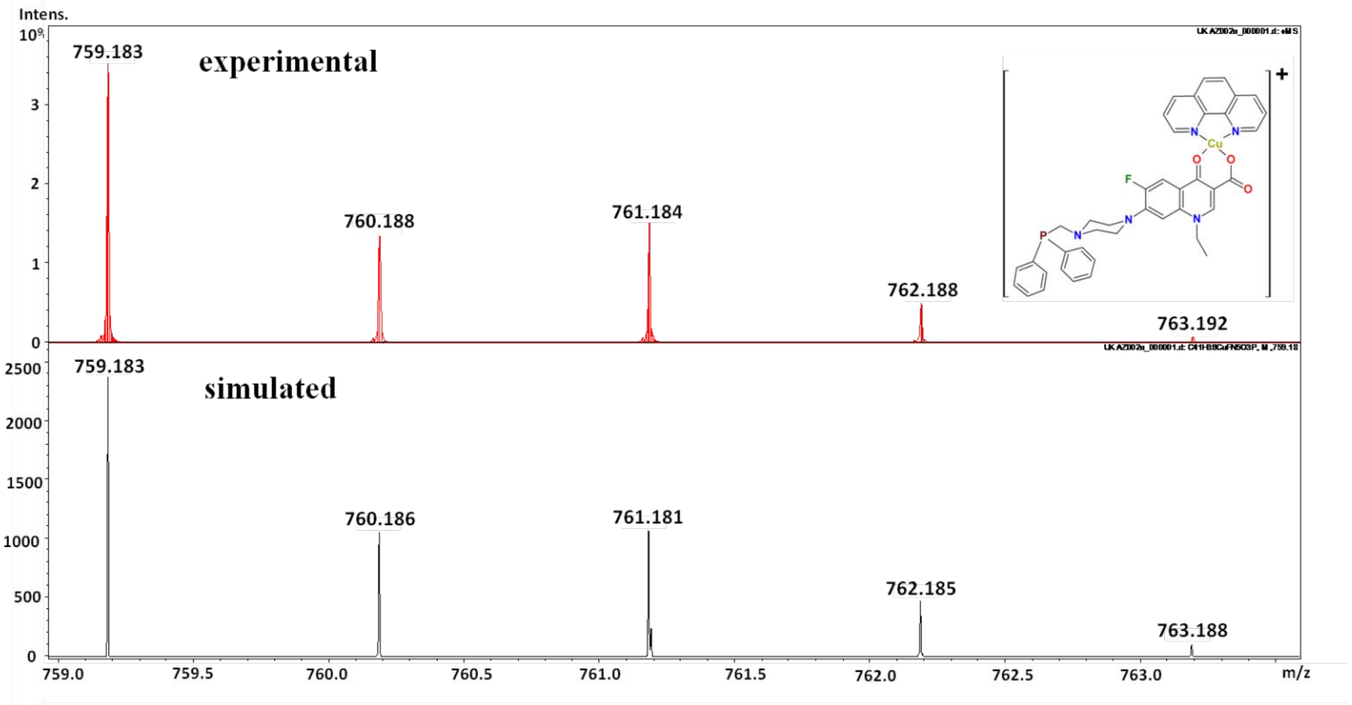

Figure S9 (a) ESI mass spectrum of **IrPNrCu(phen)**. ESI(+)MS in CH_3OH, m/z: 1157.199 [**M**]$^+$; 1123.246 [**M**-Cl+H]$^+$; 916.222 [**M**-Cu(phen)+H]$^+$; 894.226 [**M**-HCl-OH+CH_2OH]$^+$; 759.183 [**M**-IrCpCl_2]$^+$; **(b)** experimental and simulated spectra of [**M**]$^+$; **(c)** experimental and simulated spectra of [**M**-Cl+H]$^+$**(d)** experimental and simulated spectra of [**M**-Cu(phen)+H]$^+$**(e)** experimental and simulated spectra of [**M**-HCl-OH+CH_2OH]$^+$**(f)** experimental and simulated spectra of [**M**-IrCpCl_2]$^+$

Fig. S10 (a)

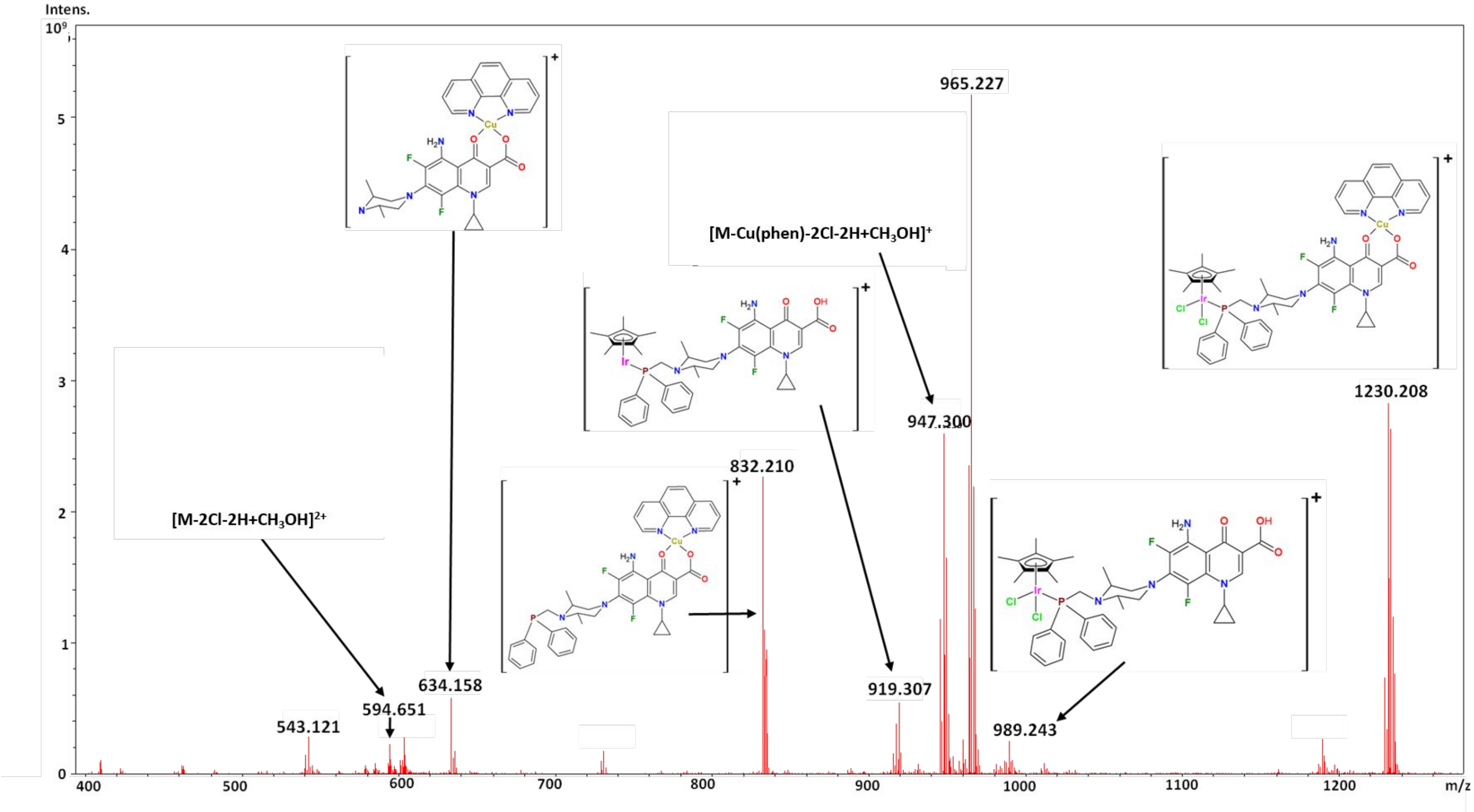

Intens.
10^9
[M-2Cl-2H+CH$_3$OH]$^{2+}$
[M-Cu(phen)-2Cl-2H+CH$_3$OH]$^+$
965.227
947.300
1230.208
832.210
634.158
919.307
594.651
543.121
989.243
m/z

Fig. S10 (b)

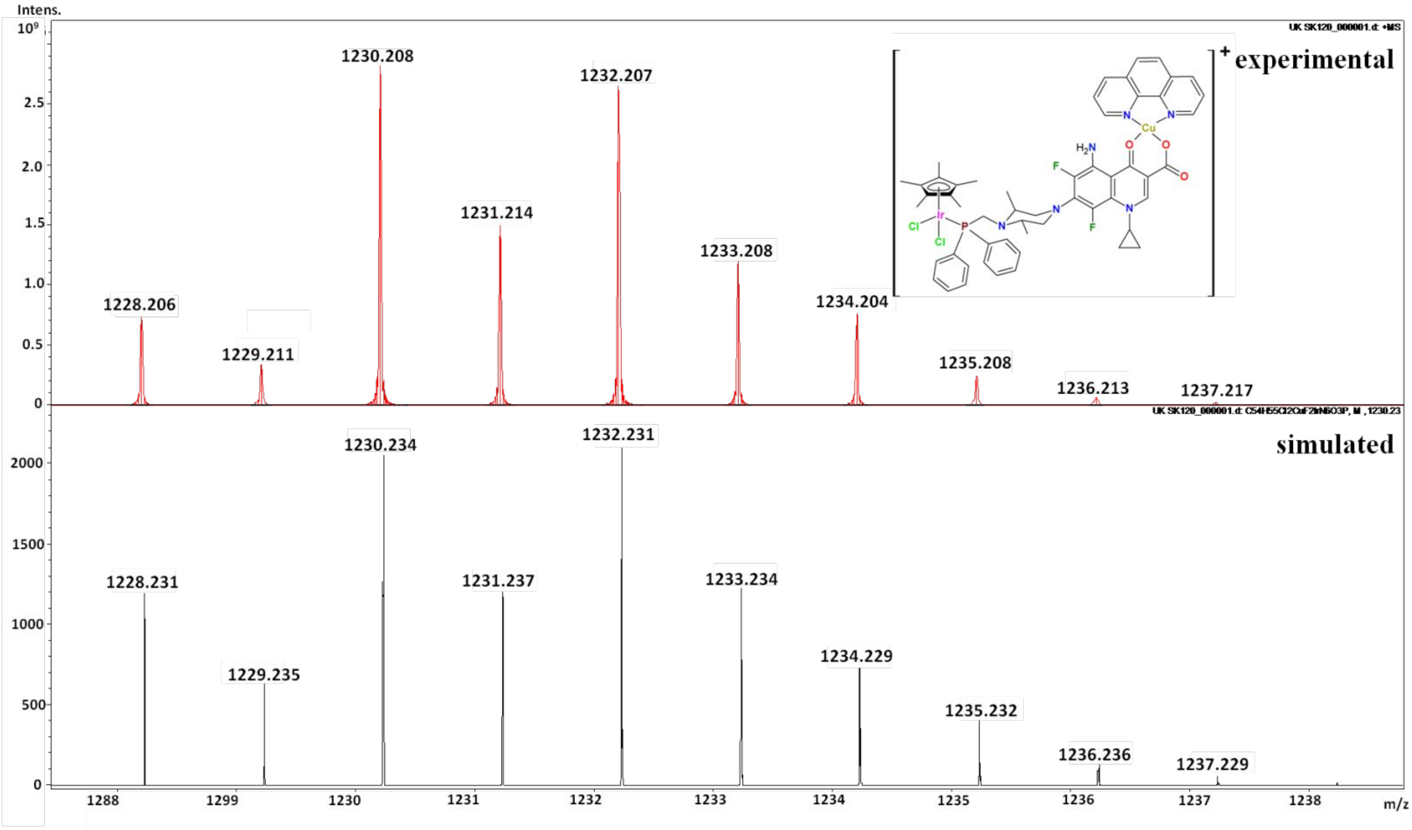

Fig. S10 (c)

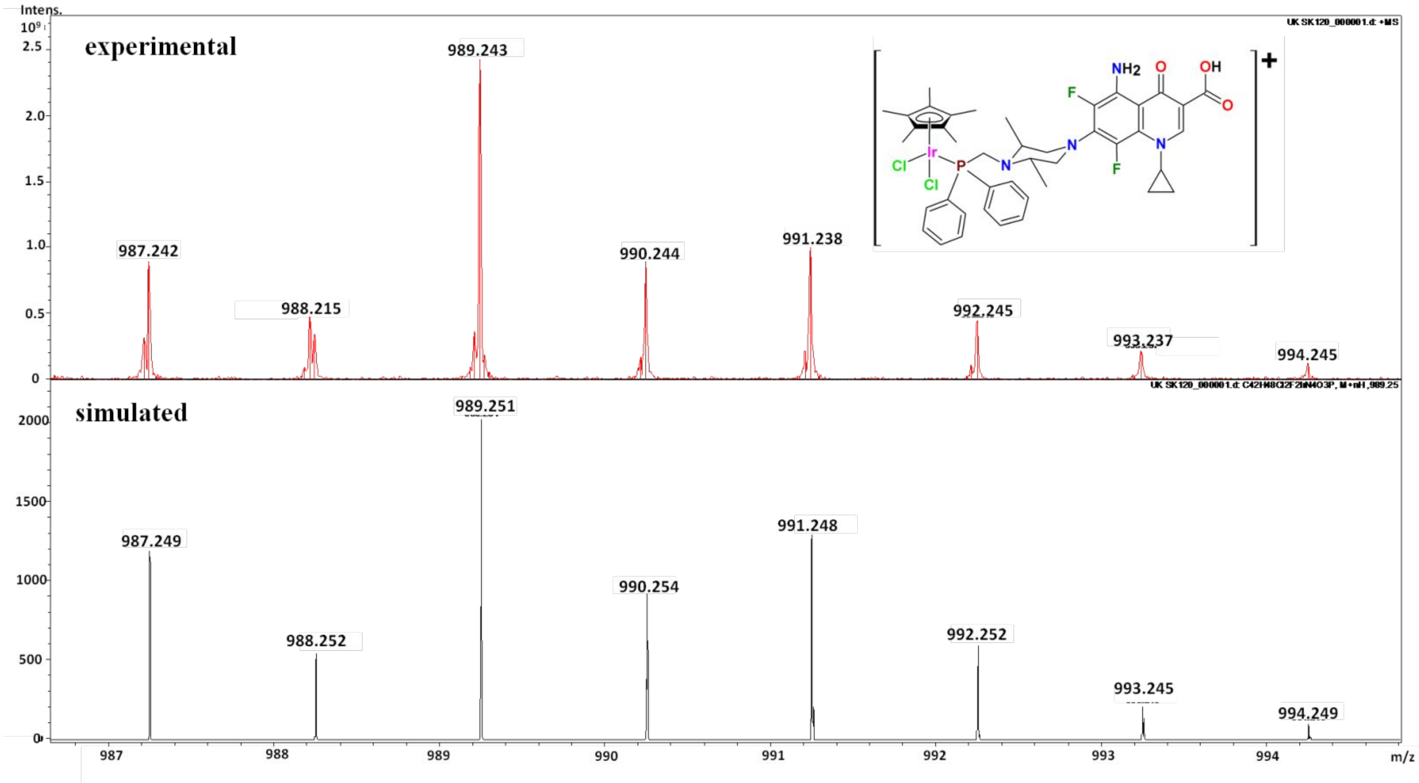

Fig. S10 (d)

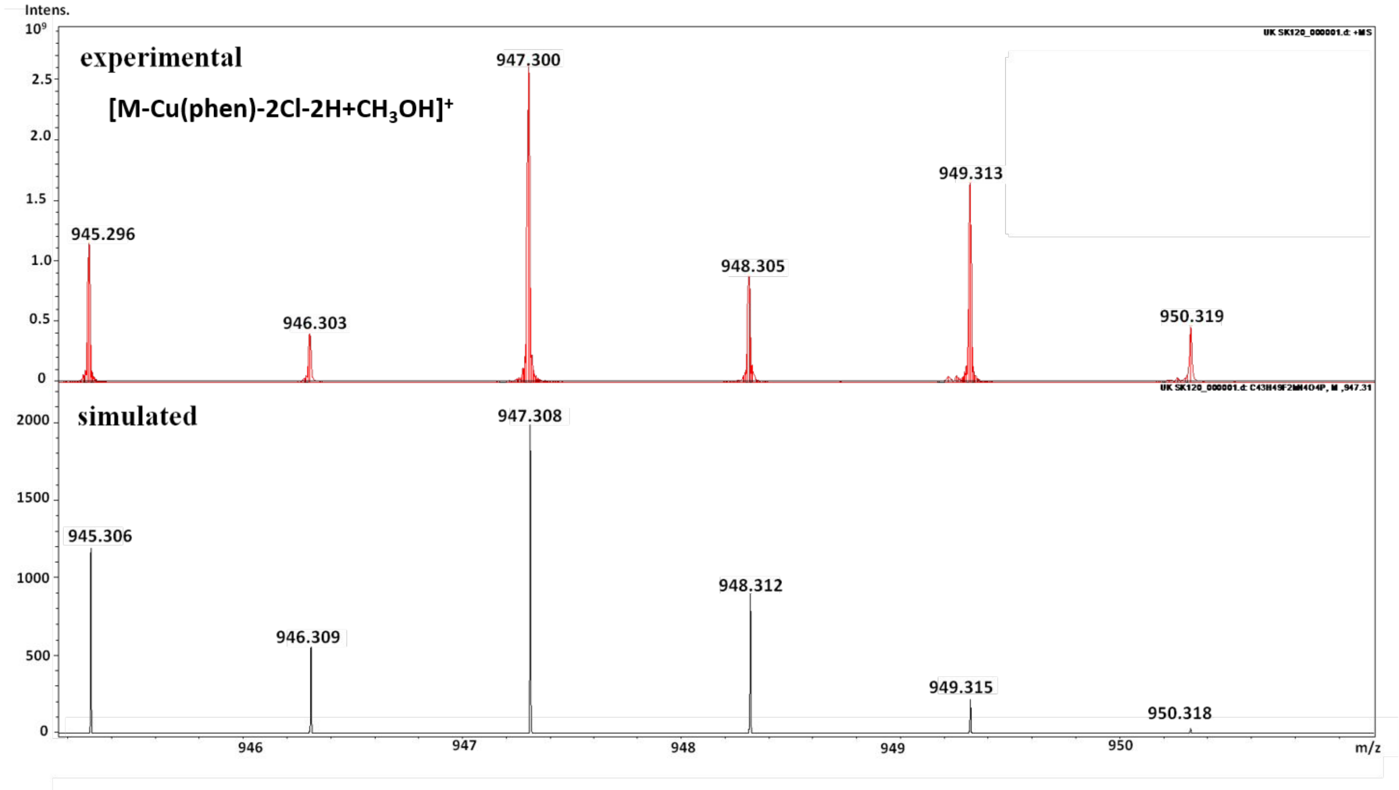

Fig. S10 (e)

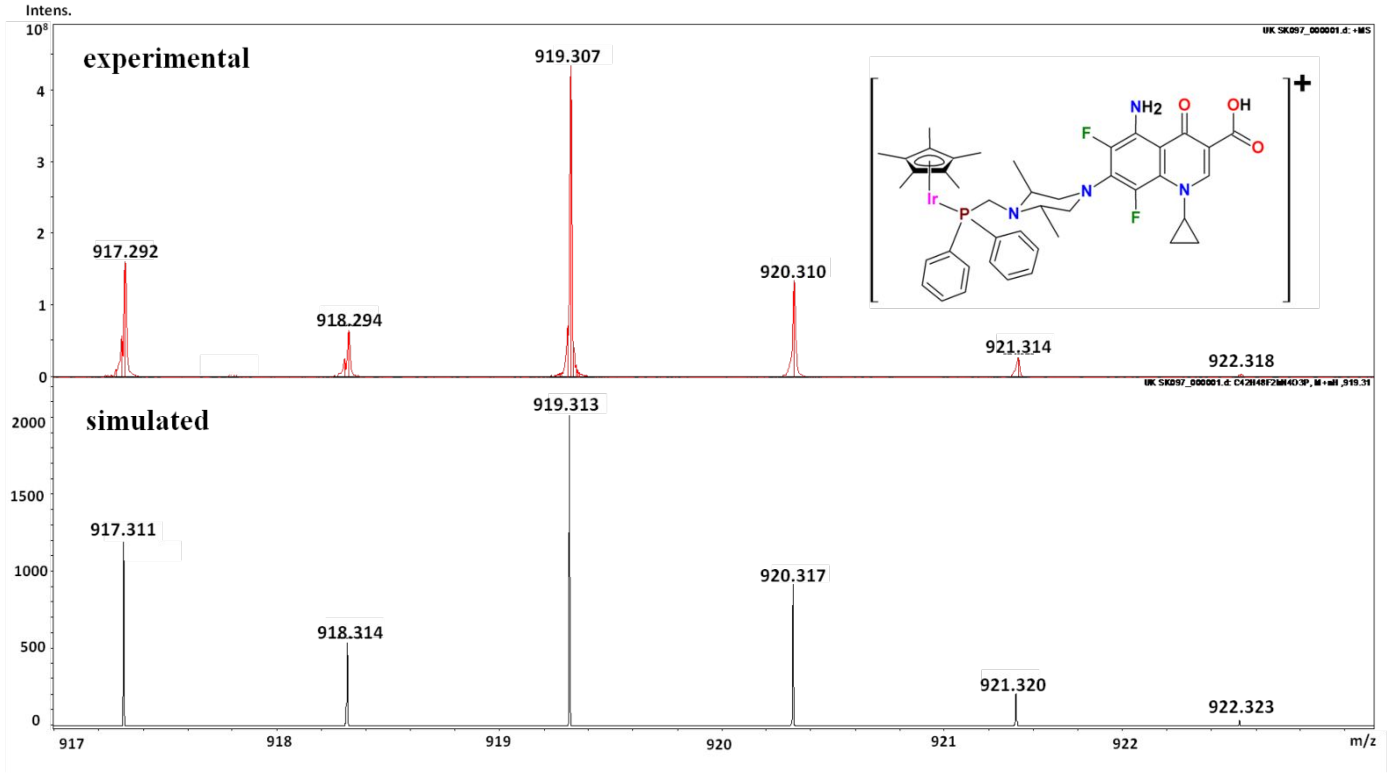

Fig. S10 (f)

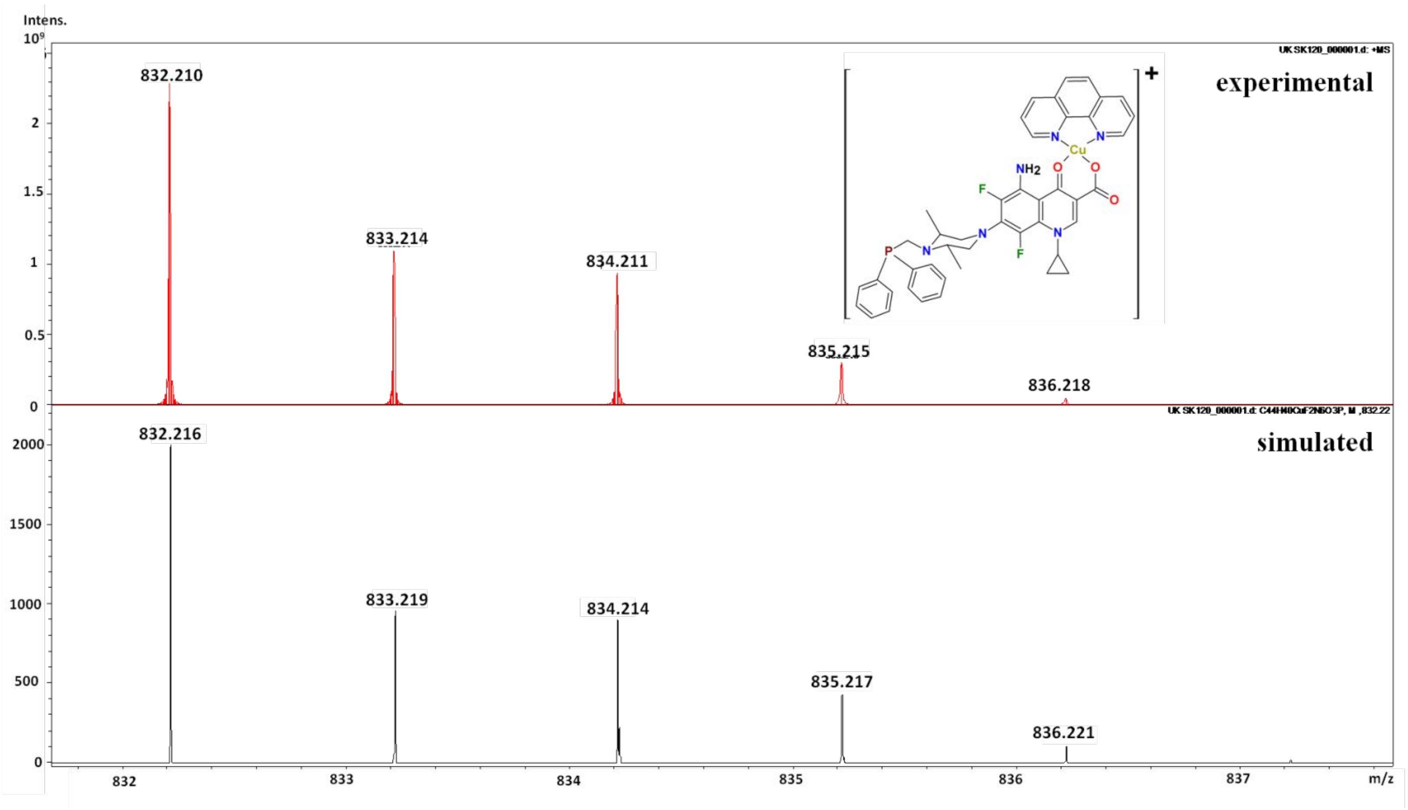

Fig. S10 (g)

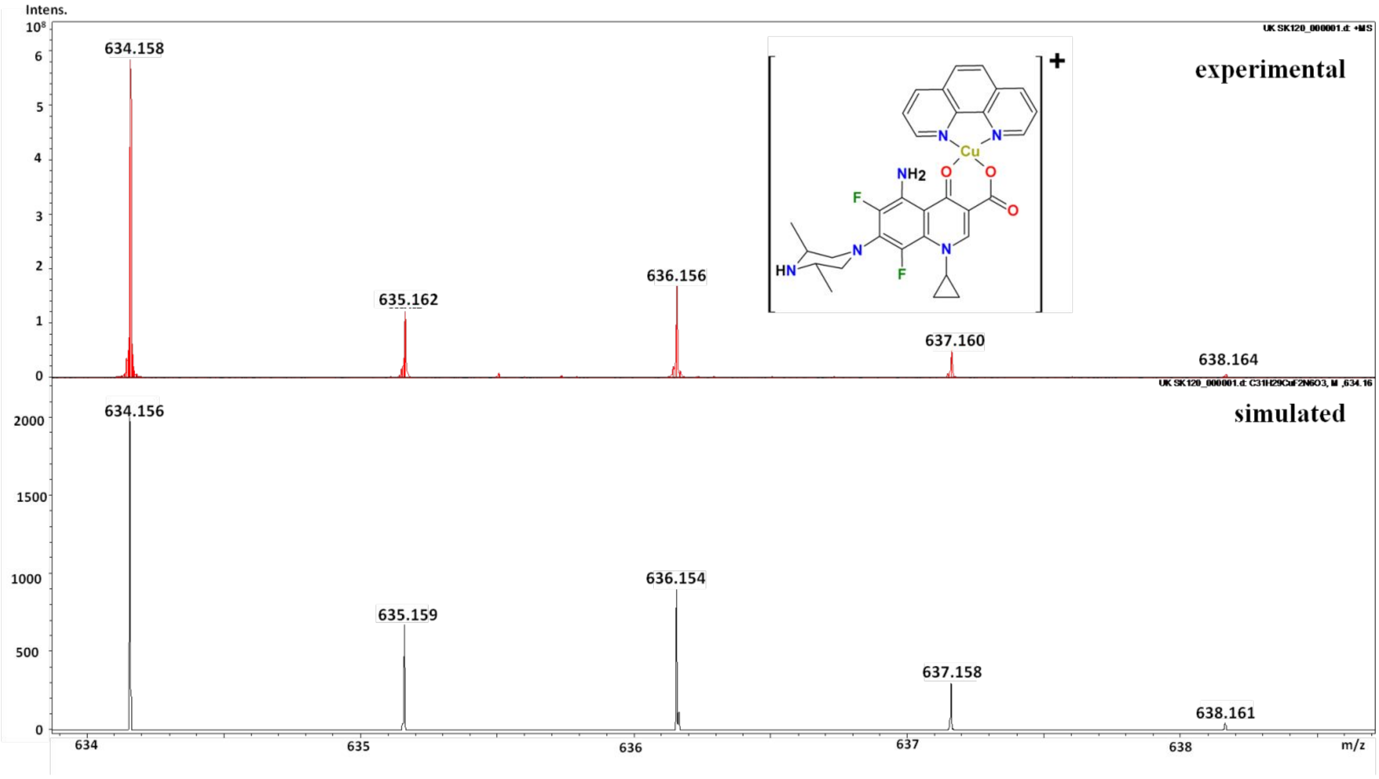

Fig. S10 (h)

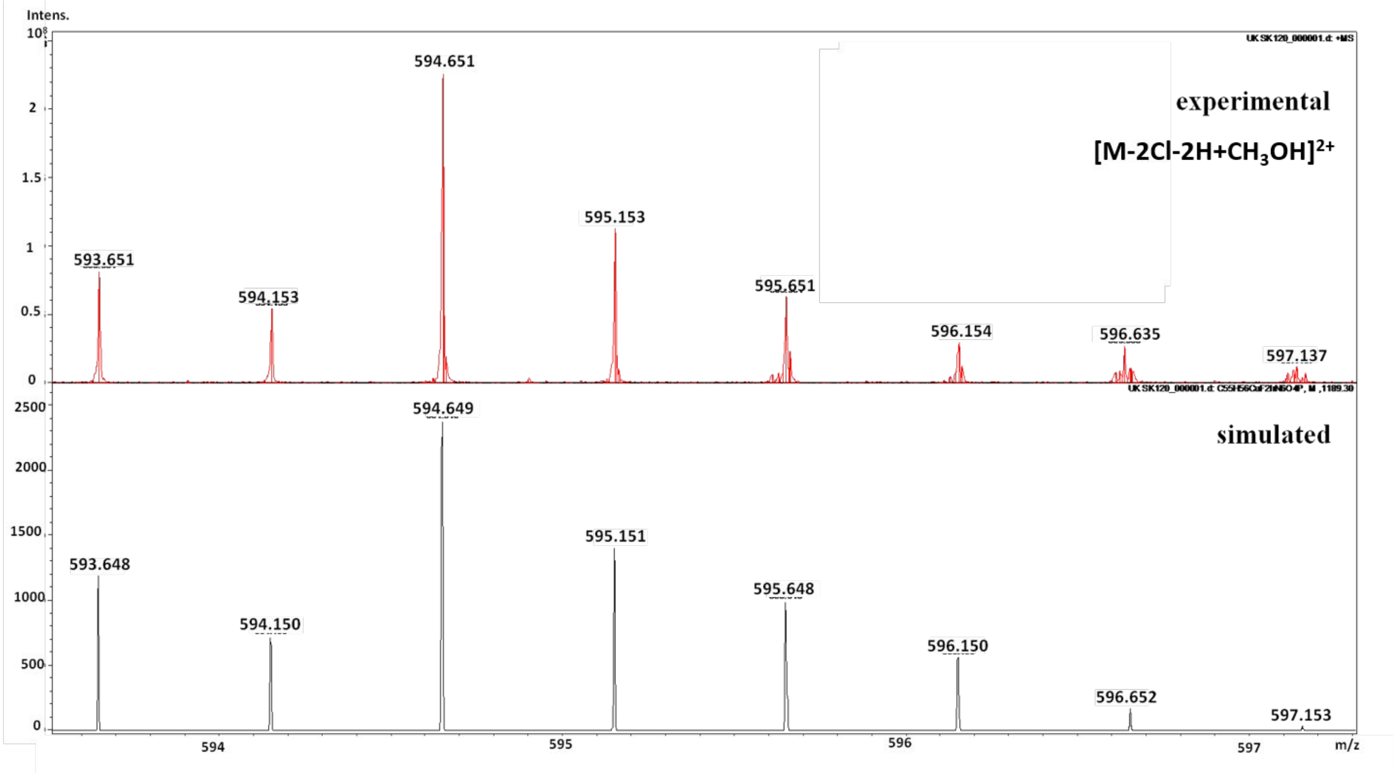

Figure S10 (a) ESI mass spectrum of **IrPSfCu(phen)**. ESI(+)MS in CH_3OH, m/z: 1230.208 [**M**]$^+$; 989.243 [**M**-Cu(phen)+H]$^+$; 947.300 [**M**-Cu(phen)-2HCl+CH_2OH]$^+$; 919.320 [**M**-Cu(phen)-2Cl+H]$^+$; 832.210 [**M**-IrCpCl_2]$^+$; 634.158 [**M**-IrCpCl_2-PPhCH_2]$^+$; 594.651 [**M**-2HCl+CH_2OH]$^{2+}$ (b) experimental and simulated spectra of [**M**]$^+$ (c) experimental and simulated spectra of [**M**-Cu(phen)+H]$^+$ (d) experimental and simulated spectra of [**M**-Cu(phen)-2HCl+CH_2OH]$^+$ (e) experimental and simulated spectra of [**M**-Cu(phen)-2Cl+H]$^+$ (f) experimental and simulated spectra of [**M**-IrCpCl_2]$^+$ (g) experimental and simulated spectra of [**M**-IrCpCl_2-PPhCH_2]$^+$ (h) experimental and simulated spectra of [**M**-2HCl+CH_2OH]$^+$

Infrared spectroscopies

The FT-IR spectra of the four novel iridium(III)-copper(II) complexes in the far infrared region are shown in in Fig. S10a, b for MIR region; and Fig. 10c shows FT-IR spectra of the starting reagent,

Cu(phen)$(NO_3)_2$: see the Supporting Information). The characteristic □(C-H) stretching vibrations generate in the spectral range of 3057-2853 cm^{-1} the medium peaks in the FT-ATR spectra of discussed complexes. In the free ligands a strong band near 1720 cm^{-1} is assigned to the □(C=O) stretching vibrations of their carboxylic group (-COOH),[1] which is very weak or not observed in ATR spectra of these complexes. Besides, in the FT-IR spectra of the novel complexes are observed two characteristic bands attributed to the antisymmetric and symmetric stretching vibrations of □(COO^-), which can be the marker of the coordination model. The bands with medium intensity around 1630 cm^{-1} and 1335 cm^{-1} are assigned to the $\nu_{as}(COO^-)$ and $\nu_s(COO^-)$ stretching vibrations, respectively (Tab. 3).

Table S2. The characteristic discussed bands in FT-IR spectra of complexes **1-4**.

Complex	ν_{as}(COO-) [cm^{-1}]	ν_s(COO-) [cm^{-1}]	Δν [cm^{-1}]	ν(C=O) [cm-1] pyridine group	ν (C=N) [cm^{-1}]	ν (Cu-O) [cm^{-1}]	ν (Cu-N) [cm^{-1}]
1	1618	1333	285	1583	1517	520	543
**1***	1630	1334	296	1587	1519	524	548
2	1627	1308	319	1584	1519	501	554
**2***	1632	1335	297	1587	1520	523	ov
3	1619	1321	298	1585	1520	512	523
**3***	1633	1327	306	1588	1522	512	540
4	1630	1292	338	1576	1519	ov	ov
**4***	1632	1293	339	1583	1520	ov	ov

Abbreviations: ν- stretching vibrations, ν_{as}- antisymmetric stretching vibrations, ν_s-symmetric stretching vibrations, ov- overlapped band; * in MeOH.

We have determined the model of binding the used ligands to Cu^{2+} by showing Δ parameter (Δ=($\nu_{as}(COO^-)$ $\nu_s(COO^-)$)). In the studied complexes Δ parameters are in the range of 285-339 cm^{-1}, which indicates the monodentate (unidentate) coordination of the carboxylate group in **1-4**, because Δ is larger than

in ionic compounds.[2] Nevertheless, the strong bands observed at 1624 cm^{-1}, 1628 cm^{-1}, 1612 cm^{-1} and at 1645 cm^{-1} in the FT-IR spectra of Ph_2PCH_2cfx, Ph_2PCH_2nfx, Ph_2PCH_2lfx, Ph_2PCH_2sfx phosphine ligands are assigned to ν(C=O)py stretching vibrations, respectively. These peaks are shifted to lower frequencies in the range of 1558-1568 cm^{-1} for the FT-IR spectra of studied iridium-copper complexes indicating decrease in the stretching force constant of the C=O bond as consequence of the coordination of the oxygen atom to the Cu^{2+} ions. In the FT-IR spectrum of free phen (1,10-phenanthroline) ligand a characteristic band around 1586 cm^{-1} is due to ν(C=N) stretching vibrations and is shifted to a lower frequency in FT-IR spectra of **1-4** (ν=69-64 cm^{-1}). This shift indicates the coordination of the pyridine nitrogen atoms of phen to the cooper ions (from nitrogen atom to the empty d-orbital of the metal ion) in a bidentate model.[2]

Actually, the FT-IR spectroscopy can be used as a good analytical tool to follow the coordination of organic ligands with metal ions,[3] which we confirm by analysis in FIR spectral region of the studied complexes **1-4**. The coordination marker bands: ν(Cu-O) and ν(Cu-N) are found in the FT-IR spectra of these complexes, *i.e.* at 520, and 543 cm^{-1} for complex **1**, at 554 cm^{-1} and 501 cm^{-1} for complex **2**, respectively. These bands are absent in the spectra of free Ph_2PCH_2cfx, Ph_2PCH_2nfx, Ph_2PCH_2lfx, Ph_2PCH_2sfx ligands and phen. Nevertheless, dissolving the obtained complexes in methanol hardly changes their structures, which can be seen in the comparison of the spectra in the solid state with methanol solutions (Fig. S10a,b see the Supporting Information).

1. Nakamoto, K. Infrared and Raman Spectra of Inorganic and Coordination Compounds, Part B. *J. Wiley & Sons Inc.* New York, **2009**

2. Socrates, G. Infrared and Raman characteristic group frequencies: tables and charts. *J. Wiley & Sons*, **2004**

3. Yadav, S.; Singh, R. V. Ferrocenyl-substituted Schiff base complexes of boron: Synthesis,structural, physico-chemical and biochemical aspects. *Spectrochim Acta A Mol Biomol Spectrosc* **2011**, *78*, 298-306

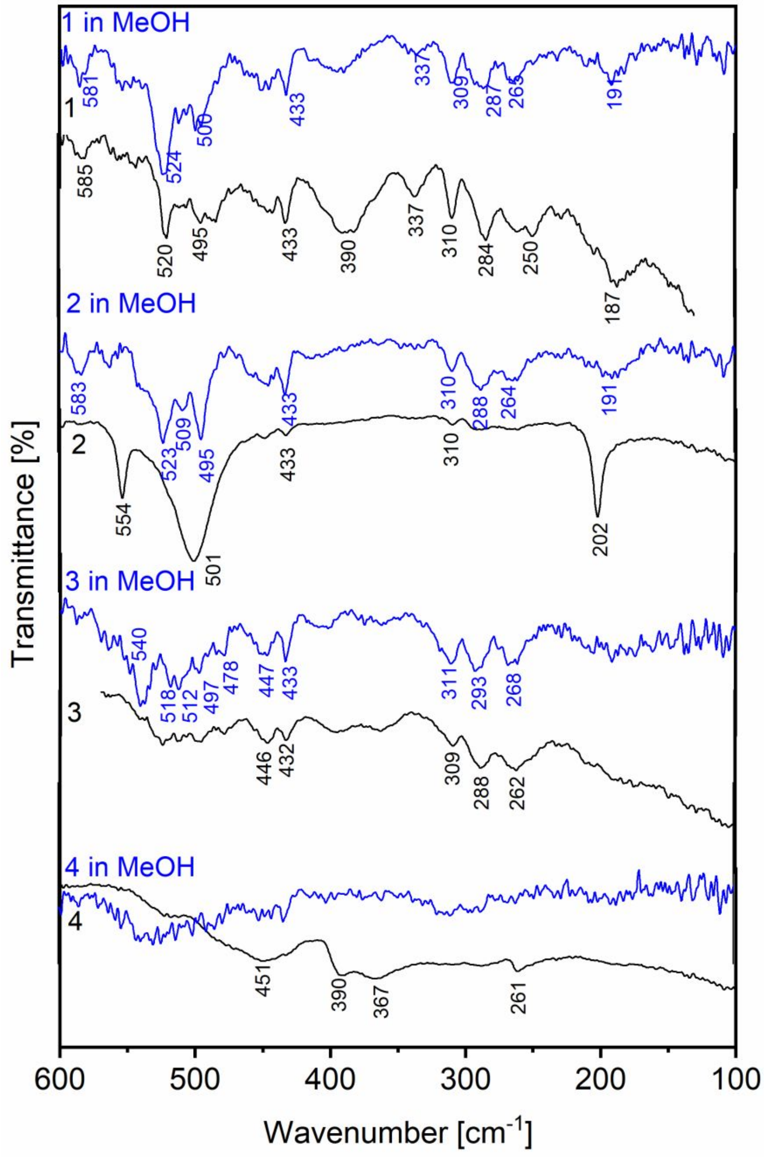

Figure S11 a The FT-FIR spectra of complexes **1-4**.

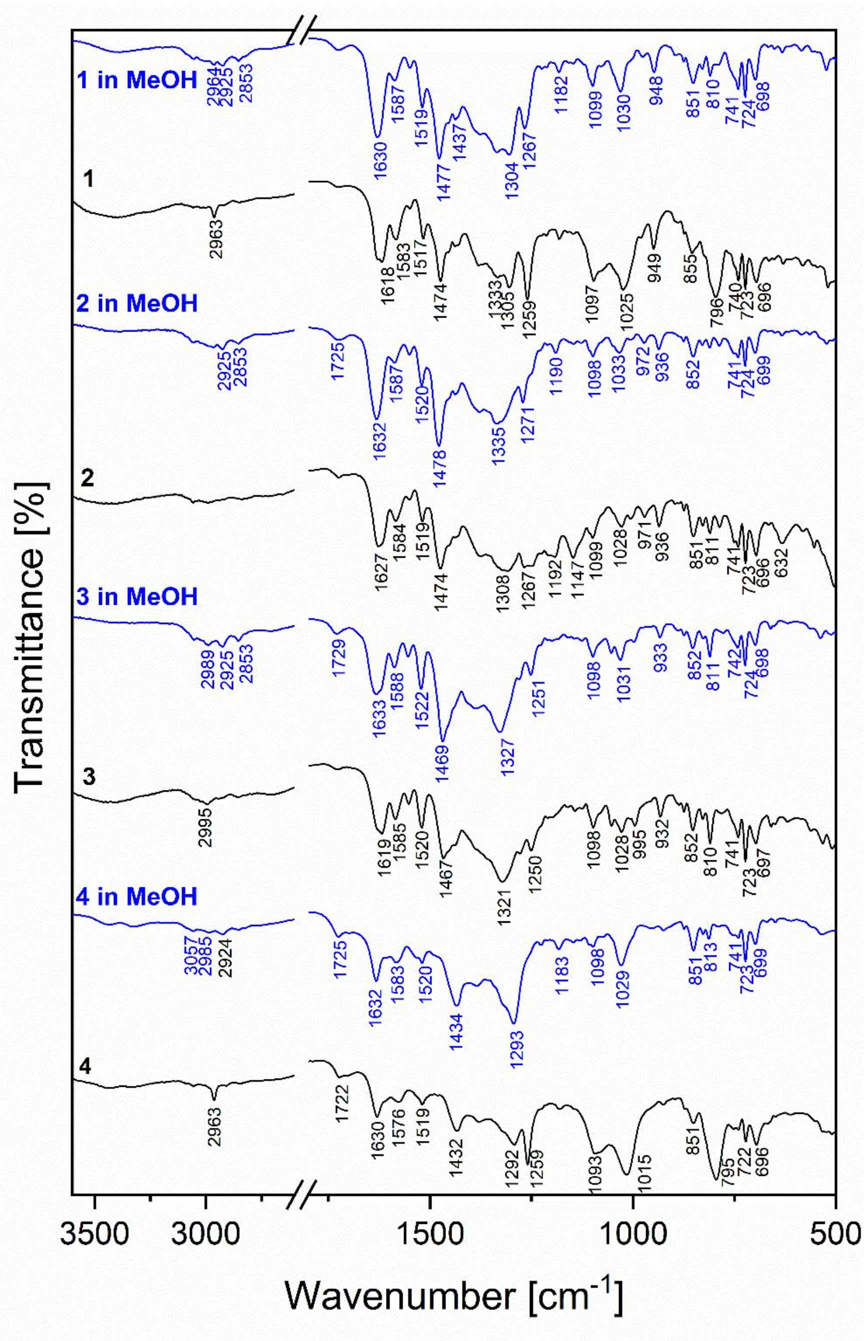

Figure S11 b The FT-IR spectra of complexes 1-4 in the MIR spectral region.

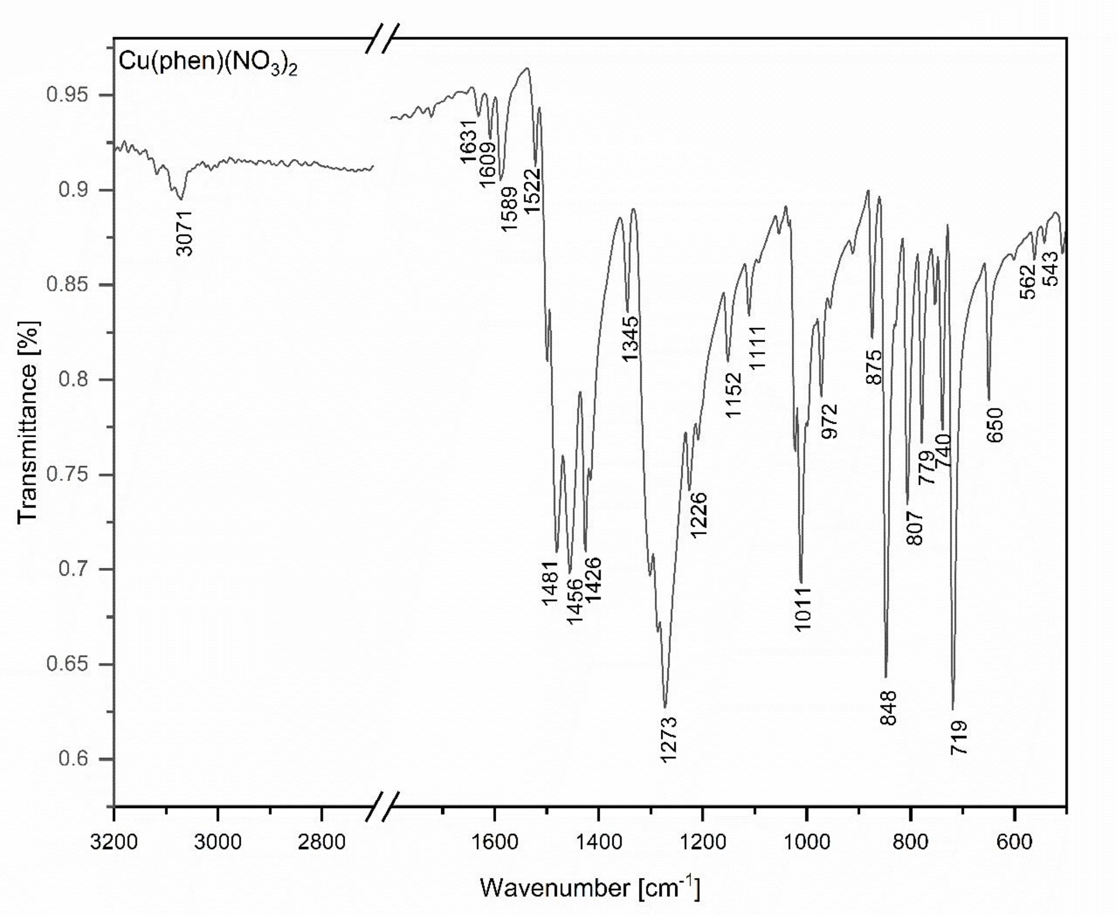

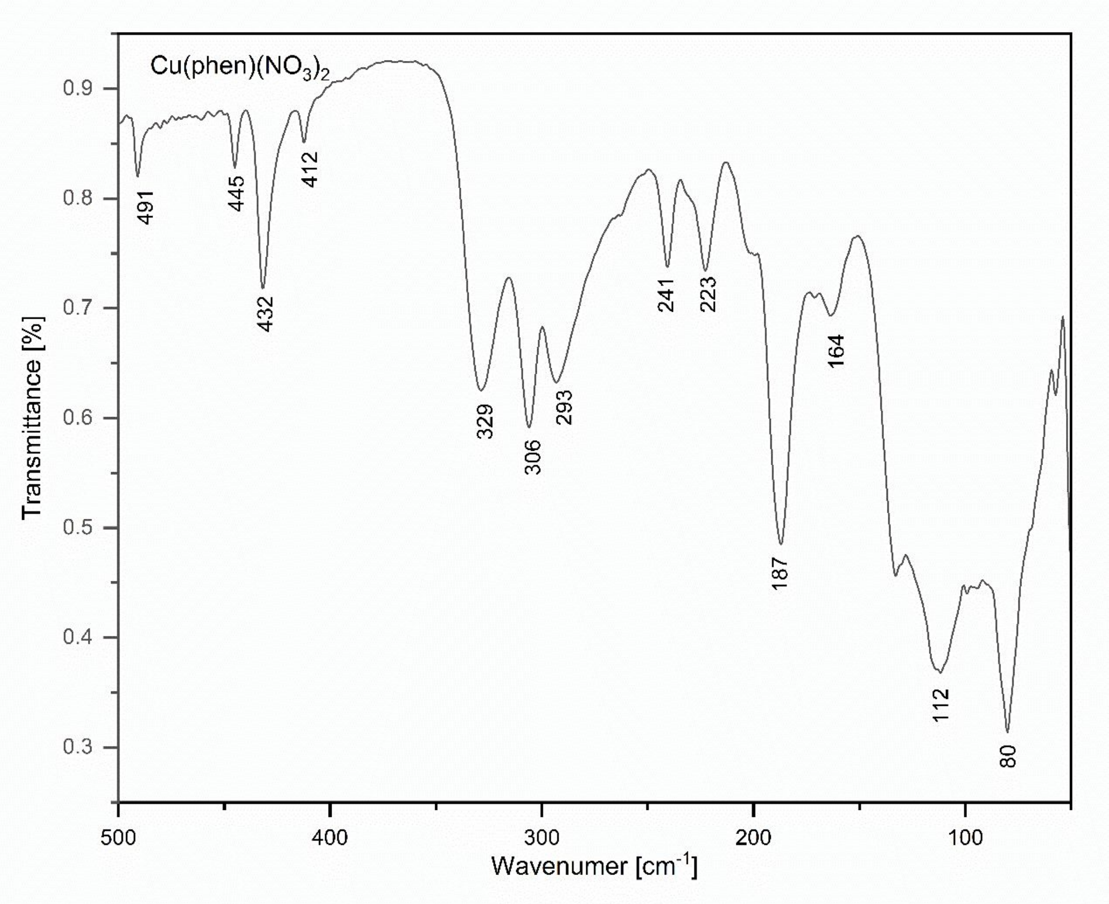

Figure S11 c The FT-IR spectra of Cu(phen)(NO_3)$_2$ in the MIR and FIR spectral regions.

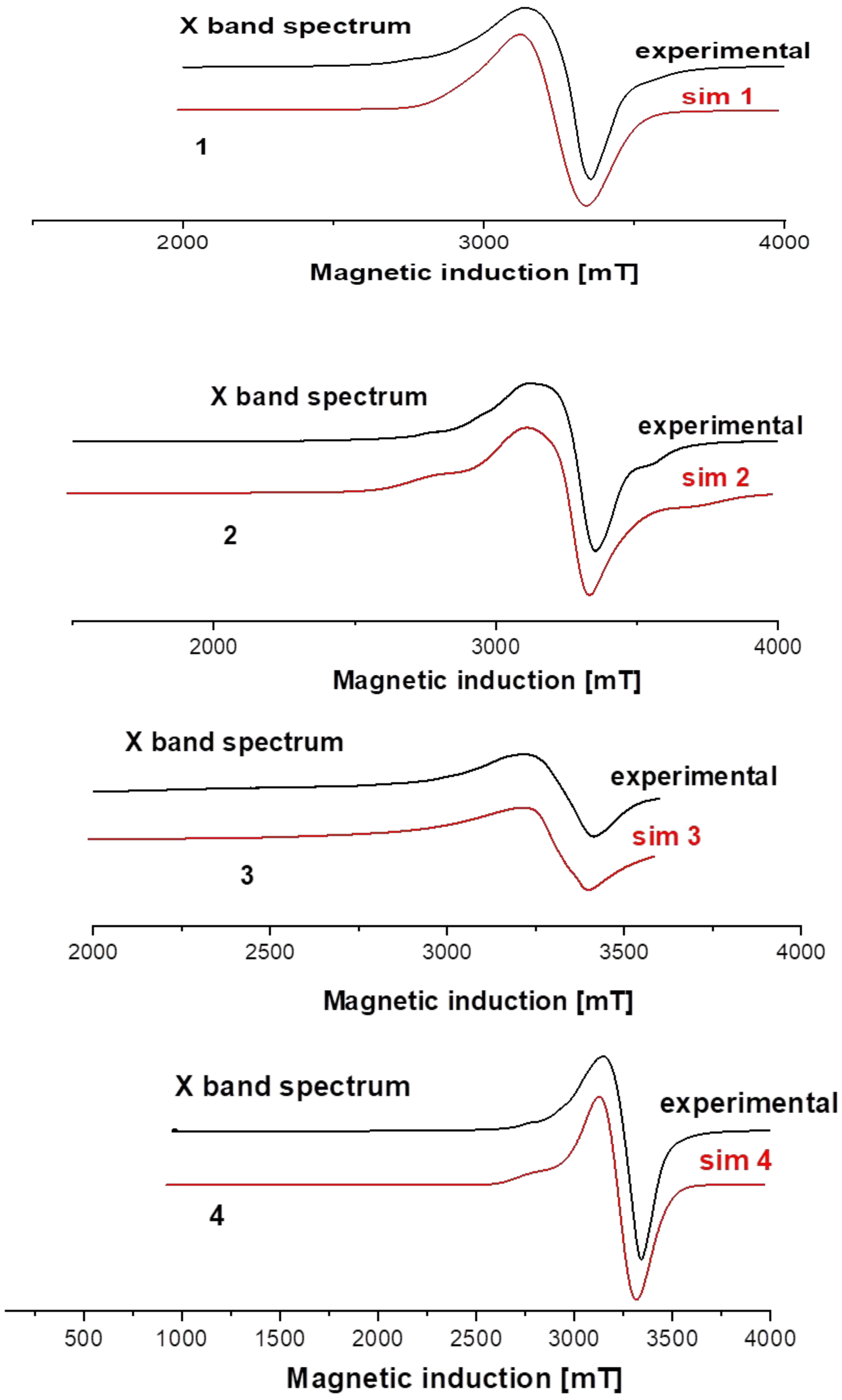

Figure S12 The X-band EPR spectra of **1 - 4** at 77 K together with the spectrum calculated by computer simulation of the experimental spectra with spin Hamiltonian parameters given in the text.

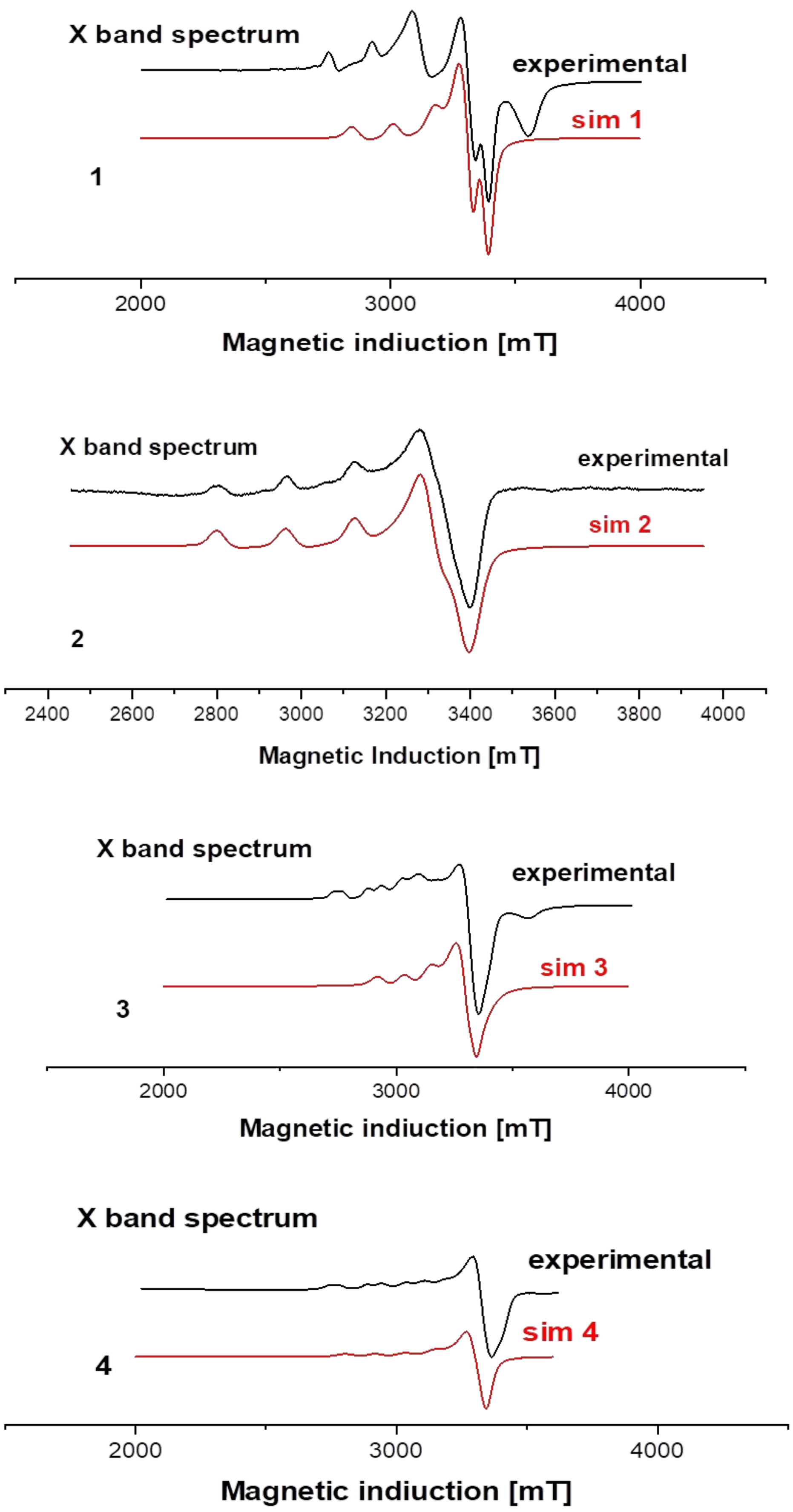

Figure S13 EPR frozen solution spectra (at 77 K) of compounds **1 - 4**; in DMSO solvent together with the theoretical spectrum calculated using the parameters given in the text.

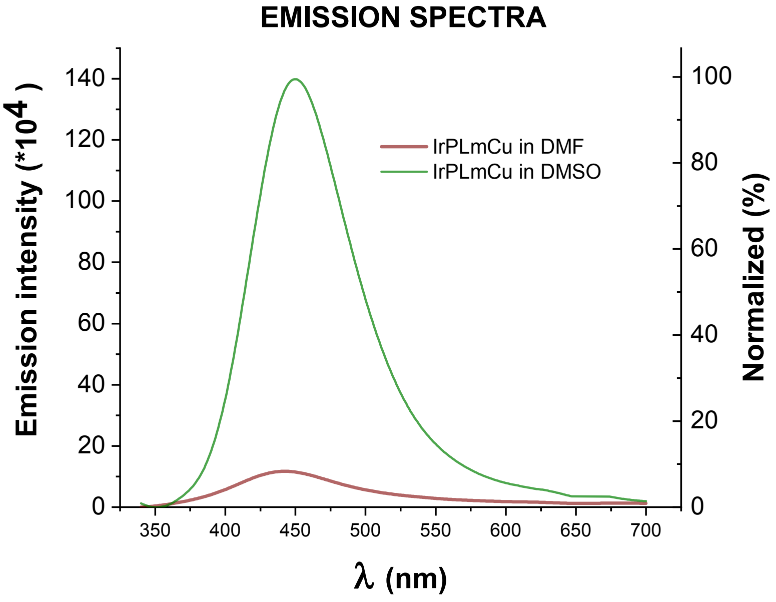

Figure S14 Emission spectra obtained for IrPLmCu(Phen) and RuPLmCu(Phen) both in DMF and DMSO solution.

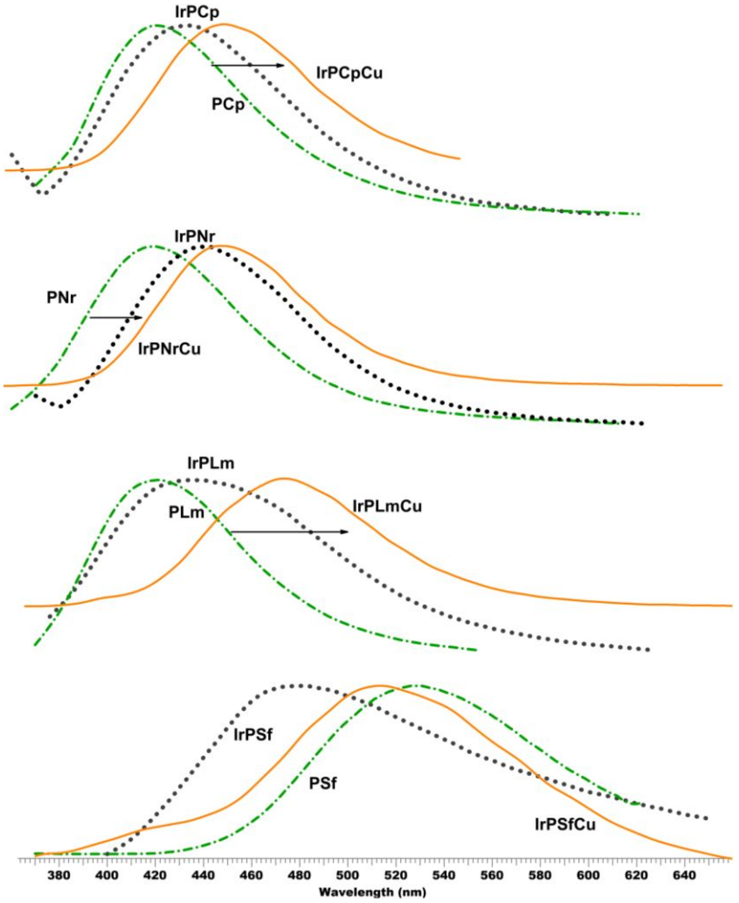

Figure S15 Normalized emission spectra for heteronuclear Ir(III)/Cu(II) complexes, homonuclear Ir(III) complexes and the corresponding phosphine ligands; λex = 340 nm, 298 K.

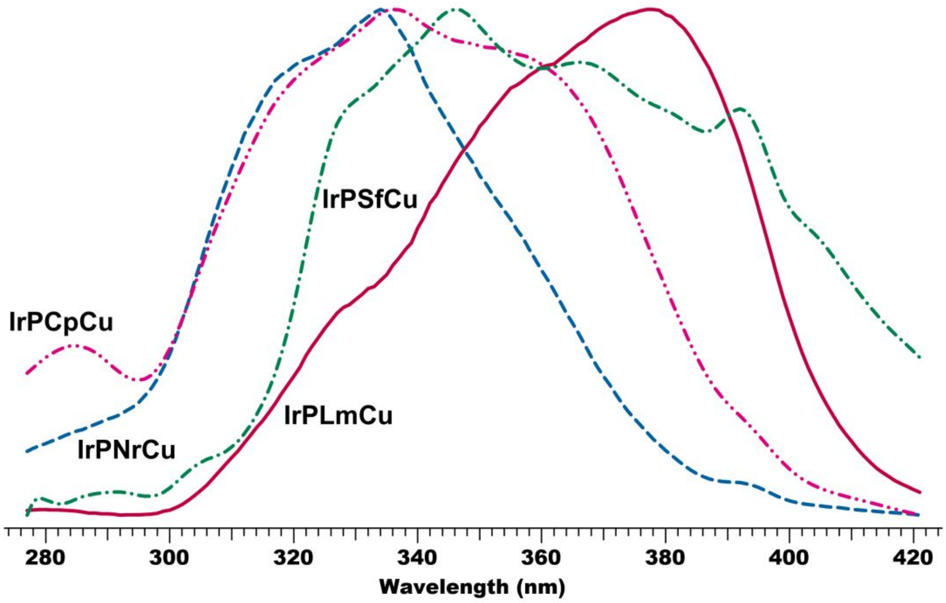

Figure S16 Excitation spectra of IrPCpCu, IrPNrCu, IrPLmCu and IrPSfCu

Magneto-structural studies

DC measurements

To fit and interpret the magnetic susceptibility data of examined complexes, first it is necessary to find all possible magnetic pathways. As mentioned in the structural discussion, complexes **3** can be viewed as a monometallic chain in which the neighboring Cu(II) ions are bridged either by carboxylate groups of phosphino-fluoroquinolone ligands or OH- linkers with Cu···Cu separation of 3.470 Å and 4.193 Å, respectively. Considering above the susceptibility data was analysed using a Hamiltonian [eq. 1] for an alternating Ising chain shows in scheme S1: (52)

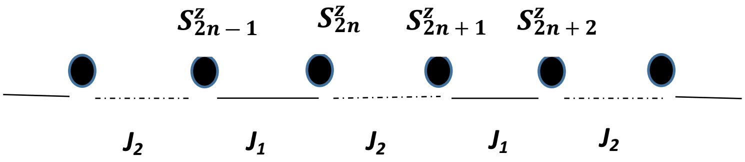

Scheme S1 Alternating Ising chain.

$$H = \sum_{n=1}^{N/2}\left[-2J_1 S^z_{2n-1}S^z_{2n} - 2J_2 S^z_{2n}S^z_{2n+1} - g\beta H\left(S^z_{2n-1} - S^z_{2n}\right)\right], \qquad \textbf{eq. 1}$$

where S^z_{2n} denotes the z – component of the $2^{n\text{-th}}$ spin in a linear chain, J_1 and J_2 are

The zero-field susceptibility of alternating antiferromagnetic Ising chain is:

$$\chi = \frac{Ng^2\beta^2}{4kT}\left[\frac{e^{K_1+K_2}}{cosh(K_1-K_2)}\right], \qquad \textbf{eq. 2}$$

Where $K_1 = \frac{J_1}{2kT}$, $K_2 = \frac{J_2}{2kT}$.

The best agreement with the experimental magnetic data for 3 was obtained with J1 = –0.82 cm–1, J2 = –0.29 cm–1, gav(Cu) = 2.09 and TIP = 66·10–6 cm3mol–1, R = Σ[(χT)exp – (χT)calc]2/ Σ[(χT)exp]2 = 2.42·10–5 (solid blue line in Figure 7A). The calculated curve matches the magnetic data well. The obtained

result suggests that the stronger antiferromagnetic exchange interaction is mediated through the doubly oxygen bridge of carboxylate group (the higher J value, shorter Cu···Cu contact) which correspond to the magneto-structural correlations presented in the literature. (53,54) For dimers with planar or near-planar cores $[Cu2(\mu2\text{-}O)2]_2^+$, the exchange coupling constant (J) depends on the value of the Cu - O - Cu (φ) angle and the Cu-O bridge distance (R) especially expressed by the φ / R ratio, and from more complicated factors such as geometry around paramagnetic centers. (55-57) Thus for φ ˃ 97.5o the interaction is predicted to be antiferromagnetic (S = 0 ground state) and for φ ˂ 97.5o the ground state is equal 1 (S = 1) and interaction should be ferromagnetic. The small magnitude of this interaction may be due to the asymmetry of the oxide bridge (two different R values, one much shorter 2.058 Å and the other much longer 2.220 Å) causing distortion of the OH geometry and an unusually long distance Cu ··· Cu.

Complexes **1**, **2** and **4** can be considered as mononuclear species which create supramolecular one-dimensional polymeric architecture through π-stacking interactions and system of hydrogen bonds between the water molecule and oxygen atoms from carbonyl groups with shortest Cu ··· Cu contact 4.196 (1), 4.904 (2) Å. The intermolecular interactions in all monomeric complexes was calculated using the well-known PHI program (51) which allows the simultaneous fitting of $\chi T(T)$ and M(H) dependencies. The TIP was also included into the fitting procedure. The least squares fit of the experimental data by this expressions leads to the following results:, $g_{av}(Cu) = 2.09$, $zJ' = -0.09$ cm^{-1}, TIP = 138×10^{-6} for **1** (red lines in Fig. 7A) and $g_{av}(Cu) = 2.11$, $zJ' = 0.15$ cm^{-1}, TIP = 192×10^{-6} for **2** (green lines in Fig. 7A) and $g_{av}(Cu) = 2.14$, $zJ' = 0.28$ cm^{-1}, TIP = 226×10^{-6} for **4** (black lines in Fig. 7A). The discrepancy factor is 2.09×10^{-5} (**1**), 6.93×10^{-6} (**2**) and 3.57×10^{-6} (**4**). These data indicate that a weak exchange interaction between nearest copper atoms in the crystal lattices can exist of antiferromagnetic in **1** and ferromagnetic in **2** and **4** nature. The temperature-independent paramagnetic term is bigger than usually found. Although the origins of the observed phenomenon are unclear, it was verified by repeated measurements.

AC susceptibility measurements

New information was obtained from the AC susceptibility measurements. They were performed first at low temperature T = 2.0 K for a set of representative frequencies of the alternating field (f = 1.1, 11, 111, and 1111 Hz) by ramping the magnetic field from zero to BDC = 1 T with the working amplitude BAC = 0.3 μT. There was no absorption signal (out-of-phase susceptibility component χ'') at the zero-field owing to a fast magnetic tunneling. With the increasing external field, this component raised, and only for complex **2** passed through a maximum between 0.1 and 0.2 T at the highest frequencies (Figure S16). This behavior indicates that **2** can exhibit the field induced slow magnetic relaxation.

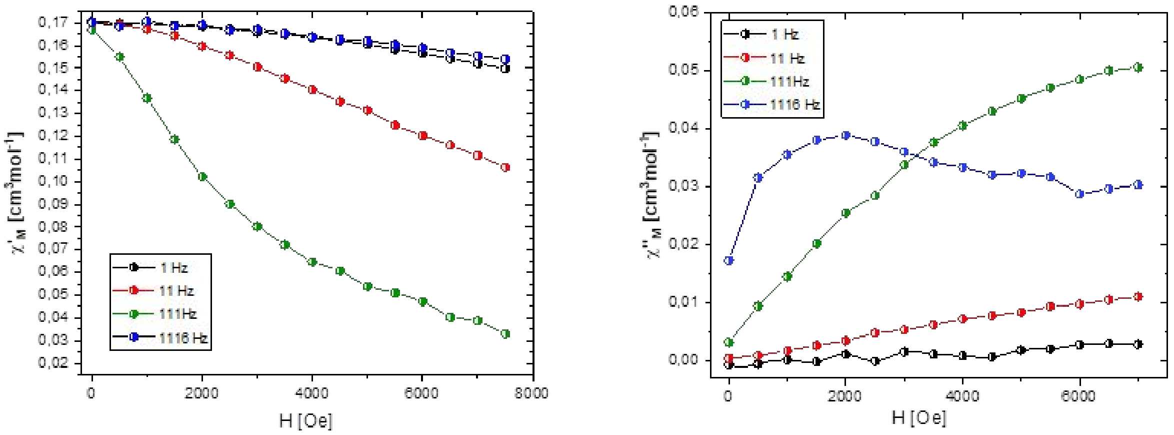

Figure S17 Field dependencies of the AC susceptibility components for 2 at T = 2.0 K for a set of frequencies of the AC field. Lines are a guide for the eye.

Subsequent experiments were done at a fixed external magnetic field BDC = 0.1 T (the maximum of the high-frequency signal) changing the frequency between f = 0.1 to 1500 Hz for a set of temperatures between T = 1.8 and 12 K (Figure S17).

The in-phase (χM') and out-of-phase (χM'') components exhibit small frequency dependences with the application of an external field of 0.2 T, indicative the possibility of a slow relaxation of magnetization, although the maxima in χM'' are missing. Using this data we cannot suggest SMM or SIM behavior. However, the relaxation process for Cu(II) ions is very rare due to the absence of a barrier to spin reversal: the axial zero-field splitting parameter D is undefined. However, some S = 1/2 spin systems such V(IV),

low-spin Mn(IV), Ni(I, III), only three example of Cu(II), Os(V), Ir(IV), Fe(III) and Ru(III) complexes show a slow magnetic relaxation (SMR) that is supported by the external magnetic field. (58-62) The presence of a relaxation process in complex **2** can be a result of geometry around Cu(II) ions. Though the D parameter cannot be assigned to mononuclear copper(II) complexes, these are well-known as anisotropic systems showing at least two distinct $g_z \neq g_x$ values well seen in the EPR spectra of an axial type. Thus, even in the absence of the zero-field splitting, there exists a magnetic anisotropy. Additionally, the rest complexes not exhibit a relaxation process. This may be due to the presence of higher square-pyramidal geometry distortion in **2** (τ' = 0.26), which should lead to a greater difference between gx and gy and thus greater anisotropy of g tensor than those for **1**, **3** and **4**.

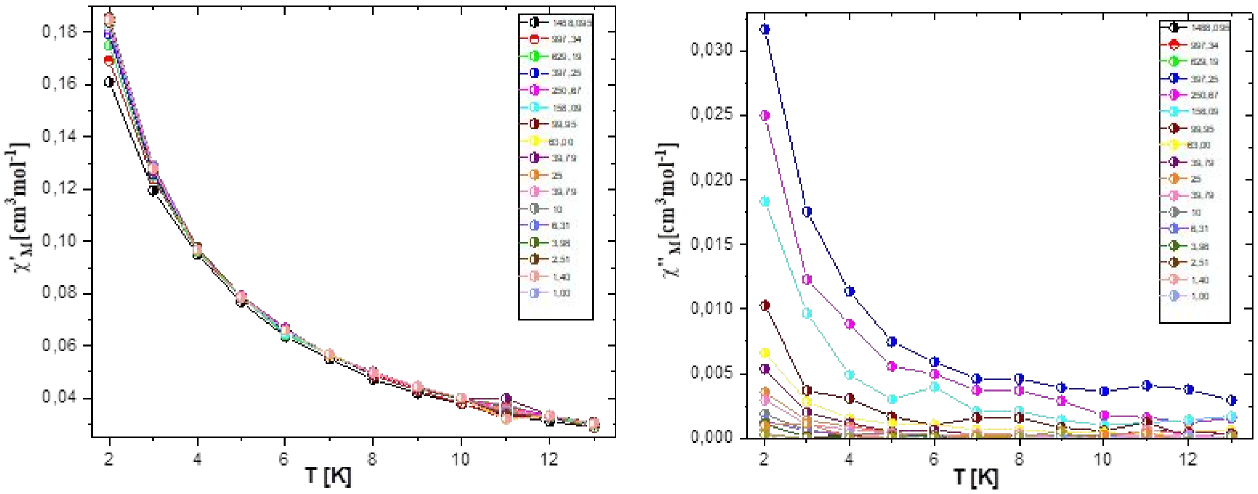

Figure S18 Temperature dependence of (a) the in-phase and (b) out-of-phase molar susceptibility of **2**.

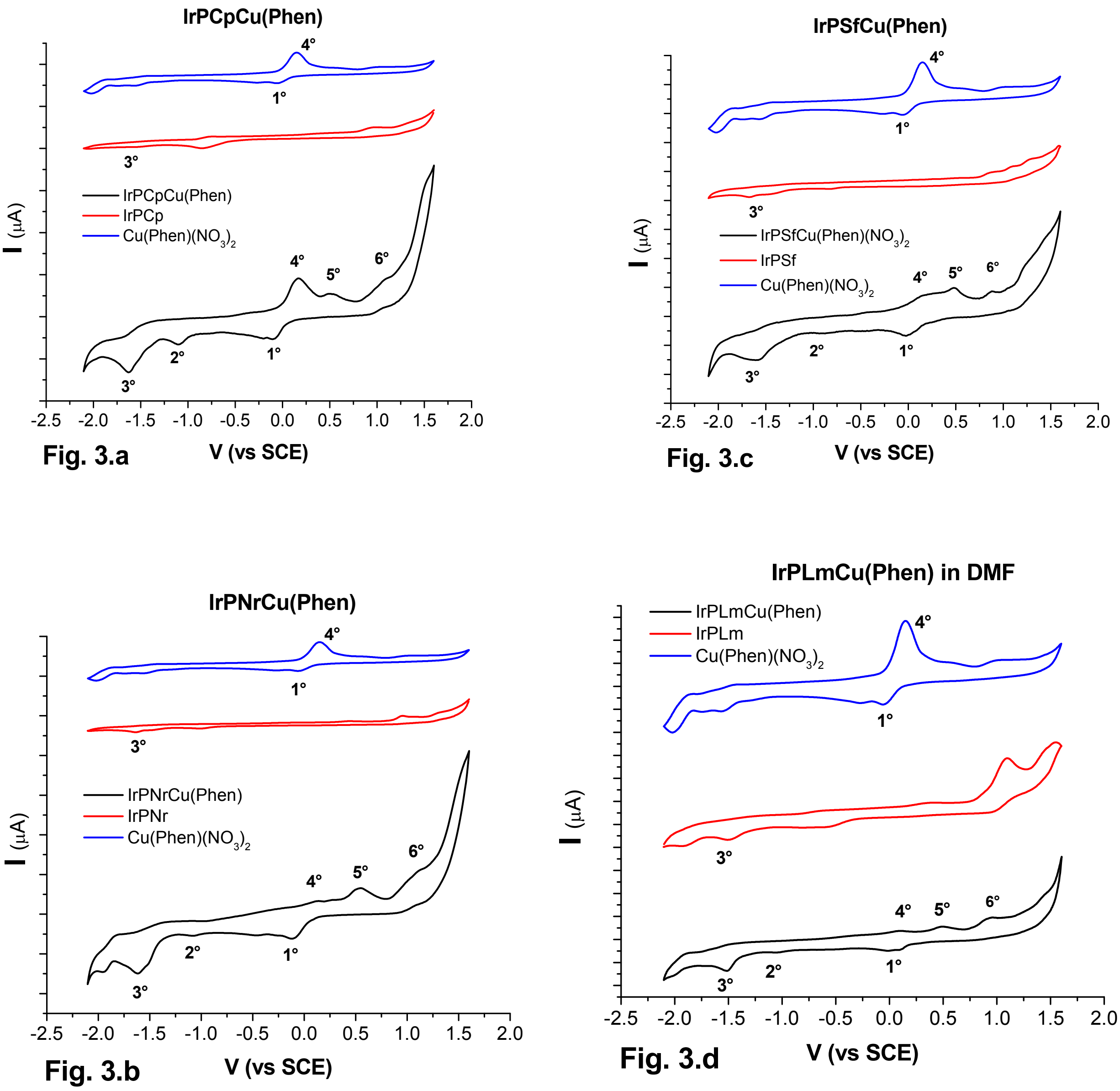

Figure S19 Cyclic voltammetry of the mononuclear fragments of Iridium complexes in DMF (50mV/s from -2.1 to +1.6 vs SCE, $TBAPF_6$ 0.1M as supporting electrolyte).

Table S3. Calculated log P values for ligands using program ACD/log P[1] and complexes using program ALOGPS 2.150.(1-3)

Ligands	Homonuclear Ir^{III}	Heteronuclear Ir^{III}-Cu^{II}

		complexes		complexes	
**PCp**	5.81	**IrPCp**	7.73	**IrPCpCu**	3.47
**PSf**	6.35	**IrPSf**	7.44	**IrPSfCu**	3.83
**PLm**	6.86	**IrPLm**	7.51	**IrPLmCu**	3.92
**PNr**	5.97	**IrPNr**	7.26	**IrPNrCu**	3.55

Table S4. Hydrodynamic diameter and Zeta potential determined by DLS technique as well as loading content and encapsulation efficiency determined by ICP-MS technique for selected liposome formulations

**Formulation**	**Diameter [nm] (TEM, ImageJ)**	**Hydrodynamic diameter [nm] (DLS)**	**Zeta potential [mV] (pH =)**
**L**	135.8 ± 21.0 nm	146.8 ±13.6, PDI = 0.01	-30.2 ± 2.7 mV
**1a**	83.7 ±18.4 nm	108.8± 24.7, PDI = 0.05	-42.3 ± 6.2 mV

Experimental methods

General procedures

All starting reagents (iridium and ruthenium), 2nd (Hcfx, Hnfx, Hlfx) and 3rd (Hsfx) generation fluoroquinolones (>98%), phosphines, $[Cu(phen)(NO_3)_2]$, $[Ir(\eta^5\text{-}Cp^*)Cl_2]_2$ and solvents were purchased from Sigma Aldrich and used without further purifications. All solvents were deaerated prior to use. Anhydrous Dimethylsulphoxide (DMSO) (34869-1L CAS: 67-68-5 ≥99.7%) and dimethylformamide (227056-1L CAS: 68-12-2 ≥99.8%) were purchased from Sigma Aldrich. Tetrabuthylammoniumhexafluorophosphate ($TBAPF_6$) was obtained from Alfa Aesar (CAS: 3109-63-5 98%). Electrodes for cyclic voltammetry were a platinum wire counter electrode (Matech Ø0.5mm 99.9%) a SCE reference (303/SCG/6) and glassy carbon disk working electrode from Amel Electrochemistry

(303/SCG/6). Mononuclear complexes (IrPsfx, IrPlfx, IrPnfx and IrPcfx) (26) and aminomethyl(diphenyl)phosphines (Psfx, (67) Plfx, (38) Pnfx (37) and Pcfx (68)) were synthesized as described previously.

Characterization of organometallic iridium(iii) complexes

X-Ray quality crystals were obtained by slow evaporation mother liquor. SCXRD measurements for 1 and 3, were performed on a Rigaku Oxford Diffraction XtaLAB Synergy-R DW diffractometer equipped with a HyPix ARC 150° Hybrid Photon Counting (HPC) detector using CuKα (λ = 1.54184 Å). For structure **2** a Xcalibur Gemini Ultra diffractometer equipped with Ruby CCD detector using CuKα (λ = 1.54184 Å) was employed. All measurements were performed at 100 K. Data were processed using the CrystAlisPro software. The structures were solved by intrinsic phasing with SHELXT (2015 release) and refined by full-matrix least-squares methods based F **2** using SHELXL. For all structures, H atoms bound to C atoms were placed in the geometrically idealized positions and treated in riding mode, with C-H = 0.95 Å and Uiso(H) = 1.2Ueq(C) for C-H groups, and C-H = 0.98 Å and Uiso(H) = 1.5Ueq(C) for CH3 groups. The N- and O-bonded hydrogen atoms which were at initial stage of refinement located from difference maps and then constrained with AFIX 13 and AFIX 3 command, respectively. Crystallographic data of the structures have been deposited at the Cambridge Crystallographic Data Centre with CCDC reference numbers 2175515: [Ir(η^5-Cp*)Cl_2Plfx-Cu(phen)](NO_3)·1.3(H_2O)·1.95(CH_3OH); 2175516: [Ir(η^5-Cp*)Cl_2Pnfx-

$Cu(phen)](NO_3)·1.75(CH_3OH)·0.75(H_2O)$ and 2175517: $([Ir(\eta^5\text{-}Cp^*)Cl_2Pcfx\text{-}Cu(phen)](NO_3)·1.75(CH_3OH)·0.75(H_2O)$.

Elemental analyses (C, H and N) were carried out with a Vario Micro Cube – Elementar. Mass spectra were recorded with a Bruker MicrOTOF-Q II spectrometer with an ESI ion source under the following conditions: nebulizer pressure: 0.4 bar, dry gas: 4.0 l min^{-1} heated to 180 °C. Data were recorded in the positive ion mode, while profile spectra were recorded in the mass range 50–3000 m/z; end plate offset −500 V; capillary voltage 4500 V; mass resolving power of the instrument – over 18 000. Mass calibration was done using the cluster method with a mixture of 10 mM sodium formate and isopropanol (1 : 1, v/v) before the run. In order to record the spectra the compounds were dissolved in chloroform.

Electronic absorption spectroscopy was carried out with an UV-Vis spectrophotometer (Agilent Technologies, Cary300 UV-Vis).

Steady state luminescence spectra were acquired with an Edinburgh Instruments FS920 spectrofluorimeter equipped with a R928 phototube detector. The spectroscopic energy E00 was obtained from the crossing of the normalized absorption and emission spectra.

FT-IR spectra of complexes were recorded using a BrukerVertex 70V vacuum spectrometer equipped with a diamond ATR cell with resolution of 2 cm^{-1} in the middle-infrared (4000-500 cm^{-1}) and far-infrared (600–

100 cm^{-1}) regions at room temperature as solid state and methanolic solutions (c = 0.80%) as well. The spectral data were collected and further elaborated using Bruker OPUS software.

Direct current (DC) magnetic measurements in the temperature range 1.8-300 K (BDC = 0.1 T) and variable - field (0-5 T) (at low temperature) were performed using a Quantum Design SQUID-based MPMSXL-5-type magnetometer. Corrections were based on subtracting the sample – holder signal and contribution χD estimated from the Pascal constants. (69) Variable-temperature (2–7 K) alternating current (AC) magnetic susceptibility measurements under different applied static fields in the range of BDC = 0–1.0 T were carried out with Quantum Design Physical Property Measurement System (PPMS). Magnetic measurements were carried out by crushing the crystals and restraining the sample in order to prevent any displacement due to its magnetic anisotropy.

Electron paramagnetic resonance (EPR) spectra were measured using a Bruker ELEXYS E 500 Spectrometer equipped with NMR teslametr and frequency counter at X-band. The experimental spectra were simulated with use of computer programs (S=1/2) written by Dr. Andrew Ozarowski from NHMFL, University of Florida. The solid compounds dissolved in water and a few drops of DMSO were added to the samples to ensure good glass formation at liquid nitrogen temperature.

Synthesis of heterometallic iridium-copper complexes

The Ir(III)/Cu(II) complexes **1–4** were prepared according to following general synthetic procedure: a solution of the [Cu(phen)(NO_3)$_2$] (1 equiv) in methanol (5 mL) was slowly added to a stirred solution of mononuclear complexes Ir(III) (1 equiv) in the same solvent (20 mL) and the mixture was stirred at room temperature in the dark. After 24 h of stirring dark-green (complexes 1-3) / -brown (complex 4) solid has been formed. The solid was filtered off, washed a few times with mixture of water and methanol (V:V = 5:1) and dried under vacuum. Green/brown solid was recrystallized from CH_3OH (T=28˚C) to give microcrystals in each case (but only complexes 1-3 were suitable for x-ray analysis).

Data for [Ir(η^5-Cp*)Cl_2Pcfx-Cu(phen)] (1)

Yield: 84%, Molar mass: 1300.196%g/mol. Anal. Calcd for $C_{52}H_{53}Cl_2CuFIrN_5O_4P\cdot(NO_3)\cdot 2.75(H_2O)$: C, 48.04; H, 4.54; N, 6.46%. Found: C, 48.05; H, 4.55; N, 6.47%.

ESI(+)MS in CH_3OH, m/z: 1129.264 (theor.: 1129.274) (100%) [IrPCpCu-~~H~~2Cl-Cl2H+$CH_{23}OH$]+; 1099.260 (theor.: 1099.263) (3%) [IrPCpCu-2Cl]+; 858.280 (theor. 858.281) (6%) [IrPCpCu-2Cl-Cu(phen)$^+$H]$^+$; 787.176 (theor.: 787.178) (27%) [IrPCpCu-$IrCl_2$+O]$^+$; 573.122 (theor.: 573.123) (9%) [IrPCpCu-$IrCl_2$-PPh_2CH_2]$^+$; 564.131 (theor.: 564.133) (5%) [IrPCpCu-2H-2Cl+$CH_{23}OH$]$^{2+}$; 549.126 (theor.: 549.127) (6%) [IrPCpCu-HCl-Cl]$^{2+}$

Data for [Ir(η^5-Cp*)Cl_2Pnfx-Cu(phen)] (2)

Yield: 89%, Molar mass: 1353.226%g/mol. Anal. Calcd for ($C_{51}H_{52}Cl_2CuFIrN_6O_6P$)·($NO_3$)·1.75($CH_4O$)·0.75($H_2O$): C, 46.82; H, 4.51; N, 7.25%. Found: C, 46.83; H, 4.52; N, 7.27%.

ESI(+)MS in CH3OH, m/z: 1157.199 (theor.: 1157.201) (100%) $[IrPNrCu+H]^+$; 1123.246 (theor.: 1123.240) (1%) $[IrPNrCu\text{-}Cl+H]^+$; 916.222 (theor.: 916.218) (4%) $[IrPNrCu\text{-}Cu+H]^+$; 759.183 (theor.: 759.183) (17%) $[IrPNrCu\text{-}IrCl_2]^+$

Data for [Ir(η^5-Cp*)Cl$_2$Plfx-Cu(phen)] (3)

Yield: 88%, Molar mass: 1357.29%g/mol. Anal. Calcd for $C_{52}H_{52}Cl_2CuF_2N_5O_3PIr$·($NO_3$)·1.3($H_2O$)·1.95($CH_4O$): C, 48.26; H, 4.81; N, 6.19%. Found: C, 48.28; H, 4.82; N, 6.20%.

ESI(+)MS in CH_3OH, m/z: 1191.175 (theor.: 1191.204) (100%) $[IrPLmCu+H]^+$; 1149.285 (theor.: 1149.280) (5%) [IrPLmCu-~~H~~2Cl-2H~~Cl~~+CH$_{23}$OH]$^+$; 948.229 (theor.: 948.225) (38%) $[IrPLmCu\text{-}Cu]^+$; 912.253 (theor.: 912.248) (5%) $[IrPLmCu\text{-}Cu(phen)\text{-}Cl]^+$; 908.303 (theor.: 908.297) (4%) [IrPLmCu-Cu(phen)-2Cl-2H+CH$_{23}$OH]$^+$; 878.293 (theor.: 878.287) (3%) $[IrPLmCu\text{-}Cu(phen)\text{-}2Cl+H]^+$; 791.191 (theor.: 791.187) (76%) $[IrPLmCu\text{-}IrCl_2]^+$; 593.131 (theor.: 593.129) (43%) $[IrPLmCu\text{-}IrCl_2\text{-}PPh_2CH_2]^+$

Data for [Ir(η^5-Cp*)Cl$_2$Psfx-Cu(phen)] (4)

Yield: 78%, Molar mass: 1231.69%g/mol. Anal. Calcd for $C_{54}H_{55}Cl_2CuF_2N_6O$¬3PIr: C, 52.66; H, 4.50; N, 6.82%. Found: C, 52.65; H, 4.49; N, 6.81%.

ESI(+)MS in CH_3OH, m/z: 1230.208 (theor.: 1230.234) (58%) $[IrPSfCu+H]^+$; 989.243(theor.: 989.251) (4%) $[IrPSfCu\text{-}Cu(phen)+H]^+$; 947.300(theor.: 947.308) (52%) $[IrPSfCu\text{-}Cu(phen)\text{-}2H\text{-}2Cl+CH_{23}OH]^+$; 919.307(theor.: 919.313) (12%) $[IrPSfCu\text{-}Cu(phen)\text{-}2Cl+H]^+$; 832.210 (theor.: 832.216) (48%) $[IrPSfCu\text{-}IrCpCl2]^+$; 634.158 (theor.: 832.156) (12%) $[IrPSfCu\text{-}IrCpCl_2\text{-}PPhCH_2]^+$; 594.651 (theor.: 594.649) (4%) $[IrPSfCu\text{-}2H\text{-}2Cl+CH_{23}OH]^{2+}$

Electrochemical characterization

Cyclic voltammetry was carried out with an Autolab PGSTAT302N potentiostat-galvanostat. A single compartment cell of the type GC/SCE/Pt containing a deoxygenated supporting electrolyte made of 0.1 M TBAPF6 in DMF was used. Typically, an analytic concentration of 0.5 mM was adopted. Voltammetric cycles were performed at 50mV/s, scanning from open circuit to negative potentials and backwards.

Preparation of liposomes

Cholesterol (100 mg) and phosphatidylcholine (200 mg) were dissolved in dichloromethane (4.5 ml). After mixing 150 µl of the solution was transferred to each Eppendorf tube (30 pieces, 1.5 ml).The solvent was blown out by nitrogen during rotating. This way liposome film was formed on the walls of tube. Each Eppendorf tube contained 10 mg of liposome. The complexes (2, 1, 0.5, 0.25 and 0.125 mg) were dissolved in dichloromethane (300 µl) and added to Eppendorf tube with liposome solution (10 mg in 150 µl of CH_2Cl_2). The solutions were homogenized in ultrasonic bath (10 min) and then blown out by nitrogen.

Water (1 ml) was poured on the film and Eppendorf tube was homogenized in ultrasonic bath to form light brown or light green liposome suspension. The samples were shaken and heated on Thermomixer comfort for 15 min at 60 °C.

TEM characterization of liposomes

Liposomes morphology was analyzed by using a FEI™ Tecnai T20 Microscope operated at 200 kV. The size was determined from the enlarged TEM micrographs, using commercially available software ImageJ, counting at least 50 particles in different images.

DLS characterization of liposomes

The average hydrodynamic diameter and electrokinetic potential were determined by the dynamic light scattering (MADLS; Zetasizer Ultra, Malwern). Measurements were performed in PBS (pH = 7.4) at room temperature.

Cell cultures

MCF7 cell line (human breast adenocarcinoma, morphology: epithelial-like, ATCC: HTB-22), A549 cell line (human lung adenocarcinoma, morphology: epithelial, ATCC: CCL-185) and HaCaT (human keratinocyte) were cultured in Dulbecco's Modified Eagle's Medium (DMEM, Corning) with phenol red, supplemented with 10% fetal bovine serum (FBS) and with 1% streptomycin/penicillin. WM266-4 cell line

(human skin (metastasis), morphology: epithelial, ATCC CRL-1676) were cultured in EMEM (EBSS) + 2mM Glutamine + 1% Non-Essential Amino Acids (NEAA) + 1% Sodium Pyruvate (NaP) + 10% Foetal Bovine Serum (FBS). DU-145 cell line (human prostate carcinoma) and HEK293T cell line (human embryonic kidney) were cultured in minimum essential medium (MEM, Corning) with only 10% fetal bovine serum (FBS). Cultures were incubated at 37 ˚C under a humidified atmosphere containing 5% CO_2. Cells were passaged using a solution containing 0.05% trypsin and 0.5 mM EDTA. All media and other ingredients were purchased from ALAB, Poland.

Cytotoxic activity

Since most of the studied compounds are insoluble in aqueous media, therefore they needed to be pre-dissolved in DMSO for biological tests. Cytotoxicity was assessed by MTT assay performed according to the protocols described previously. (70) In brief, 1×10^4 cells per well, seeded in 96-well flat-bottom microtiter plate, were incubated with the tested complexes (1, 2, 3 and 4) at various concentrations for 24 h. After that time, solutions of compounds were washed out, cells were washed thee time with PBS and fresh medium was applied. Each compound concentration was tested in five replicates and repeated at least three times. Determined values of IC_{50} (concentration of a drug required to inhibit the growth of 50% of the cells) are given as mean + S.D. (Standard Deviation). Furthermore, post-treatment survival assessment of the treated cells was analyzed under a fluorescence inverted microscope (Olympus IC51, Japan) with an excitation filter 470/20 nm. For this, cells were stained with two versatile fluorescence dyes: fluorescein

diacetate (FDA, 5 mg/mL) and propidium iodide (PI, 5 mg/mL) in standard conditions in the dark for 20 min. Before visualization dyes were removed and cells were washed with PBS twice. IC_{50} values were determined, after 72 h, from the plots of cell viability in the presence of various concentrations of each compound by matching dose–response curves.

Cellular uptake

A549 and Du-145 cells at a density of 2×10^6 cells per 2 mL were seeded on 6-well plates and were incubated with **1a** and **1b** (2mL) for 24 h (37 ˚C, 5% CO_2). Additional wells were incubated with medium alone as a negative control. Then, compound solutions were removed; the cells were washed twice with PBS buffer, and trypsinized. Measurement of the concentration of iridium ions was carried out using a mass spectrometer (ELAN 6100 PerkinElmer) with inductively coupled plasma (ICP-MS). For analysis, the collected cells were mineralized in 1 mL of 65% HNO_3 at 60˚C for 1 h. The iridium content under each condition is expressed as ng mg^{-1} protein. The protein content was assessed with Bradford Protein Assay (Thermo Scientific™). (71) The experiment was repeated at least 3 times and results are presented as mean value + S.D.

Confocal laser scanning microscopy

The intracellular uptake of **1a** was studied in the A549 and DU145 cancer cells according to the previously applied protocol. (4) In brief, before imaging, A549 and Du145 cells were seeded on microscopic slides at a

density of 1 × 10^5 cells. Cells were kept for 24 h at 37˚C in a 95% atmospheric air and 5% CO_2 humidified atmosphere. After being washed with fresh medium, the cells were incubated in the dark with 1 μM solution of 1a prepared growth medium for 2 h. Next, cells were stained with phalloidin-atto488 (Sigma Aldrich) to visualize the cytoskeleton and then incubated for 10 min with Hoechst 33342 for nuclear staining. After this incubation, at 37 ˚C, in the dark, the cells were washed with HBSS two times, and the slide was transferred to the microscope stage and cells were visualized under a confocal microscope Zeiss LSM 880 (Carl Zeiss, Jena, Germany) with a 63 × oil immersion objective. Images were analyzed by Zeiss ZEN Software.

Cell death analysis by flow cytometry

Annexin V Apoptosis Detection Kit FITC (Invitrogen) and Propidium Iodide (Thermo Fischer Scientific, Waltham, Massachusetts, USA) were used to distinguish cell death (apoptotic and necrotic cells) induced by studied compounds quantitatively due to the previously described protocol. (5) In brief, the studied compounds **1a** and **1b** (in a broad range of concertation ranging between 100 and 0.01 μM) were incubated for 24 h with A549 and HaCaT cells (seeded at density 5 × 10^5 cells/mL) in 12-well plates. After this time, the compound solutions were removed, and the cells were washed twice with PBS buffer (phosphate-buffered saline, pH = 7.4). Trypsin was added to the cells and then they were left for 10 min at 37 ˚C in a humidified atmosphere containing 5% CO_2. The cells were collected, centrifuged, and separated from the supernatant, then washed twice with 0.5 ml PBS buffer (buffer phosphate saline NaCl, KCl, Na2HPO4, KH2PO4) and suspended in Binding Buffer. Fifteen minutes before measuring, cells were stained with

Annexin V-FITC and PI and incubated in the dark. Viable and dead (early apoptotic, late apoptotic, and necrotic) cells were detected using the BDAccuri flow cytometer (BD Biosciences). The experiment was repeated at least 3 times.

Cell cycle analysis

The A549, Du145 and HaCaT cells ($3 \times 10_5$/well) were seeded in 12-well plates and treated with various concentrations of **1a**, **1b** and cisplatin (CDDP) for 24 h. Synchronization of A549, Du145 and HaCaT cell cultures was performed by serum starvation protocol. Serum starvation is widely used for synchronizing donor cells by arresting them in the G0/G1 phase of the cell cycle. (6) Cell cultures were seeded and incubated in a growth medium with 20% FBS overnight to synchronize the cell cultures. Then the cultures were rinsed by PBS and changed to serum-free medium. After serum starvation for 18 h, the cells were passaged and released into the cell cycle by the addition of serum. Then cells were treated with **1a**, **1b** and CDDP for 24 h. For FACS analysis, cell samples were harvested with trypsinization and stained with propidium iodide (20 ug/mL). Cell cycle phase distributions were analyzed by flow cytometry (BD Bioscience). Experiments were reaped at least three times.

Three-dimensional culturing *in vitro*

For hanging drop formation, the lid from a tissue culture dish was removed and 5×10^5 A549 or DU-145 cells in 10 μl drops were placed on the bottom of the lid. In each case, the cell suspension was homogeneous

and did not contain aggregates, since it determines the size and uniformity of spheroids. Then, the lid was inverted onto the PBS-filled bottom chamber and incubated at 37 ˚C/ 5% CO_2/ 95% humidity. The sphere growth was monitored daily and incubates until either cell sheets or aggregates were formed. Once sheets were formed, they were transferred to 96-well plates coated with Geltrex matrix and incubated with a completed growth medium until spheroids were created. Using optimal growth conditions, a period of 4–7 days was found to be optimal for spheroid assembly. The direct effect of drug toxicity was examined on spheroids derived from both A549 and Du-145 cell lines. For cytotoxicity assessment, spheroids were grown and were monitored for 5–7 days. After this time, the spheres were treated with the tested liposomal formulation of **1** (**1a**) at increasing doses (0.1 × IC_{50}, IC_{50}, and 10 × IC_{50}, IC_{50} determined for the corresponding Pluronic P-123 nanoformulations), and the plates were further incubated at 37 ˚C. Forty-eight hours after treatment, the spheroids were stained with 4′,6-diamidino-2-phenylindole (DAPI), calcein AM (CAM), and propidium iodide (PI) to estimate the live/dead cells population for one hour, washed and visualized using a confocal microscope Zeiss LSM 880 (Carl Zeiss, Jena, Germany) with a 10 × oil immersion objective. Images were analyzed by Zeiss ZEN Software. (7)

References

1 Petrauskas, A. A.; Kolovanov, E. ACD/Log P method description, Perspect, *Drug Discovery Des.*, **2000**, 19, 99–116

2 Tetko, V.; Gasteiger, J.; Todeschini, R.; Mauri, A.; Livingstone, D.; Ertl, P.; Palyulin, V. A.; Radchenko, E. V.; Zefirov, N. S.; Makarenko, A. S.; Tanchuk, V. Y.; Prokopenko, V. V. Virtual

computational chemistry laboratory-design and description. *J. Comput.-Aided Mol. Des.*, **2005**, 19, 453-463

3 VCCLAB, Virtual Computational Chemistry Laboratory – Design and Description, Virtual Computational Chemistry Laboratory, http://www.vcclab.org, **2005**

4 Kyzioł, A.; Cierniak, A.; Gubernator, J.; Markowski, A.; Jeżowska-Bojczuk, M.; Komarnicka, U. K. Copper(i) complexes with phosphine derived from sparfloxacin. Part III: multifaceted cell death and preliminary study of liposomal formulation of selected copper(i) complexes, Dalton Trans., 2018, 47, 1981-1992

5 Komarnicka, U. K.; Pucelik, B.; Wojtala, D.; Lesiów, M. K.; Stochel, G.; Kyzioł, A. Evaluation of anticancer activity in vitro of a stable copper(I) complex with phosphine-peptide conjugate, Sci Rep., 2021, 11, 23943

6 Chen, M.; Huang, J.; Yang, X.; Liu, B.; Zhang, W.; Huang, L.; Deng, F.; Ma, J.; Bai, Y.; Lu, R.; Huang, B.; Gao, Q.; Zhuo, Y.; Ge, J. Serum starvation induced cell cycle synchronization facilitates human somatic cells reprogramming, PLoS One, 2012, 7, e28203

7 Pucelik, B.; Arnaut, L. G.; Stochel, G.; Dabrowski, J. M. Design of Pluronic-based formulation for enhanced redaporfin-photodynamic therapy against pigmented melanoma, ACS Appl. Mater. Interfaces, 2016, 8, 22039
